# Identification of a Catalase-Phenol Oxidase in Betalain Biosynthesis in Red Amaranth (*Amaranthus cruentus*)

**DOI:** 10.3389/fpls.2015.01228

**Published:** 2016-01-08

**Authors:** Xiao-Lu Teng, Ning Chen, Xing-Guo Xiao

**Affiliations:** State Key Laboratory of Plant Physiology and Biochemistry, Department of Biochemistry and Molecular Biology, College of Biological Sciences, China Agricultural UniversityBeijing, China

**Keywords:** *Amaranthus cruentus*, betalain biosynthesis, catalase-phenol oxidase, fourth group catalase, peptide fragment, cDNA

## Abstract

Betalains are a group of nitrogen-containing pigments that color plants in most families of Caryophyllales. Their biosynthesis has long been proposed to begin with hydroxylation of L-tyrosine to L-DOPA through monophenolase activity of tyrosinase, but biochemical evidence *in vivo* remains lacking. Here we report that a Group 4 catalase, catalase-phenol oxidase (named as AcCATPO), was identified, purified and characterized from leaves of *Amaranthus cruentus*, a betalain plant. The purified enzyme appeared to be a homotrimeric protein composed of subunits of about 58 kDa, and demonstrated not only the catalase activity toward H_2_O_2_, but also the monophenolase activity toward L-tyrosine and diphenolase activity toward L-DOPA. Its catalase and phenol oxidase activities were inhibited by common classic catalase and tyrosinase inhibitors, respectively. All its peptide fragments identified by nano-LC-MS/MS were targeted to catalases, and matched with a cDNA-encoded polypeptide which contains both classic catalase and phenol oxidase active sites. These sites were also present in catalases of non-betalain plants analyzed. *AcCATPO* transcript abundance was positively correlated with the ratio of betaxanthin to betacyanin in both green and red leaf sectors of *A. tricolor*. These data shows that the fourth group catalase, catalase-phenol oxidase, is present in plant, and might be involved in betaxanthin biosynthesis.

## Introduction

Betalains are vacuole-localized, water-soluble, nitrogen-containing pigments, which consist of red-violet betacyanins and yellow-orange betaxanthins (Gandía-Herrero et al., 2005; Hatlestad et al., 2012). They are distributed in plants belonging to most families of Caryophyllales and some higher fungi such as *Amanita muscaria* (Musso, 1979; Cuénoud et al., 2002; Strack et al., 2003), and responsible for the bright and beautiful coloration of the plants (Wang et al., 2007a; Castellanos-Santiago and Yahia, 2008; Svenson et al., 2008; Gandía-Herrero et al., 2009). Betalains are used in food industry such as food additives (Stintzing and Carle, 2004) and recognized as compounds with potential health-benefits because of their antioxidant and radical scavenging properties (Kanner et al., 2001; Stintzing and Carle, 2004; Tesoriere et al., 2004; Han et al., 2009; Gandía-Herrero et al., 2014). Thus, how betalains are biosynthesized has attracted great attention of researchers.

**Figure 1:**
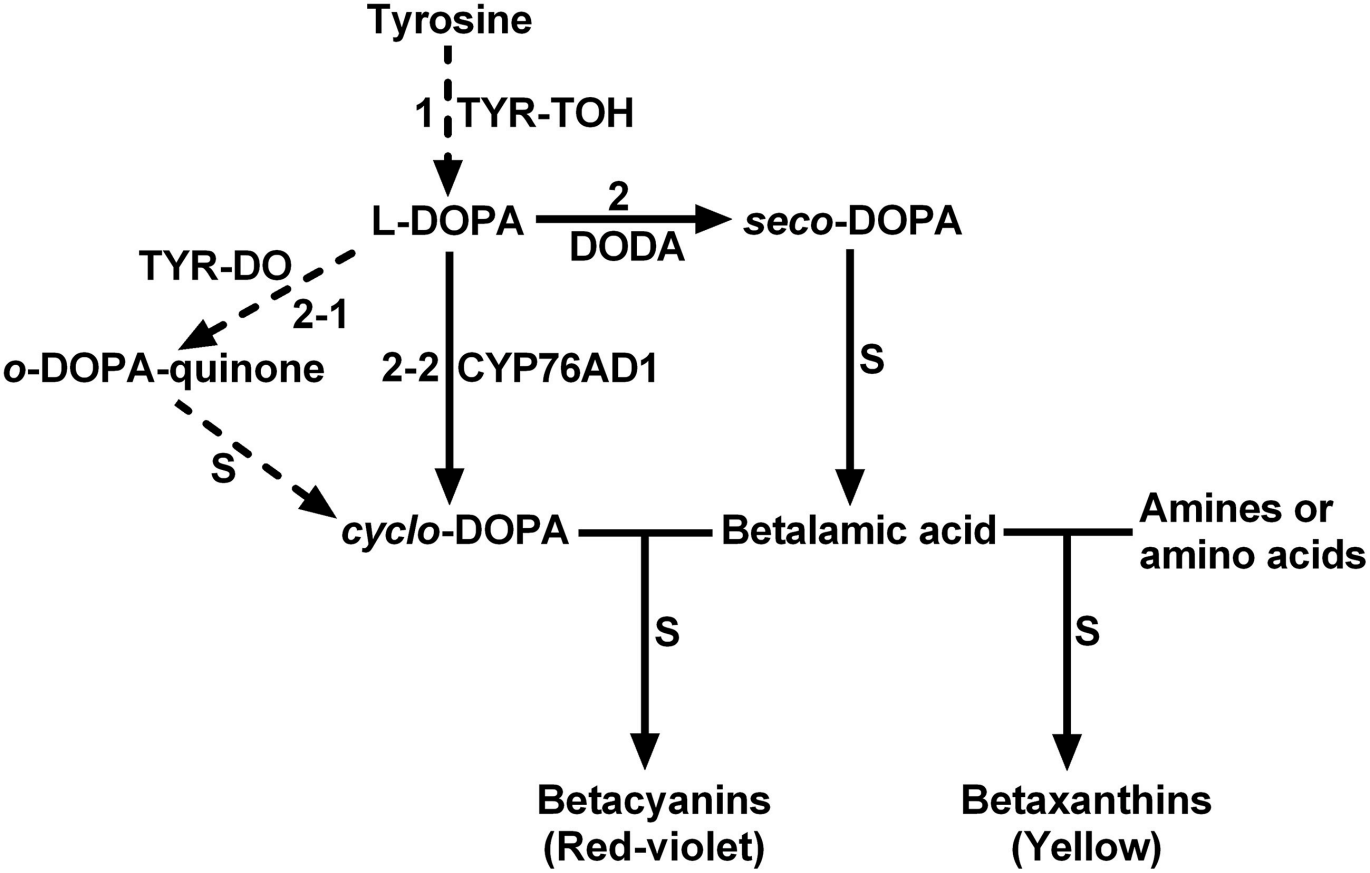
**The schematic description showing proposed key steps of plant betalain biosynthetic pathway**. S, spontaneous reactions; TYR-TOH, tyrosine hydroxylase or monophenolase activity of tyrosinase; TYR-DO, diphenolase activity of tyrosinase; CYP76AD1, a cytochrome P450 enzyme; DODA, 4,5-DOPA-extradiol dioxygenase. Solid arrows show biochemically and molecularly confirmed enzymatic reactions. Dashed arrows show proposed reactions.

The biosynthetic pathway of betalains is presumed to begin with the hydroxylation of L-tyrosine to L-DOPA only through monophenolase activity of tyrosinase (Figure 1, step 1) (Strack et al., 2003; Grotewold, 2006; Gandía-Herrero and García-Carmona, 2013). In addition to this first and crucial step, tyrosinase is also proposed involved in several other steps of the biosynthetic pathway such as oxidation of L-DOPA (Figure 1, step 2-1) (Strack et al., 2003). The classical tyrosinase is considered to be a bifunctional polyphenol oxidase (PPO) (Strack and Schliemann, 2001). It catalyzes two distinct and continuous reactions in the presence of molecular oxygen: the hydroxylation of monophenols to *o*-diphenols (EC 1.14.18.1; tyrosine hydroxylase activity or monophenolase activity) and their subsequent oxidation to the corresponding *o*-quinones (EC 1.10.3.1; diphenolase activity). Between two functions, the first one, *i.e*., monophenolase activity is known to be initial and rate-determining (Rodríguez-López et al., 1992; Selinheimo et al., 2007), in particular toward L-tyrosine. Therefore, the monophenolase activity is a key feature for distinguishing tyrosinases involved in biosynthesis of specialized metabolites such as betalains from other PPOs associated with plant browning and defense (Strack and Schliemann, 2001; Sullivan, 2015).

Possible involvement and important roles of tyrosinase in betalain biosynthesis have promoted its identification, purification and biochemical characterization. A tyrosinase was initially purified and characterized from the betalain-forming pileus of the fungus *Amanita muscaria*, and both monophenolase and diphenolase activities were detected (Mueller et al., 1996). However, the substrate used for detecting the monophenolase activity was L-tyramine but not L-tyrosine, the typical substrate for tyrosinase (Chang, 2009). In betalain-forming plants, the tyrosinases with monophenolase activity to L-tyrosine and diphenolase activity toward L-DOPA were partially or highly purified and more or less characterized from betacyanin-producing cell- or callus cultures of *Portulaca grandiflora* (Steiner et al., 1999; Yamamoto et al., 2001). The question remaining to be answered is whether the tyrosinase from these cell- and callus cultures can well represent the tyrosinase from plant tissues or organs. From beet root a tyrosinase was purified and its diphenolase activity toward L-DOPA was characterized, but its monophenolase activity was detected only with L-tyramine as substrate in gel staining (Gandía-Herrero et al., 2004). In 2007, Wang and his colleagues partially purified a tyrosinase with diphenolase activity to L-DOPA and some monophenolase activity toward L-tyrosine from cotyledons of dark-grown *Suaeda salsa* seedlings, and showed that the tyrosinase activity was positively correlated with betalain biosynthesis (Wang et al., 2007b). Our group previously highly purified and characterized a tyrosinase from leaves of red Swiss chard (*Beta vulgaris* subsp. *cicla*), but the monophenolase activity of purified tyrosinase toward L-tyrosine was much lower than its diphenolase activity to L-DOPA (Gao et al., 2009). Lacking or very low monophenolase activity toward L-tyrosine of purified tyrosinases from plant tissues or organs raises an intriguing question: how can so low monophenolase activity of the tyrosinase toward L-tyrosine provide sufficient L-DOPA for sequentially synthesizing large amount of betalains, such as betacyanins in red Swiss chard? Two possible explanations may exist: (1) the tyrosinases purified up-to-now are not the tyrosinases involved in betalains biosynthesis. (2) there exist other enzymes rather than PPO-type tyrosinase responsible for conversion of L-tyrosine to L-DOPA. Recently CYP76AD1, a new cytochrome P450 was found to convert L-DOPA into *cyclo*-DOPA, replacing the diphenolase activity of proposed tyrosinases, and thus involved in betalain biosynthesis of sugar beet (Hatlestad et al., 2012). Accordingly and logically, the second explanation is supported.

Keeping the second explanation in mind, we purified and biochemically characterized an enzyme with both catalase activity and tyrosinase activity from leaves of red amaranth (*Amaranthus cruentus*), a C_4_ betalain-producing plant. This new type of catalase, catalase-phenol oxidase (CATPO), might be involved in betalain biosynthesis, and in particular in betaxanthin biosynthesis.

## Materials and methods

### Plant materials

Red amaranth plants (*Amaranthus cruentus* cv. Hopi Red Dye) were grown in greenhouse with natural light in summer, and common amaranth plants (*Amaranthus tricolor*) were purchased from a local market. Fresh young red-violet leaves of red amaranth were washed, cut and processed for enzyme and RNA extraction immediately, and fresh young tricolor leaves of common amaranth were washed, sectioned based on the color and separated for betalain content and quantitative Real-Time PCR (qRT-PCR) analysis.

### Enzyme extraction and purification

Fresh young red-violet leaves were homogenized with ice-cold 50 mM sodium phosphate buffer (pH 7.0) containing 10 mM EDTA, 10 mM mercaptoethanol, 0.1% (w/v) SDS, 0.1% (v/v) Triton X-100, and 40% (v/v) methanol. The samples were filtered through six layers of cheesecloth, centrifuged twice at 4°C at 8000 g for 30 min to remove plant debris. The supernatant (crude extract) obtained was stored at −80°C for further purification. The crude extract was purified through gel slice according to McMahon et al. (2007) with small modifications. After native-PAGE electrophoresis (63 mA for 7 h at 4°C; 20 ^*^ 20 cm; JunYi, Beijing), a single lane of the gel was stained with 5 mM L-DOPA for 60 min at 45°C. Using the stained lane as a marker to the position of enzyme with diphenolase activity, the corresponding band was sliced out of the unstained lanes, excised and extracted in ice-cold 50 mM Tris-HCl buffer (pH 7.0). Purified enzyme (supernatant) was obtained by centrifugation and stored at −80°C. The enzymatic activities of the purified enzymes in different sets were detected according to in-gel staining of diphenolase activity (Wang and Constabel, 2003), and the enzyme solutions of different sets with high enzymatic activity and a single band of same size were pooled together for further characterization studies. The protein content was determined according to the Bradford method (Bradford, 1976).

### SDS-PAGE, native PAGE and enzyme staining

SDS-PAGE was carried out on discontinuous gels (10% separating gel, 20 μL purified enzyme or 8 μL crude extract loaded on each lane) according to the method of Laemmli (1970). Native PAGE was performed as described in Nellaiappan and Vinayagam (1986) and Beck et al. (1996). The native PAGE gel or SDS-PAGE gel was stained for protein purity and size by using Coomassie Brilliant Blue (CBB) and/or silver.

### Monophenolase activity assays of purified enzyme

Monophenolase activity toward L-tyrosine of the purified enzyme was first qualitatively estimated by color change in reaction mixture. The reaction mixture (1 mL) contained 25 mM Tris-HCl buffer (pH 7.0), 2.5 mM L-tyrosine and 1 μg mL^−1^ enzyme, and was placed at room temperature until reddish color appeared. A control (CK) was performed in parallel without the enzyme. Then the influence of cofactor L-DOPA on the reaction speed of monophenolase catalysis was tested according to Espín and Wichers (2001) with some modifications. Two concentrations of L-DOPA, 0.0114 mM ([L-DOPA]/[L-tyrosine] = 0.057) and 0.057 mM, were used and the positive controls were set with commercial tyrosinase from non-betalain-forming mushroom *Agaricus bisporus*. The reaction solution (200 μL) consisted of 50 mM potassium phosphate buffer (pH 7.0), 50 μM CuSO_4_, 0.2 mM L-tyrosine (Winder and Harris, 1991), 0.0114 or 0.057 mM L-DOPA. The negative control was carried out without the enzyme. After addition of 2 μg mL^−1^ enzyme to the reaction solution, the absorbance was immediately read at 475 nm every 15 min for 3 h (37°C) in the Microplate Spectrophotometer PowerWave XS2 (BioTek Instruments, USA). Unless otherwise stated, experiments were repeated in triplicate. After determining the suitable cofactor concentration, the monophenolase activity was quantitatively determined spectrophotometrically by measuring dopaquinone accumulation at 475 nm (Ros et al., 1994; Espín and Wichers, 2001) under the previous assay condition with L-tyrosine as substrate and 0.057 mM L-DOPA as cofactor. The reaction mixtures were incubated at the optimal temperature 60°C for 90 min and the absorbance was immediately measured. One unit of the monophenolase of tyrosinase was defined as the amount of the enzyme that produces 1 μmol of dopaquinone (the molar extinction coefficient: 3600 M^−1^ cm^−1^) per minute, and the monophenolase activity toward L-tyrosine was calculated after removing the effect of L-DOPA as substrate.

Effects of pH and temperature on the monophenolase activity of the purified enzyme were evaluated under the above assay condition. Optimal pH for the monophenolase activity was estimated by monitoring the enzyme's activity at pH range of 4.0–12.0. The buffer systems (at final concentration of 50 mM) used were acetate buffer for pH 4.0–5.0; phosphate buffer for pH 6.0–7.0; Tris-HCl buffer for pH 8.0–9.0; carbonate-bicarbonate buffer for pH 10.0–11.0; biphosphate-hydroxide buffer for pH 12.0. For determining the optimal temperature, the enzyme activity was measured at temperature ranging from 20 to 80°C. At the optimal temperature and pH, the *K*_*m*_ value for L-tyrosine was estimated by measuring dopaquinone formation rate from L-tyrosine at concentrations of 0.1, 0.125, 0.15, 0.1675, 0.25, 0.3, and 1 mM under previous assay conditions.

Effects of six common tyrosinase inhibitors (tropolone; kojic acid; sodium diethyldithiocarbamate, SDDC; phenylthiocarbamide; EDTA; sodium azide; final concentration, 1 mM) on the monophenolase activity was evaluated under the previous assay condition without the addition of CuSO_4_. A control test was run in parallel in the absence of the inhibitor.

### Diphenolase activity assays of purified enzyme

Diphenolase activity of the purified enzyme was assayed with L-DOPA as substrate by measuring the dopaquinone accumulation at 475 nm. The reaction mixture (200 μL) contained 50 mM Tris-HCl (pH 8.0), 1 mM L-DOPA, and 2 μg mL^−1^ enzyme. The reaction mixtures were incubated at the optimal temperature 60°C for 60 min and the absorbance was measured at 475 nm. The control was performed in parallel without the enzyme. One unit of the diphenolase of tyrosinase was defined as the amount of the enzyme that produces 1 μmol of dopaquinone per minute.

The effects of pH and temperature on the diphenolase activity were evaluated as on the monophenolase activity, except L-DOPA instead of L-tyrosine as the substrate. The *K*_*m*_ value for L-DOPA was calculated by measuring dopaquinone formation rate from L-DOPA at eight concentrations (0.1, 0.125, 0.2, 0.25, 0.5, 0.8, 1.0, 1.5 mM) at the optimal temperature and pH.

The effects of five common tyrosinase inhibitors [tropolone; kojic acid; sodium diethyldithiocarbamate (SDDC); phenylthiocarbamide; EDTA; final concentration, 1 mM] on the diphenolase activity of the purified enzyme were evaluated through both in-gel staining and spectrophotometer. For in-gel staining determination, each three lanes of the gel, after electrophoresis on native PAGE with the purified enzyme (40 μL on every lane) were sliced out and incubated in 5 mM L-DOPA containing different inhibitors for 60 min at 45°C. A control test was run in parallel without the inhibitor. For spectrophotometry, the diphenolase activity was measured in the standard reaction medium in the presence or absence of the stated concentration of inhibitor.

### Nano-LC-MS/MS analysis and database searches

After SDS-PAGE, the band corresponding to the purified enzyme was excised from the gel, destained, and digested with sequencing-grade modified trypsin (Roche). Nano-LC-MS/MS analysis was conducted by using a Q-Exactive system (Thermo Scientific). The peptide mixture was separated using a nanoAcquity Ultra performance LC (Waters). A sample volume of 10 μL was loaded, and separation was performed at a flow rate of 200 nL min^−1^ using 0.1% formic acid (v/v; solvent A) and 0.1% formic acid in acetonitrile (v/v; solvent B). The HPLC linear gradient for separation was 1–90% (v/v). A spray voltage of 2 kV was applied. The MS scan range was mass-to-charge ratio 300–1800, and the 10 most intense precursor ions were selected for subsequent MS/MS scans. The MASCOT Server 2.4 (Matrix Science) software was used for MASCOT database searching. Peptide data were searched against the NCBI non-redundant database (Nov. 19, 2013). The type of search was MS/MS Ion Search with 2+ as peptide charge and Viridiplantae (Green Plants) as taxonomy, and the instrument type was ESI-TRAP. Up to two missed cleavage was allowed, and mass tolerance for protein identification was 10 ppm for MS and 20 mmu for MS/MS. Peptides were considered identified if the MASCOT score was over the 95% confidence limit based on the “identity” score of each peptide.

### Catalase activity assays of purified enzyme

Catalase activity of the purified enzyme was studied through *in vitro* catalysis and inhibition assays. For catalysis assays, two methods were used: in-gel activity assay and spectrophotometer. After concentrating 100-folds by ultrafiltration (Amicon Ultra-4 30 K MWCO centrifugal filter units, Millipore) (Bollag and Edelstein, 1991), the purified enzyme was subjected to the in-gel activity assay. The assay was carried out according to Weydert and Cullen (2010) with small modifications: rinse the gel with ddH_2_O only once for 5 min after incubating the gel in the H_2_O_2_. The spectrophotometer measurement of the catalase activity was performed by monitoring the decrease in absorbance at 240 nm of the reaction mixture as described in Merle et al. (2007). The reaction mixture (200 μL) consisted of 14 mM H_2_O_2_ in 50 mM potassium phosphate buffer (pH 7.0), and 2 μg mL^−1^ enzyme. Catalase unit, defined as the amount of enzyme that catalyzes the decomposition of 1 μmol H_2_O_2_ per minute, was calculated using a molar extinction coefficient of 39.4 M^−1^ cm^−1^ for H_2_O_2_ at 240 nm. For *in vitro* inhibition assays, the residual catalase activity was measured by spectrophotometer in the presence of each of four common catalase inhibitors (isoniazid, sodium dithionite, sodium azide, and salicylic acid) at 1 mM final concentration under the standard assay condition, and with absence of the inhibitor as control.

The optimal pH and temperature for the catalase activity was estimated as those for the tyrosinase activity except the temperature ranging from 10 to 60°C and H_2_O_2_ as the substrate. At the optimal temperature and pH, the *K*_*m*_ value for the catalase activity was determined using H_2_O_2_ at eight concentrations (1.6, 2, 4, 5, 8, 10, 14 and 20 mM) under standard assay conditions.

### cDNA cloning and sequence analysis

A reverse cloning approach was used based on nano-LC-MS/MS-peptides matched *Mesembryanthemum crystallinum* catalase and *Suaeda maritima* subsp. *salsa* catalase to clone the cDNA of the purified enzyme. Total RNA was extracted from fresh young red-violet leaves of red amaranth with Trizol reagent (Invitrogen) following the manufacturer's protocols. First strand cDNA was obtained from 4 μg of the RNA using a PrimeScript II 1^st^ strand cDNA Synthesis Kit (TaKaRa) according to the manufacturer's instructions. A core cDNA fragment was PCR-amplified with degenerate primers AcCATPOF1 and AcCATPOR1 (Table 1) and Taq DNA polymerase (TIANGEN, China) by denaturing for 30 s at 94°C, then annealing for 30 s at 61.5°C, extension for 2 min at 72°C for 30 cycles and a final extension for 10 min at 72°C. The PCR product of *ca*.1500 bp was gel purified with the AxyPrep DNA gel extraction kit (Axygen, USA), cloned into the pGEM-T vector (Promega) and sequenced at BGI Inc. (China) (The same for following PCR products). Subsequently, the 5′-end and 3′-end of the core sequence were PCR-extended with RACE technique by using Ex Taq™ DNA polymerase (TaKaRa), 5′/3′ Full RACE kit (TaKaRa), two 5′-end gene-specific primers (5′ GSP I and 5′ GSP II, Table 1) and two 3′-end ones (3′GSP I and 3′GSP II, Table 1), respectively. The PCR was run as described above except annealing at 56°C for 5′RACE and 69°C for 3′RACE, and the products were cloned and sequenced. Based on the assembled sequence of the core and RACE fragments, an entire coding sequence of 1479 bp was PCR-amplified by using a specific primer pair AcCATPO-F/AcCATPO-R (Table 1) with an annealing temperature of 65°C, and cloned and sequenced. Multiple alignments of cDNAs and their deductively encoded protein sequences were performed with DNAMAN (version 6.0).

**Table 1:** **Primers used in this study**.

**Primer name**	**Primer sequence (5′ → 3′)**	**Purpose**
AcCATPOF1	ATGGATCCATACAAGTACCGAC	Cloning cDNA of the purified enzyme
AcCATPOR1	CTTCACATGGTTGGCCTCAC	
3′ GSP I	CCATCAAGATTTGATCCAGTTCGTGAGGCAG	
3′ GSP II	CAAGGAACCAGGGGAGAGATACAG	
5′ GSP I	AGAACACCGGGAAGTTGTTACCTACTATG	
5′ GSP II	AGCACTTGCACCTCTAGCATG	
AcCATPO-F	ATGGATCCTTACAAGTATCGGCCTTCAAGTG	
AcCATPO-R	TTCACATGGTTGGTCTTATGTTAAGTC	
qAt-Actin-1F	CGTGACCTGACTGATTACCCTA	qRT-PCR
qAt-Actin-1R	ACCTCAGGGCAACGGAAT	
q-AcCATPO-1F	CCCCAGAGGTCCGATTC	
q-AcCATPO-1R	CGCAAGTGAGATGGGAAACG	

The cDNA-encoded protein sequence was first compared with that of CATPOs reported for catalase and phenol oxidase active sites analysis, and then multiply aligned with those retrieved from a basic local alignment search (BLAST) in GenBank by using itself as a query for phylogenetic analysis. The multiple sequence alignment was performed by using ClustalW2 and the unrooted tree was constructed with the software MEGA 6.0 using the neighbor-joining algorithm.

### Betalain pigment analysis

Green and red sectors of young leaves (8–10 leaves) of *A. tricolor* were carefully separated and pooled respectively. The pooled sample of 200–300 mg (fresh weight) was homogenized to a fine powder in liquid nitrogen and then the betalains were extracted in 1 ml of 80% (v/v) methanol containing 50 mM sodium ascorbate as described in Harris et al. (2012). Betacyanins and betaxanthins were detected spectrophotometrically at 538 and 470 nm and quantified by using the molar extinction coefficient 60,000 and 48,000 M^−1^ cm^−1^, respectively.

### Quantitative real-time PCR (qRT-PCR)

Total RNA was extracted from green and red leaf sectors (green or red) of *A. tricolor* as described above. Approximately 1 μg RNA was digested using RNase-free DNase I (Promega) at 37°C and then reverse-transcribed to cDNA using a PrimeScript II 1^st^ strand cDNA Synthesis Kit (TaKaRa). The qRT-PCR was conducted in a reaction mixture of cDNA, SYBR Green Master Mix (TaKaRa) and gene-specific primer pair (q-AcCATPO-1F/q-AcCATPO-1R, Table 1) with *Actin* (GenBank accession number: 156972026, see Table 1 for primers) as an internal control. The qRT-PCR was performed using a qTOWER 2.2 real-time PCR system (Analytik Jena).

## Results

### Purification of enzymes with tyrosinase activity from leaves of red amaranth

To purify enzymes with tyrosinase activity from red–violet leaves of red amaranth quickly, the crude enzymes were separated on Native-PAGE and actively stained with L-DOPA (for the diphenolase activity of tyrosinase). Each of stained bands was gel-sliced out of the gel, and the second band was selected for further studies after preliminary test. This gel slice purification not only removed large amount of red–violet pigments betacyanins, but also gave an overall purification of 7.5-fold, with an overall activity yield of *ca*. 10.5% and the specific activity of 0.0177, 0.2011, and 230.7 U mg^−1^ protein for monophenolase, diphenolase and catalase, respectively (Table 2).

**Table 2:** **Purification of AcCATPO from leaves of red amaranth**.

**Purification step**	**Volume (mL)**	**Total activity (U)**			**Total protein (mg)**	**Specific activity (U/mg)**			**Yield (%)**			**Purification fold**		
		**CAT**	**MO**	**DO**		**CAT**	**MO**	**DO**	**CAT**	**MO**	**DO**	**CAT**	**MO**	**DO**
Crude extract	40	1150	0.082	0.9448	35.65	32.3	0.0023	0.0265	100	100	100	1	1	1
Gel slice	46	116	0.0089	0.101	0.5	230.7	0.0177	0.2011	10.1	10.8	10.7	7.2	7.7	7.6

*CAT, catalase activity; MO, monophenolase activity; DO, diphenolase activity. Each specific activity was measured under its optimal conditions. Optimal Temperature 60°C and pH 8.0 for MO and DO; Optimal Temperature 20°C and pH 7.0 for CAT*.

**Figure 2:**
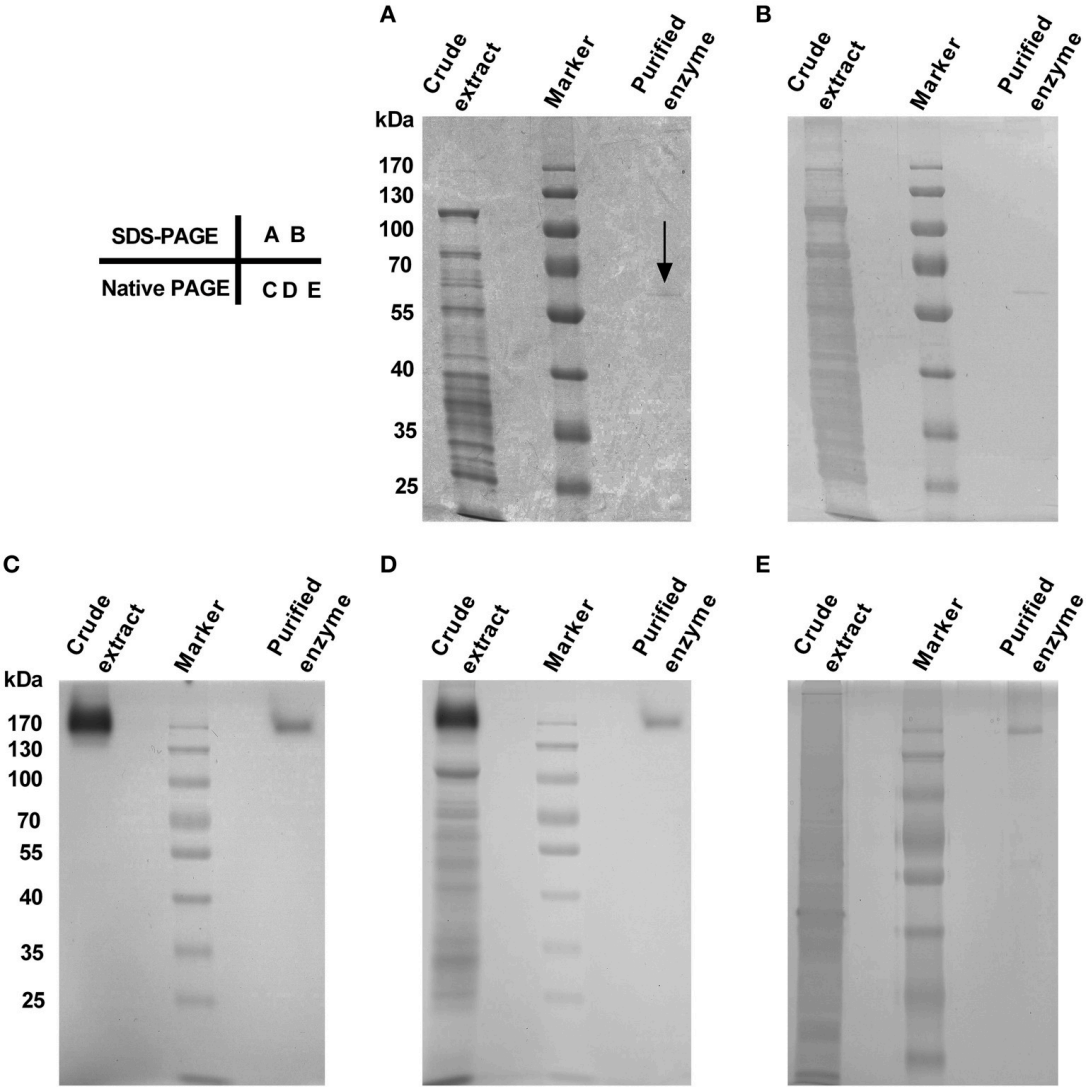
**Purity and molecular mass of gel slice-purified enzyme from leaves of red amaranth**. **(A)** 10% SDS-PAGE separation and Coomassie Brilliant Blue (CBB) staining of crude extract (8 μL) and the purified enzyme (20 μL). The arrow showed the position of the purified enzyme. **(B)** Silver staining after discoloration of **(A)**. **(C)** 10% native PAGE separation and in-gel staining of crude extract (12.5 μL) and the purified enzyme (34 μL) with 5 mM L-DOPA at 45°C. **(D)** CBB staining after wash of **(C)** with ddH_2_O for 5 min. **(E)** 10% native PAGE separation and silver staining of crude extract (5 μL) and the purified enzyme (15 μL). The same prestained protein marker was also used in **(B–E)**.

The purified enzyme appeared as a single band both on SDS-PAGE and on Native PAGE stained with Coomassie Brilliant Blue (CCB) and/or silver (Figure 2), but the band size varied, being estimated *ca*. 58 kDa on SDS-PAGE (Figures 2A,B) and about 170 kDa on Native PAGE (Figures 2C–E). These data signified that the enzyme was purified to apparent homogeneity, and the purified enzyme appeared to be a homotrimeric protein composed of subunits of about 58 kDa.

### Characterization of the monophenolase activity of purified enzyme

**Figure 3:**
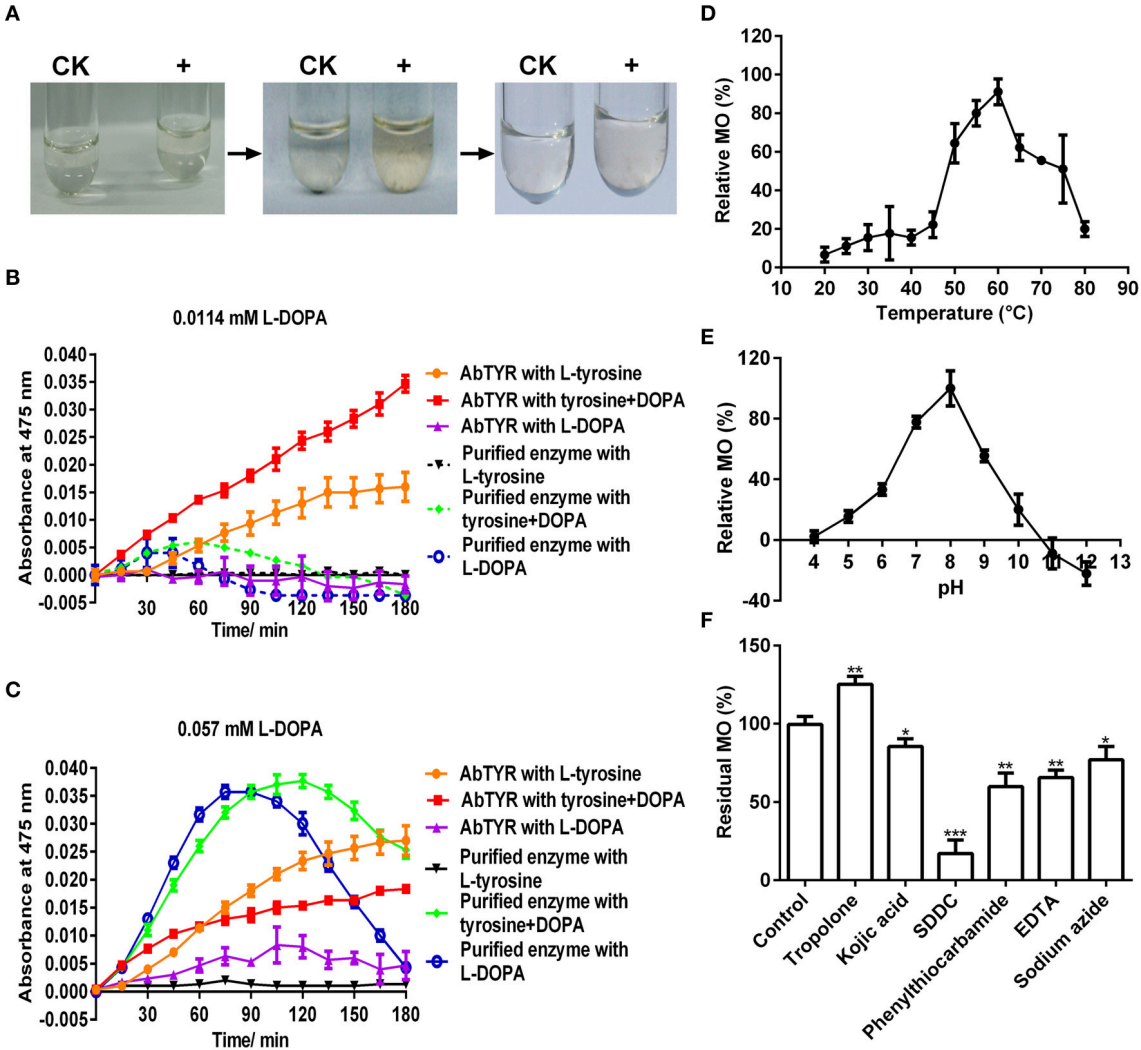
**Biochemical characterization of the monophenolase activity of the purified enzyme**. **(A)** *In vitro* catalysis of L-tyrosine with the purified enzyme. The color changes were showed in the reaction mixture containing 25 mM Tris-HCl buffer (pH 7.0), 2.5 mM L-tyrosine and 0 (CK) or 1 μg mL^−1^ enzyme (+). **(B)** Monophenolase activity assay of the purified enzyme with 0.0114 mM L-DOPA as cofactor and mushroom (*Agaricus bisporus*) tyrosinase as positive control. AbTYR: tyrosinase from *A. bisporus*. The reaction mixture (200 μL) consisted of 50 mM potassium phosphate buffer (pH 7.0), 50 μM CuSO_4_, 2 μg mL^−1^ enzyme, 0.2 mM L-tyrosine and/or 0.0114 mM L-DOPA. The negative control (without the enzyme) was used as blank for spectrophotometer analysis. After addition of the enzyme, the absorbance of the reaction mixture was immediately read at 475 nm every 15 min for 3 h (37°C) in the Microplate Spectrophotometer PowerWave XS2 (BioTek Instruments, USA). **(C)** Monophenolase activity assay of the purified enzyme with 0.057 mM L-DOPA as cofactor and mushroom tyrosinase as positive control. The reaction conditions were identical to **(B)**. **(D)** Effects of temperature on the monophenolase activity (MO) toward L-tyrosine. **(E)** Effects of pH on the MO toward L-tyrosine. **(F)** Effects of inhibitors (final concentration, 1 mM) on the MO toward L-tyrosine. SDDC, sodium diethyldithiocarbamate. Data are from three independent experiments (mean ± SD). Statistical significance was analyzed by Student's *t*-test with the control; ^*^P < 0.05; ^**^P < 0.01; ^***^P < 0.001.

To test if the purified enzyme possessed monophenolase activity, we first qualitatively observed color changes in catalyzing reaction mixtures with L-tyrosine as substrate. After addition of the purified enzyme, the colorless reaction mixtures became light reddish slowly, and then turned to red-black rapidly (Figure 3A), which is consistent with the characteristic reaction of tyrosinase (Sánchez-Ferrer et al., 1995; Espín and Wichers, 2001). In order to speed up the first color reaction, we added 0.0114 or 0.057 mM of L-DOPA, the cofactor of tyrosinase (Pomerant and Warner, 1967), and used commercial *Agaricus bisporus* tyrosinase as positive control. Without cofactor, the purified enzyme displayed very low monophenolase activity toward L-tyrosine, whereas the mushroom tyrosinase showed much higher activity (Figures 3B,C). With 0.0114 mM L-DOPA as cofactor, monophenolase activity of the purified enzyme was not obviously increased, but that of mushroom tyrosinase markedly enhanced (Figure 3B). With 0.057 mM cofactor, a significant increment of monophenolase activity of the purified enzyme was observed during all detection duration of 180 min (Figure 3C), while for the mushroom tyrosinase, a rapid increased monophenolase activity was noted during first 60 min, and from 60 to 180 min, the monophenolase activity became lower than that without cofactor (Figure 3C). Therefore, 0.057 mM L-DOPA and 90 min were used as “standardized conditions” in subsequent monophenolase activity assay of the purified enzyme.

It was also noted that the purified enzyme exhibited apparently higher activity toward L-DOPA alone than to L-DOPA plus L-tyrosine during the first 90 min, but after that, it went opposite (Figure 3C). This suggested that during the first 90 min, the L-DOPA was used mainly as the cofactor and after-then as substrate of the purified enzyme in monophenolase activity assay. Similar roles of the L-DOPA were showed in the monophenolase activity assay of mushroom tyrosinase during and after 60 min (Figure 3C).

The effect of temperature on the monophenolase activity of the purified enzyme was tested at a temperature range of 20–80°C under standardized conditions. The monophenolase activity arose with the increment of temperature until 60°C, and higher than 60°C, it dropped rapidly and only less than 20% residual activity remained at 80°C (Figure 3D).

The effect of pH on the monophenolase activity of the purified enzyme was also examined. Below pH 6.0, the monophenolase showed very weak activity, while from pH 6.0 to pH 8.0, its activity arose rapidly and arrived at maximum at pH 8.0 (Figure 3E). The activity then decreased with the increment of pH and at pH 10.0, it remained less than 20%.

Similarly, the effect of six common tyrosinase inhibitors (final concentration, 1 mM) on the monophenolase activity of the purified enzyme was investigated. As for classic tyrosinase, all inhibitors except tropolone, had a significantly inhibitory effect on the monophenolase activity (Figure 3F). Sodium diethyldithiocarbamate (SDDC), a Cu^2+^ chelator, demonstrated the strongest inhibitory effect, leading to approximately 80% loss of activity. Kojic acid, a standard inhibitor for tyrosinase (Chang, 2009), displayed an inhibitory impact slightly lower than that of EDTA and phenylthiocarbamide, resulting in about 15% decrease in enzymatic activity. Surprisingly, tropolone, a slow-binding inhibitor for classic tyrosinase (Espín and Wichers, 1999) showed a significantly stimulating effect on the monophenolase activity, bringing about 25% increase of the activity (Figure 3F).

At the optimal temperature 60°C and pH 8.0, a *K*_*m*_ value of 0.2 mM toward L-tyrosine was calculated from the Lineweaver-Burk graphs for the purified enzyme (Supplementary Data 1).

### Characterization of the diphenolase activity of purified enzyme

L-DOPA active staining of the Native PAGE (Figure 2C) and the cofactor experiment for monophenolase activity of the purified enzyme already demonstrated the diphenolase activity of the purified enzyme. Here we examined effects of temperature, pH and common tyrosinase inhibitors on the diphenolase activity with L-DOPA as substrate.

**Figure 4:**
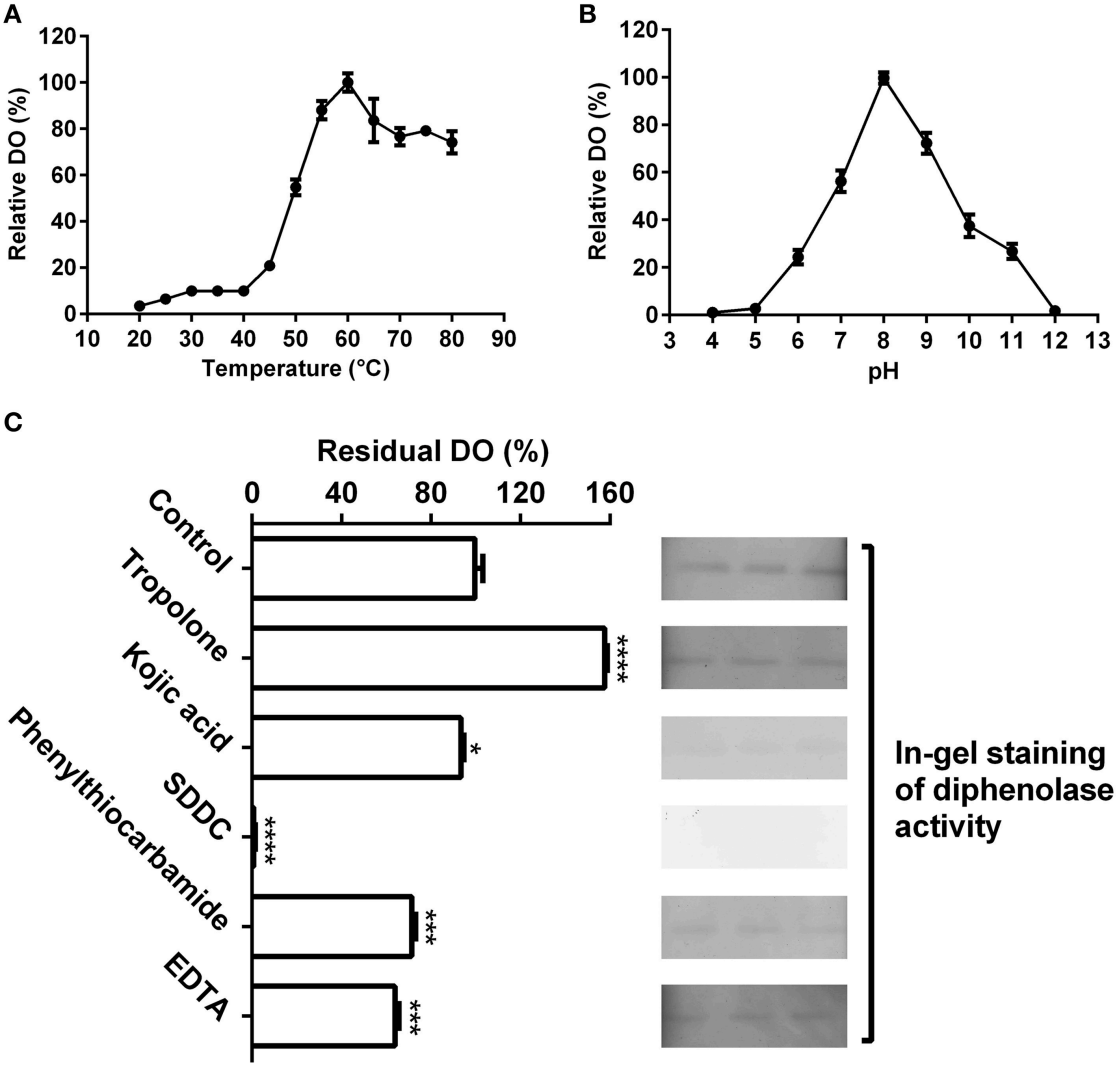
**Biochemical characterization of the diphenolase activity of the purified enzyme**. **(A)** Optimal temperature of the diphenolase activity (DO) of the purified enzyme toward L-DOPA. **(B)** Optimal pH of the DO toward L-DOPA. **(C)** Effects of inhibitors (final concentration, 1 mM) on the DO toward L-DOPA. Left: DO was measured by spectrophotometer in the standard reaction mixture with or without (CK) inhibitors; Right: DO was detected by in-gel staining. After electrophoresis, each three lanes of the native PAGE gel with the purified enzyme (40 μL on every lane) were sliced out of the gel and incubated in 5 mM L-DOPA containing or not (CK) inhibitors for 60 min at 45°C. SDDC, sodium diethyldithiocarbamate. Data are from three independent experiments (mean ± SD). Statistical significance was analyzed by Student's *t*-test with the control; ^*^P < 0.05; ^***^P < 0.001; ^****^P < 0.0001.

As shown in Figure 4A, the purified enzyme had low diphenolase activity below 40°C, and from 40 to 60°C, the diphenolase activity rapidly grew and arrived at peak at 60°C. From 60 to 80°C, the activity decreased gradually, but remained more than 75% even at 80°C.

The pH had an important influence on diphenolase activity of the purified enzyme (Figure 4B). Below pH 5.0 and at pH 12 or higher, almost no diphenolase activity was detected, whereas from pH 5.0 to 8.0, the activity augmented rapidly and came to the maximum at pH 8.0, then quickly decreased with the increment of pH (Figure 4B).

At the optimal temperature 60°C and pH 8.0, the purified enzyme had a *K*_*m*_ value of 0.6 mM toward L-DOPA, calculated from the Lineweaver-Burk graphs (Supplementary Data 2). In addition, the effect of five common tyrosinase inhibitors on diphenolase activity of the purified enzyme was also examined by in-gel staining and spectrophotometer. As on the monophenolase activity, all inhibitors (final concentration, 1 mM) except tropolone significantly inhibited the diphenolase activity (Figure 4C). SDDC demonstrated the strongest inhibitory effect and led to approximately 99% loss of the activity. It was followed by phenylthiocarbamide and kojic acid, which resulted in 25–30% decrease in the diphenolase activity. EDTA showed an inhibitory impact slightly higher than that of kojic acid, leading to about 35% of decline in the activity. In contrast, tropolone increased the diphenolase activity of purified enzyme by about 55% (Figure 4C).

Taken together, these biochemical data revealed that the purified enzyme had not only the monophenolase activity but also the diphenolase activity, *i.e*., the full activity of classic tyrosinase. The question raised was if the purified enzyme was a classic tyrosinase, why its mono- and diphenolase activities could not be inhibited by tropolone, a slow-binding inhibitor for classic tyrosinase? To address this question, we turned to its peptide sequences for some cues.

### Nano-LC-MS/MS analysis of purified enzyme

In order to get some information from the peptide sequences of the purified enzyme, we employed nano-liquid chromatography tandem mass spectrometry (nano-LC-MS/MS) analysis, due to lacking amino acid sequences of tyrosinases from betalain-containing plants and absence of genome information of *Amaranthus cruentus*. The nano-LC-MS/MS analysis gave five clear peptides: GPILLEDYHLIEK, APGVQTPVIVR, FSTVIHER, ENNFKEPGER, TFAYADTHR (Supplementary Figure S1 and Table S1). Surprisingly, a MASCOT database search targeted all five peptides to catalases, including those from two betalain-producing plants *Suaeda salsa* and *Mesembryanthemum crystallinum*, and the sequence coverage of the catalase from *Suaeda salsa* (gi|15617223) matched to with the highest score of 174 was 13% (Supplementary Figure S2A).

### Characterization of the catalase activity of purified enzyme

**Figure 5:**
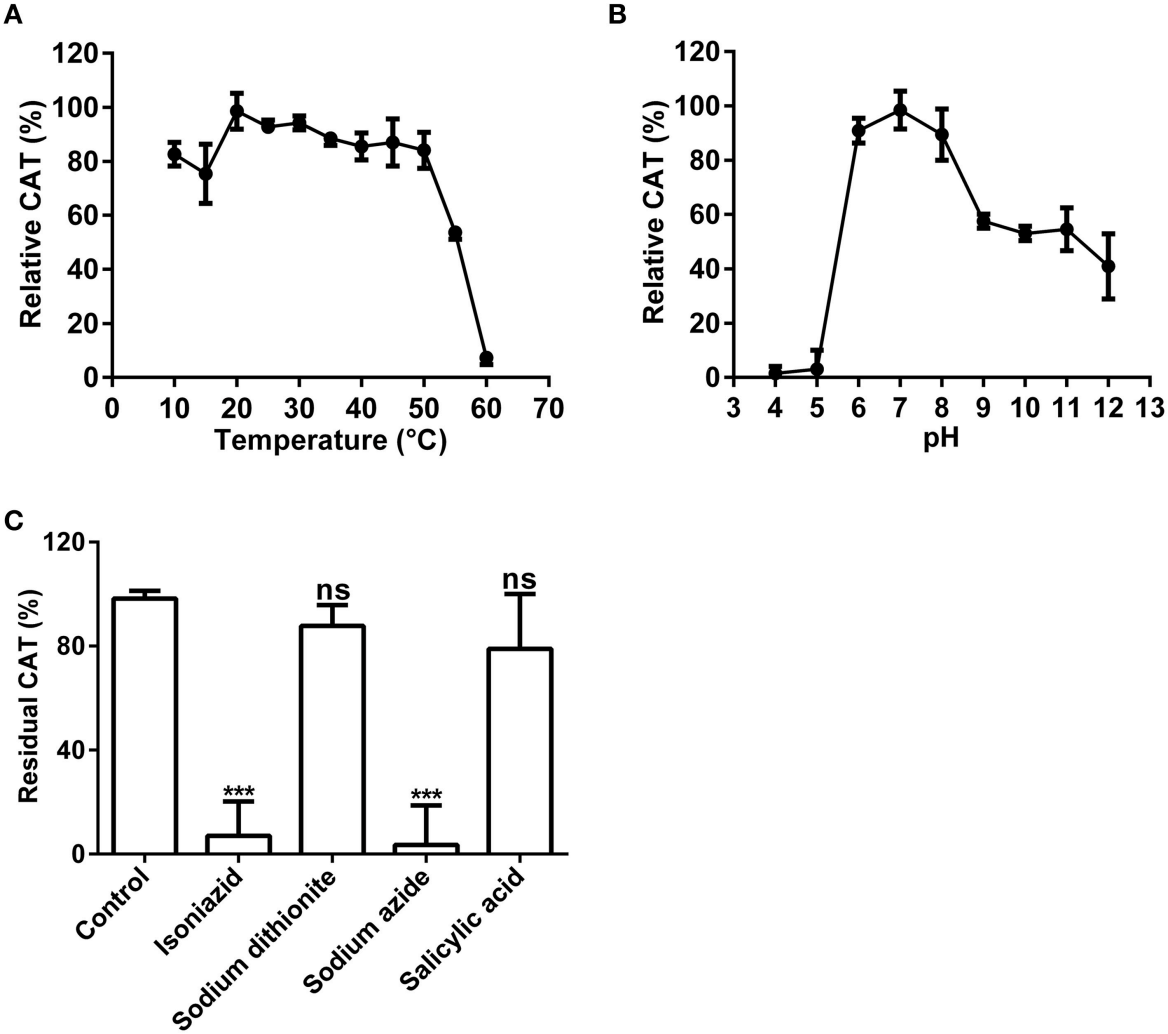
**Biochemical characterization of the catalase activity of the purified enzyme**. **(A)** Optimal temperature of the catalase activity (CAT) of the purified enzyme toward H_2_O_2_. **(B)** Optimal pH of the CAT toward H_2_O_2_. **(C)** Effects of inhibitors (final concentration, 1 mM) on the CAT toward H_2_O_2_. The residual CAT was measured by spectrophotometer in the standard reaction mixture with or without (CK) inhibitor (at 1 mM final concentration). Data are from three independent experiments (mean ± SD). Statistical significance was analyzed by Student's *t*-test with the control; ^***^P < 0.001; ns, not significant.

Above nano-LC-MS/MS and MASCOT analysis results revealed that the purified enzyme which possessed tyrosinase activity belonged to catalases biophysically. If this is true, biochemically, the purified enzyme should be able to catalyze degradation of H_2_O_2_ to produce oxygen and water as classic catalases (EC 1.11.1.6) (Aebi, 1984; Vetrano et al., 2005) in one hand, and to oxidize L-DOPA to form dopachrome (reddish in color) in other hand. To test these abilities, we performed *in vitro* catalysis of both hydrogen peroxide (H_2_O_2_) and L-DOPA in one reaction by the purified enzyme. Indeed, when the enzyme was added to the reaction mixture with both hydrogen peroxide (H_2_O_2_) and L-DOPA as substrates, the colorless reaction mixture became reddish in color and released bubbles (O_2_) (Supplementary Data 3). To further confirm the catalase-catalyzing ability, we conducted in-gel catalase activity assay (Weydert and Cullen, 2010) of the purified enzyme with bovine catalase as positive control. As expected, when the gel carrying the purified enzyme and bovine catalase in different lanes was incubated in the H_2_O_2_ solution and stained with ferric chloride/potassium ferricyanide, the achromatic bands appeared on the lanes with purified enzyme or bovine catalase, but not on the blank lane (Supplementary Figure S2B). Furthermore, at the optimal temperature 20°C (Figure 5A) and pH 7.0 (Figure 5B), the purified enzyme displayed a catalase activity with a *K*_*m*_ value of 188 mM, calculated from the Lineweaver-Burk graphs for the substrate H_2_O_2_ (Supplementary Data 4).

Above results showed that the purified enzyme possessed the catalase activity as classic catalases, in addition to its tyrosinase activity. Could this catalase activity be inhibited by common catalase inhibitors as classic catalases? To answer this question, we tested four common catalase inhibitors (isoniazid, salicylic acid, sodium azide, sodium dithionite) at the optimal temperature and pH. Inhibition experiments showed that almost all inhibitors tested could inhibit the catalase activity of the purified enzyme to some extent (Figure 5C). Sodium azide (at 1 mM) demonstrated the strongest inhibitory effect and led to approximately 95% loss of the catalase activity. It was followed by isoniazid (INH), a catalase-peroxidase specific inhibitor (Johnsson et al., 1997; Zámocký and Koller, 1999), which inhibited about 90% of the activity. For the rest, salicylic acid and sodium dithionite, their inhibitory effect was much lower and not significant, resulting in less than 25% decrease in catalase activity.

These results of catalysis and inhibition experiments demonstrated that the purified enzyme indeed possessed the biochemical characteristics of classic catalases, and in particular of catalase-peroxidases, in addition to that of tyrosinases.

### Nucleotide sequence, catalase active site and tyrosinase active site of purified enzyme

Both peptide fragment analysis and biochemical characterization revealed that the purified enzyme belonged to catalases but with tyrosinase activity. Accordingly, this implied that the purified enzyme would have both catalase active site and tyrosinase active site. To test this hypothesis, we cloned and analyzed the cDNA sequence of the purified enzyme. Based on the mRNA sequences of catalases from two betalain-producing plants, *Mesembryanthemum crystallinum* and *Suaeda maritima* subsp. *salsa* whose amino acid sequences exhibited high identity with the peptide fragments of the purified enzyme, we first designed the degenerate primer pairs (Table 1) to clone the core cDNA sequence of catalase from red amaranth leaves, then used RACE techniques to clone full-length cDNA sequence. We obtained a cDNA sequence of 1951 bp (GenBank accession number: KP710221) that consisted of 137 bp/335 bp of 5′/3′-UTR and a complete CDS which encoded a peptide of 492 amino acids deductively. Based on the cDNA, an entire CDS coding for 492 amino acids was cloned and identical to that of RACE cDNA. The predicted molecular weight of the deduced protein was 57 kDa, which was in agreement with that determined by SDS-PAGE.

**Figure 6:**
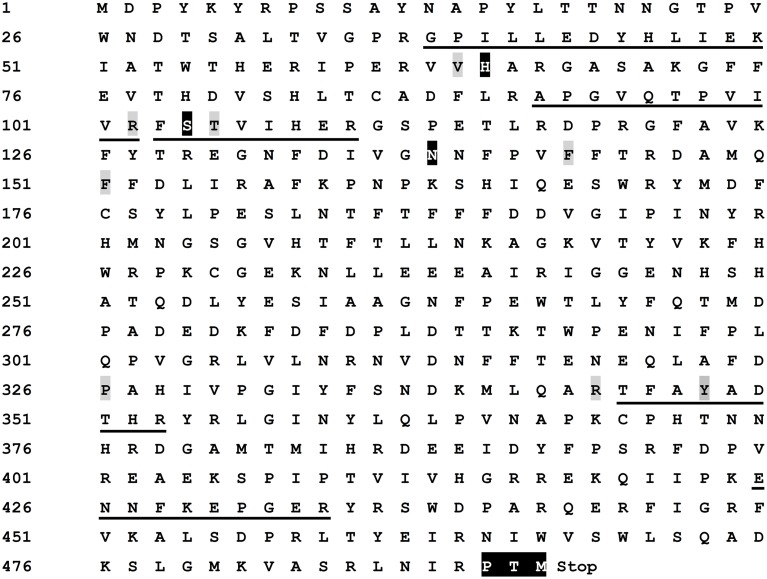
**Characterization of deduced amino acid sequence of cloned cDNA**. The amino acid sequences identical to the peptide fragments determined by nano-LC-MS/MS were underlined. The conserved catalase catalytic amino acid residues (active site), 65-H (His), 104-S (Ser), and 138-N (Asn), were white and black-marked. The distal heme binding domain [64-V (Val), 102-R (Arg), 105-T (Thr), 143-F (Phe), and 151-F] and proximal heme binding domain [326-P (Pro), 344-R, and 348-Y (Tyr)] were gray-marked. A putative conserved internal peroxisomal targeting signal (PTM) was in white and black at the C-terminal [490-P, 491-T, and 492-M (Met)]. The stop signal was represented by “Stop”. The sequence has been deposited in GenBank as Accession No. KP710221.

The deduced amino acid sequence had an identity of 88.92% to those of catalases from *Beta vulgaris* subsp. *maritima, M. crystallinum*, and *S. maritima* subsp. *salsa* (Supplementary Figure S3), and as expected, it contained all five peptide sequences determined by nano-LC-MS/MS analysis (Figure 6). As shown in Figure 6, in the deduced amino acid sequence, there presented conserved catalytic amino acid residues (active site) of common catalases (Kwon and An, 2001), 65-H, 104-S, and 138-N (white and black-marked), distal heme binding domain (64-V, 102-R, 105-T, 143-F, and 151-F) and proximal heme binding domain (326-P, 344-R, and 348-Y). In addition, a putative conserved internal peroxisomal targeting signal (PTM) was detected at the C-terminal at the position 490–492. These features showed that the protein encoded by cloned cDNA was a catalase including nano-LC-MS/MS-determined peptide sequences of the purified enzyme, signifying that the purified enzyme was encoded by this cDNA.

Having identified the catalase active site, we turned our attention to search potential oxidase site(s) in the deduced amino acid sequences of cloned cDNA. Therefore, we compared our amino acid sequences with those of some mammalian catalases that were reported to possess oxidase activity in the absence of hydrogen peroxide or any added cofactors (Vetrano et al., 2005). The result revealed that the potential conserved phenol oxidase active site, composed of the amino acid residuals 74-R, 111-R, 131-F, and 364-R in both bovine liver and human catalases (Putnam et al., 2000; Chelikani et al., 2004; Vetrano et al., 2005), was also present in the deduced amino acid sequences of cloned cDNA, and the interspace (number of amino acid) between the composing amino acid residuals was identical among bovine liver catalase, human catalase and our cDNA-encoded catalase (see Supplementary Data 5). However, we didn't find any characteristic copper-binding site of classic tyrosinases or ordinary PPOs (Supplementary Figure S4).

### Phylogenetic analysis and related statistical analysis

**Figure 7:**
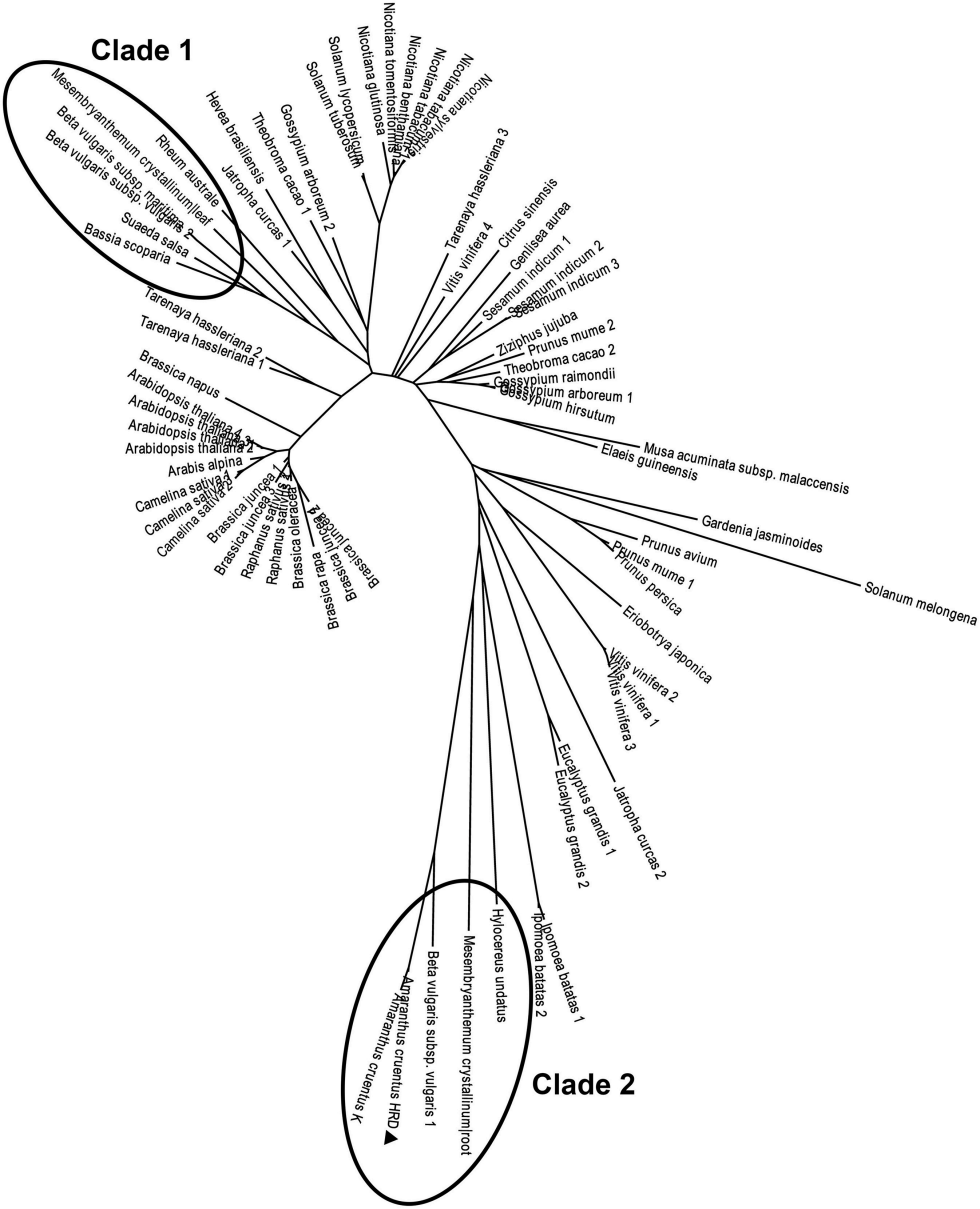
**Phylogenetic analysis of AcCATPO and other plant catalases**. Sequences were retrieved from a basic local alignment search (BLAST) (National Center for Biotechnology Information, NCBI). The AcCATPO (marked by triangle) sequence obtained was used as a query selecting results with total score >800 (*E* = 0, except sectorial sequences). Accession codes (GenBank) for catalase sequences from betalain-producing plants (marked by ellipses, Clades 1 and 2) are: *Beta vulgaris* subsp. *vulgaris* 1,2 (gi:731361773, 731361778), *Beta vulgaris* subsp. *Maritime* (gi:564116010), *Mesembryanthemum crystallinum* root, leaf (gi:3202034, 3202032), *Rheum australe* (gi:197312885), *Hylocereus undatus* (gi:571032250), *Bassia scoparia* (gi:686477557), and *Suaeda salsa* (gi:20138726). *Amaranthus cruentus* K: AA of mira_lrc70 CDS from the *Amaranthus cruentus* cv. Kerala transcriptome (Hatlestad et al., 2012). Accession codes for the non-betalain-producing plants: *Prunus mume* 1,2 (gi:645265094, 645265092), *Gardenia jasminoides* (gi:721750661), *Nicotiana tabacum* 1,2 (gi:429535123, 2459684), *Prunus persica* (gi:32526568), *Nicotiana benthamiana* (gi:219560127), *Vitis vinifera* 1,2,3,4 (gi:526117723, 359476986, 819330654, 731427403), *Nicotiana sylvestris* (gi:698496386), *Prunus avium* (gi:121078773), *Nicotiana tomentosiformis* (gi:697176653), *Solanum tuberosum* (gi:565347640), *Eucalyptus grandis* 1,2 (gi:702368481, 702368496), *Solanum lycopersicum* (gi:854282574), *Tarenaya hassleriana* 1,2,3 (gi:729311669, 729453171, 729330417), *Musa acuminata* subsp. *malaccensis* (gi:695050168), *Theobroma cacao* 1,2 (gi:590701975, 590595360), *Sesamum indicum* 1,2,3 (gi:749385032, 747055694, 747055696), *Brassica juncea* 1,2,3,4 (gi:4336754, 4336756, 4336758, 4336752), *Camelina sativa* 1,2,3 (gi:727512115, 727548051, 727523245), *Ipomoea batatas* 1,2 (gi:282935438, 115703), *Raphanus sativus* 1,2 (gi:8050693, 7302765), *Ziziphus jujuba* (gi:357966938), *Brassica rapa* (gi:685256782), *Jatropha curcas* 1,2 (gi:802555337, 806776603), *Nicotiana glutinosa* (gi:2253291), *Arabis alpina* (gi:674237382), *Arabidopsis thaliana* 1,2,3,4 (gi:15236264, 1246399, 15451166, 444340), *Brassica oleracea* (gi:259122789), *Brassica napus* (gi:169244543), *Solanum melongena* (gi:562787), *Genlisea aurea* (gi:527202914), *Gossypium arboretum* 1,2 (gi:728845709, 728833533), *Gossypium hirsutum* (gi:211906480), *Eriobotrya japonica* (gi:442736195), *Gossypium raimondii* (gi:823132870), *Hevea brasiliensis* (gi:315937176), *Elaeis guineensis* (gi:743775712), and *Citrus sinensis* (gi:568839653).

There arose the question whether those characteristics of the purified enzyme (named AcCATPO) were typical only in catalases from betalain-producing plants or prevalent in non-betalain plant catalases. To answer this question, we performed bioinformatics analysis of relevant data in GenBank and *Amaranthus cruentus* cv. Kerala transcriptome data (Hatlestad et al., 2012) with our AcCATPO amino acid sequence as query. The results showed that both conserved catalase active site and potential oxidase active site were present in the catalases of both betalain plants and non-betalain plants, and obvious difference between two group plants lay in the proportion of triple amino acids (PTM) used as peroxisomal targeting signal: 90% in the catalases of betalain plants and 16.7% in those of non-betalain plants (Table 3 and Supplementary Data 5). In addition, plant catalases showed high amino acid identity among themselves and even *ca*. 82.6% identity with those of bovine liver and human catalases (Supplementary Data 5). Based on the identity, we constructed a phylogenetic tree for plant catalases with complete cDNA sequences (Figure 7). As showed in Figure 7, the catalases of betalain plants were divided into two distinct clades, and our AcCATPO was in Clade 2, adjacent to *M. crystallinum* root catalase, sugar beet catalase 1 and *A. cruentus* cv. Kerala catalase.

**Table 3:** **Comparative analysis of the active sites and targeting signal of plant catalases**.

	**Total**	**Containing catalase active sites**		**Containing oxidase active sites**		**Containing peroxisomal targeting signal (PTM)**	
		**Number**	**Proportion (%)**	**Number**	**Proportion (%)**	**Number**	**Proportion**
From betalain-forming plants	10	10	100	10	100	9	90
Other plants	60	59	98.3	60	100	10	16.7

*The AcCATPO sequence obtained was used as a query selecting results with total score >800 (E = 0, except partial sequences)*.

### AcCATPO and betalain biosynthesis

**Figure 8:**
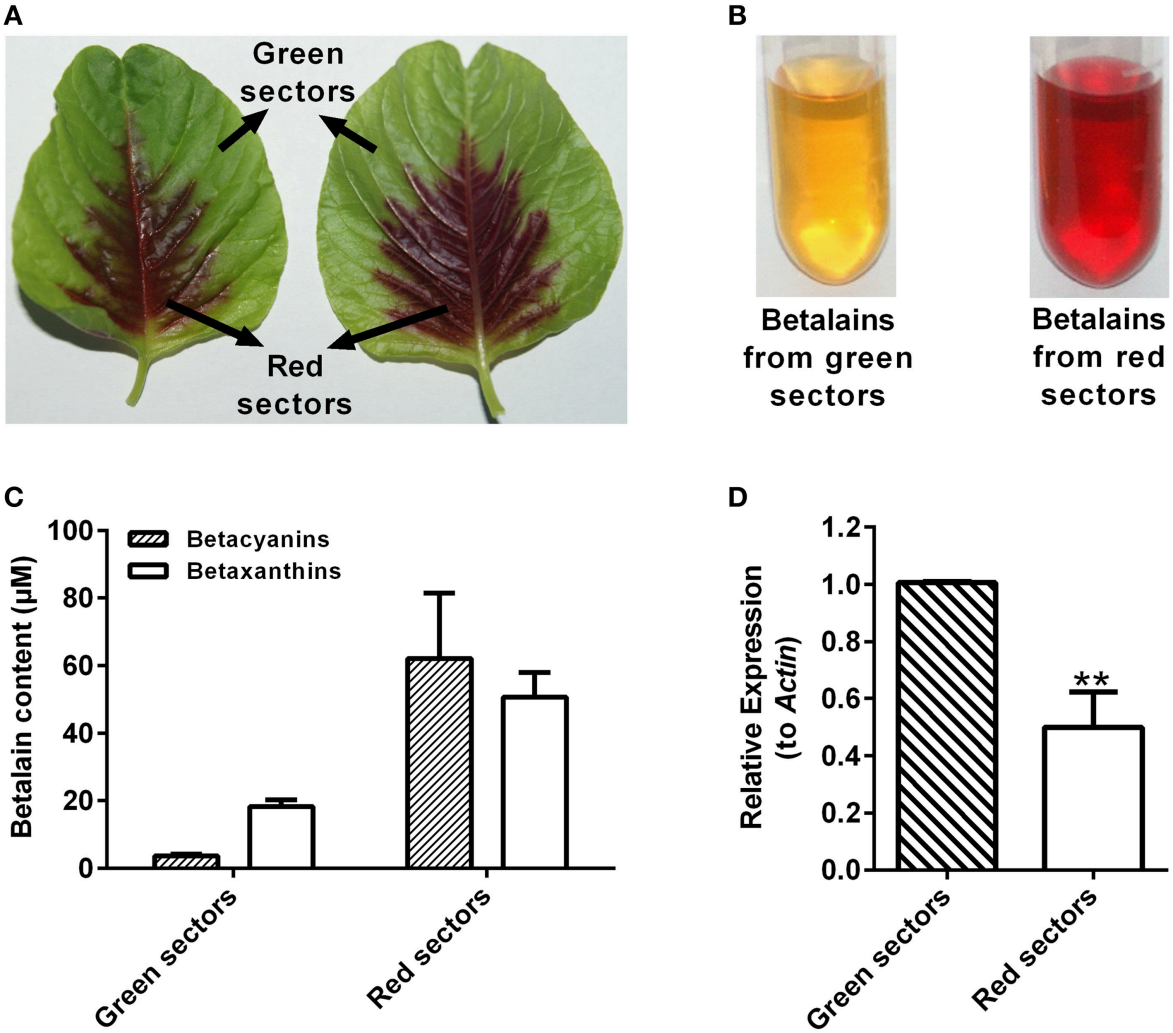
**Betalain pigment analysis and expression levels of ***AcCATPO*** in different colored leaf sectors of ***Amaranthus tricolor*****. **(A)** Images depicting different colored leaf sectors of *A. tricolor*. **(B)** Images depicting the betalain pigments from green sectors and red sectors. **(C)** Betalain pigment analysis of green sectors and red sectors. **(D)** Expression levels of *AcCATPO* in green sectors and red sectors analyzed by qRT-PCR. These levels were depicted as expression ratios relative to *ACT*. Three independent experiments were performed, each with three technical replicates. Values are means ± SD (*n* = 3) from one representative experiment. Asterisk indicates significant difference relative to expression levels of *AcCATPO* in green sectors (Student's *t*-test, ^**^P < 0.01).

The *AcCATPO* possessed the monophenolase activity toward L-tyrosine and diphenolase activity to L-DOPA besides the catalase activity, so was it involved in betalain biosynthesis? With this question in mind, we examined the relation between the betalain content and the expression level of *AcCATPO* in green and red leaf sectors of *A. tricolor*. As shown in Figures 8A–C, the major betalain in green sectors was yellowish betaxanthins (the ratio of betaxanthins/betacyanins = 4.9), whereas in red sectors, the violet betacyanins were visibly higher than betaxanthins (the ratio of betaxanthins/betacyanins = 0.8) although both pigments were abundant. The content of total betalains in red sectors was higher than that in green sectors (approximately 16.6-fold in betacyanins and 2.8-fold in betaxanthins). The *AcCATPO* transcript abundance revealed by qRT-PCR was about twice higher in green sectors than that in red ones, and it was positively correlated with the ratio of betaxanthins to betacyanins (Figure 8D). This correlation suggested that the *AcCATPO* might be involved in betaxanthins biosynthesis, if it played a role in betalain biosynthesis.

Collectively, these data suggested that the purified enzyme was a catalase-phenol oxidase (CATPO), the fourth group catalase which was verified by cloned cDNA. This *CATPO* might be involved in the biosynthesis of betaxanthins.

## Discussion

Tyrosinase, a particular type of PPO, has long been proposed to be involved in plant betalain biosynthesis, catalyzing hydroxylation of L-tyrosine to form L-DOPA (EC 1.14.18.1, monophenolase activity) and subsequent oxidation of L-DOPA to produce dopaquinone (EC 1.10.3.1, diphenolase activity) (Figure 1; see Strack et al., 2003; Han et al., 2009; Gandía-Herrero and García-Carmona, 2013, and references therein). In the search of such a tyrosinase, we purified a homotrimeric enzyme of *ca*. 170 kDa to apparent homogeneity by gel slice with L-DOPA as substrate from red-violet leaves of red amaranth, a betacyanin-producing C_4_ plant (Figure 2). As expected, the purified enzyme displayed both diphenolase activity toward L-DOPA and monophenolase activity toward L-tyrosine in the presence of the cofactor (Figures 3, 4) as pointed by Gowda and Paul (2002) *in vitro* catalysis assay. However, in inhibition experiments, both monophenolase and diphenolase activities of the purified enzyme were enhanced instead of being inhibited by tropolone (Figures 3F, 4C), a slow-binding inhibitor for classic tyrosinases (Espín and Wichers, 1999; Chang, 2009). To address this contradiction, we analyzed peptide sequences of the purified enzyme through nano-LC-MS/MS analysis and obtained five clear peptide fragments (Supplementary Figure S1 and Table S1). MASCOT database search surprisingly targeted all obtained peptide fragments to catalases, including those from betalain-producing plants *M. crystallinum and S. salsa* (Supplementary Figure S2A). This might explain why tropolone didn't inhibit the tyrosinase activity of the purified enzyme, but this phenomenon also raised a new question whether the purified enzyme could function as common catalases in addition to its tyrosinase activity. Our results showed that the purified enzyme indeed catalyzed degradation of H_2_O_2_ to produce oxygen (bubbles released in the reaction mixture) and water (Supplementary Data 3) as classic catalases, and oxidized L-DOPA to form reddish dopachrome as classic tyrosinases. This catalyzing ability was further confirmed by in-gel catalase activity assay with bovine catalase as positive control (Supplementary Figure S2B) and by inhibiting assays with classic catalase-specific inhibitors (Figure 5C). In addition, the specific activities of the monophenolase, diphenolase and catalase of the purified enzyme increased almost equally (7.7-, 7.6-, and 7.2-fold, respectively, Table 2), which indicates that the three activities are from the same protein. These biochemical data suggested that the purified enzyme from red–violet leaves of red amaranth was a catalase-phenol oxidase (named “AcCATPO”), the fourth group catalase. Its kinetic properties were summarized in Table 4.

**Table 4:** **Kinetic properties of the purified enzyme, AcCATPO**.

**AcCATPO**	**Optimal T (°C)**	**Optimal pH**	***K_*m*_*value (mM)**	***V_*max*_* (μM product/min)**	**[E_T_] (μM)**	***K_*cat*_* (S^−1^)**	***K_*cat*_*/*K_*m*_* (M^−1^ S^−1^)**
MO	60	8.0	0.2	0.079	0.0345	0.04	200
DO	60	8.0	0.6	0.75	0.0345	0.36	600
CAT	20	7.0	188	5942	0.0345	2871	15271

*MO, monophenolase activity to L-tyrosine; DO, diphenolase activity toward L-DOPA; CAT, catalase activity toward H_2_O_2_; T, temperature*.

As purified AcCATPO functioned as classic catalases and tyrosinases at the same time, it may have both catalase active site and tyrosinase active site, logically. Indeed, in the deductive peptide encoded by cloned cDNA (deposited in GenBank as KP710221), we identified both catalase active site and possible phenol oxidase active site (Figure 6 and Supplementary Data 5) as expected. Thus the catalase-phenol oxidase functions of AcCATPO purified from red amaranth were supported by the cDNA sequence. It is worth to note that we didn't identify any copper-binding sites, the characteristic active sites of classic tyrosinases or PPOs (Supplementary Figure S4). This may explain why both monophenolase and diphenolase activities of purified AcCATPO were not inhibited by tropolone, one of common inhibitors of classic tyrosinases or PPOs. Then, why both activities were heavily inhibited by the Cu^2+^ chelator, SDDC (Figures 3F, 4C)? Kleschyov et al. (2000) and Pieper et al. (2003) demonstrated that the SDDC could also powerfully chelate iron. This iron-chelating may make the iron unavailable to AcCATPO and thus inhibit AcCATPO's activities, because the phenolic compound oxidation was mediated by formation of an oxyferryl intermediate in the oxidase active site of CATPO (Vetrano et al., 2005).

It is well known that classic catalases were classified into three groups: monofunctional heme catalases, catalase-peroxidases and manganese catalases (Zámocký and Koller, 1999; Switala and Loewen, 2002). Recently, the fourth group, catalase-phenol oxidases (CATPOs), was introduced, because its dual catalase-phenol oxidase activity was detected not only in fungi catalases such as those of *Scytalidium thermophilum* and *Aspergillus niger*, but also in mammalian catalases such as human catalase and bovine liver catalase (Vetrano et al., 2005; Kocabas et al., 2008). In plant, such CATPO has not been reported, to our knowledge. Our purified AcCATPO possesses dual catalase-phenol oxidase activity (Table 2) in one hand, and its amino acid sequence contains both conserved catalase active site and potential phenol oxidase active site (Figure 6 and Supplementary Data 5) in other hand. These results show that the new group of catalase, CATPO, is also present at least in a betalain-producing plant, red amaranth. In order to know whether this new group of catalases exists in non-betalain plants, we compared the amino acid sequences of our AcCATPO and CATPOs known with those of plant catalases collected from GenBank, and found that the potential oxidase active site, besides the catalase active site, was prevalent even in the catalases of non-betalain plants (Table 3 and Supplementary Data 5). This suggests that the 4^th^ group of catalases, CATPOs, is present in non-betalain plants, too, although biochemical evidence is needed.

The CATPOs were reported to be capable of *o*-diphenolic oxidation in the absence of H_2_O_2_ (diphenolase activity), but not monophenolic oxidation (monophenolase activity) (Kocabas et al., 2008). However, our purified AcCATPO did display the monophenolase activity toward L-tyrosine, in addition to CATPO activity (Figure 3). This difference may be attributed to the approach used to detect the monophenolase activity. It was reported that monophenolase activity of tyrosinase presented a characteristic lag period, and sufficient amount of diphenols was needed as the cofactor to diminish or abolish this lag period (Pomerant and Warner, 1967; Duckworth and Coleman, 1970; Chang, 2009). Thus, we selected and used 0.057 mM L-DOPA as the cofactor and successfully detected the monophenolase activity toward L-tyrosine in both purified AcCATPO and commercial mushroom tyrosinase (Figure 3C).

We noted that the monophenolase activity and diphenolase activity of purified AcCATPO were not equal in the strength (Table 2), and this difference also existed in and among tyrosinases from animals, some higher fungi and betalain-forming plants/callus cultures (Supplementary Table S2). We observed that our purified enzyme had a similar affinity to those substrates as human and murine tyrosinases.

In betalain-producing plants and fungi, tyrosinase has been presumed as the enzyme to catalyze the first two steps of betalain biosynthesis: hydroxylation of L-tyrosine to L-DOPA through its monophenolase activity and subsequent conversion of resulting L-DOPA to dopaquinone which can become *cyclo*-DOPA spontaneously through its diphenolase activity (see Strack et al., 2003; Han et al., 2009; Gandía-Herrero and García-Carmona, 2013, for details). However, this kind of PPO-type tyrosinase has not been approved experimentally, in particular *in vivo*. In contrast, Hatlestad and his colleagues recently discovered that CYP76AD1, a new cytochrome P450 from sugar beet could oxidize L-DOPA to form *cyclo*-DOPA and thus could complement the *R* mutant to produce red pigments (Hatlestad et al., 2012). Our AcCATPO purified from red amaranth possessed not only the catalase activity, but also the full enzymatic activity of proposed tyrosinases, *i.e*., the monophenolase activity toward L-tyrosine and the diphenolase activity toward L-DOPA, thus, logically, it should have a role to play in plant betalain biosynthesis, meaning that the catalase-phenol oxidase might be involved in betalain biosynthesis. To test this inference preliminarily, we examined the correlation between *AcCATPO* expression level and betalain contents in green sectors and red sectors of leaves of common amaranth (*A. tricolor*) (Figure 8). We observed a positive correlation between the expression level of *AcCATPO* and the ratio of betaxanthins to betacyanins, suggesting that the *AcCATPO* might be mainly involved in the biosynthesis of betaxanthins, but not that of betacyanins which needs a new P450 for *cyclo*-DOPA formation from L-DOPA (Hatlestad et al., 2012). If this is true, the AcCATPO would be located in cytoplasm because of vacuole-location (Strack et al., 2003; Hatlestad et al., 2012) and poor mobility (Figure 8A) of the betalains in one hand, and predicted cytoplasm location of DODA1 (UniProtKB-Q7XA48), the key enzyme for synthesizing betalamic acid, the chromophore of betalains in other hand. Bearing this implication in mind, we re-checked the amino acid sequence of AcCATPO for searching possible cytoplasm targeting signals, and indeed, we identified a putative conserved internal peroxisomal targeting signal, “PTM”, at the C-terminal of the sequence (Figure 6) and found, by bioinformatics analysis, that this peroxisomal targeting signal was present in the catalases of 90% betalain plants (Table 3). Of course, we are clearly aware that the exact subcellular location of *AcCATPO* and its involvement in betalain biosynthesis needs to be approved by the evidence of functional analysis of *AcCATPO* gene, which is under the way.

In summary, we identified, purified and characterized a catalase-phenol oxidase, AcCATPO, from red amaranth, a betalain-producing plant, and this Group 4 catalase had been only reported present in fungi and mammals including human (Vetrano et al., 2005; Kocabas et al., 2008). Successful purification and characterization of such a catalase-phenol oxidase in plant opens a window to see the function of this new class of catalases in plants, and in particular its role in betalain biosynthesis in betalain-producing plants.

## Author contributions

X-LT and X-GX planned the studies and prepared the manuscript. X-LT and X-GX designed experiments. X-LT and NC performed the experiments. All authors reviewed the results and approved the final version of the manuscript.

## Funding

This work was supported by grants from National Natural Science Foundation of China (Grant No. 31070268) and Chinese High-Tech R&D Program “863” (Grant No. 2008AA10Z103) to X-GX.

### Conflict of interest statement

The authors declare that the research was conducted in the absence of any commercial or financial relationships that could be construed as a potential conflict of interest. The reviewer Mingyue Gou and handling Editor Chang-Jun Liu declared their shared affiliation, and the handling Editor states that, nevertheless, the process met the standards of a fair and objective review.

## Abbreviations

PPO: polyphenol oxidase
nano-LC-MS/MS: nano-liquid chromatography tandem mass spectrometry
RACE: rapid amplification of cDNA
RT-PCR: reverse transcription polymerase chain reaction
CATPO: catalase-phenol oxidase.

## References

[B1] Aebi H. (1984). Catalase *in vitro*. Meth. Enzymol. 105, 121–126. 10.1016/S0076-6879(84)05016-3 6727660

[B2] Beck G. Cardinale S. Wang L. Reiner M. Sugumaran M. (1996). Characterization of a defense complex consisting of interleukin 1 and phenol oxidase from the hemolymph of the tobacco hornworm, *Manduca sexta*. J. Biol. Chem. 271, 11035–11038. 10.1074/jbc.271.19.11035 8626641

[B3] Bollag D. M. Edelstein S. J. (1991). Protein Methods. New York, NY: Wiley-Liss, Inc, 85–87.

[B4] Bradford M. M. (1976). A rapid and sensitive method for the quantitation of microgram quantities of protein utilizing the principle of protein-dye binding. Anal. Biochem. 72, 248–254. 10.1016/0003-2697(76)90527-3 942051

[B5] Castellanos-Santiago E. Yahia E. A. (2008). Identification and quantification of betalains from the fruits of 10 Mexican prickly pear cultivars by high-performance liquid chromatography and electrospray ionization mass spectrometry. J. Agric. Food Chem. 56, 5758–5764. 10.1021/jf800362t 18578538

[B6] Chang T. S. (2009). An updated review of tyrosinase inhibitors. Int. J. Mol. Sci. 10, 2440–2475. 10.3390/ijms10062440 19582213 PMC2705500

[B7] Chelikani P. Fita I. Loewen P. C. (2004). Diversity of structures and properties among catalases. Cell. Mol. Life Sci. 61, 192–208. 10.1007/s00018-003-3206-5 14745498 PMC11138816

[B8] Cuénoud P. Savolainen V. Chatrou L. W. Powell M. Grayer R. J. Chase M. W. (2002). Molecular phylogenetics of Caryophyllales based on nuclear 18S rDNA and plastid *rbcL, atpB*, and *matK* DNA sequences. Am. J. Bot. 89, 132–144. 10.3732/ajb.89.1.132 21669721

[B9] Duckworth H. W. Coleman J. E. (1970). Physicochemical and kinetic properties of mushroom tyrosinase. J. Biol. Chem. 245, 1613–1625. 4985615

[B10] Espín J. C. Wichers H. J. (1999). Slow-binding inhibition of mushroom (*Agaricus bisporus*) tyrosinase isoforms by tropolone. J. Agric. Food Chem. 47, 2638–2644. 10.1021/jf981055b 10552538

[B11] Espín J. C. Wichers H. J. (2001). Effect of captopril on mushroom tyrosinase activity *in vitro*. Biochim. Biophys. Acta 1544, 289–300. 10.1016/S0167-4838(00)00230-2 11341938

[B12] Gandía-Herrero F. Escribano J. García-Carmona F. (2005). Betaxanthins as substrates for tyrosinase. an approach to the role of tyrosinase in the biosynthetic pathway of betalains. Plant Physiol. 138, 421–432. 10.1104/pp.104.057992 15805475 PMC1104195

[B13] Gandía-Herrero F. Escribano J. García-Carmona F. (2014). Biological activities of plant pigments betalains. Crit. Rev. Food Sci. Nutr. 10.1080/10408398.2012.740103. [Epub ahead of print]. 25118005

[B14] Gandía-Herrero F. García-Carmona F. (2013). Biosynthesis of betalains: yellow and violet plant pigments. Trends Plant Sci. 18, 334–343. 10.1016/j.tplants.2013.01.003 23395307

[B15] Gandía-Herrero F. García-Carmona F. Escribano J. (2004). Purification and characterization of a latent polyphenol oxidase from beet root (*Beta vulgaris* L.). J. Agric. Food Chem. 52, 609–615. 10.1021/jf034381m 14759157

[B16] Gandía-Herrero F. Jimenez-Atienzar M. Cabanes J. Escribano J. García-Carmona F. (2009). Fluorescence detection of tyrosinase activity on dopamine-betaxanthin purified from *Portulaca oleracea* (common purslane) flowers. J. Agric. Food Chem. 57, 2523–2528. 10.1021/jf803608x 19227976

[B17] Gao Z. J. Han X. H. Xiao X. G. (2009). Purification and characterization of polyphenol oxidase from red Swiss chard (*Beta vulgaris* subspecies *cicla*) leaves. Food Chem. 117, 342–348. 10.1016/j.foodchem.2009.04.013

[B18] Gowda L. R. Paul B. (2002). Diphenol activation of the monophenolase and diphenolase activities of field bean (*Dolichos lablab*) polyphenol oxidase. J. Agric. Food Chem. 50, 1608–1614. 10.1021/jf010913s 11879044

[B19] Grotewold E. (2006). The genetics and biochemistry of floral pigments. Annu. Rev. Plant Biol. 57, 761–780. 10.1146/annurev.arplant.57.032905.105248 16669781

[B20] Han X. H. Gao Z. J. Xiao X. G. (2009). Enzymes and genes involved in the betalain biosynthesis in higher plants. Afr. J. Biotechnol. 8, 6735–6744.

[B21] Harris N. N. Javellana J. Davies K. M. Lewis D. H. Jameson P. E. Deroles S. C.. (2012). Betalain production is possible in anthocyanin-producing plant species given the presence of DOPA-dioxygenase and L-DOPA. BMC Plant Biol. 12:34. 10.1186/1471-2229-12-34 22409631 PMC3317834

[B22] Hatlestad G. J. Sunnadeniya R. M. Akhavan N. A. Gonzalez A. Goldman I. L. McGrath J. M.. (2012). The beet R locus encodes a new cytochrome P450 required for red betalain production. Nat. Genet. 44, 816–820. 10.1038/ng.2297 22660548

[B23] Johnsson K. Froland W. A. Schultz P. G. (1997). Overexpression, purification, and characterization of the catalase-peroxidase KatG from *Mycobacterium tuberculosis*. J. Biol. Chem. 272, 2834–2840. 10.1074/jbc.272.5.2834 9006925

[B24] Kanner J. Harel S. Granit R. (2001). Betalains - a new class of dietary cationized antioxidants. J. Agric. Food Chem. 49, 5178–5185. 10.1021/jf010456f 11714300

[B25] Kleschyov A. L. Mollnau H. Oelze M. Meinertz T. Huang Y. Harrison D. G.. (2000). Spin trapping of vascular nitric oxide using colloid Fe(II)-diethyldithiocarbamate. Biochem. Biophys. Res. Commun. 275, 672–677. 10.1006/bbrc.2000.3361 10964721

[B26] Kocabas D. S. Bakir U. Phillips S. E. V. McPherson M. J. Ogel Z. B. (2008). Purification, characterization, and identification of a novel bifunctional catalase-phenol oxidase from *Scytalidium thermophilum*. Appl. Microbiol. Biotechnol. 79, 407–415. 10.1007/s00253-008-1437-y 18369615

[B27] Kwon S. I. An C. S. (2001). Molecular cloning, characterization and expression analysis of a catalase cDNA from hot pepper (*Capsicum annuum* L.). Plant Sci. 160, 961–969. 10.1016/S0168-9452(01)00332-6 11297793

[B28] Laemmli U. K. (1970). Cleavage of structural proteins during the assembly of the head of bacteriophage T4. Nature 227, 680–685. 10.1038/227680a0 5432063

[B29] McMahon A. M. Doyle E. M. Brooks S. O'Connor K. E. (2007). Biochemical characterisation of the coexisting tyrosinase and laccase in the soil bacterium *Pseudomonas putida* F6. Enzyme Microb. Technol. 40, 1435–1441. 10.1016/j.enzmictec.2006.10.020

[B30] Merle P.-L. Sabourault C. Richier S. Allemand D. Furla P. (2007). Catalase characterization and implication in bleaching of a symbiotic sea anemone. Free Radic. Biol. Med. 42, 236–246. 10.1016/j.freeradbiomed.2006.10.038 17189829

[B31] Mueller L. A. Hinz U. Zryd J. P. (1996). Characterization of a tyrosinase from *Amanita muscaria* involved in betalain biosynthesis. Phytochemistry 42, 1511–1515. 10.1016/0031-9422(96)00171-9

[B32] Musso H. (1979). The pigments of fly agaric, *Amanita muscaria*. Tetrahedron 35, 2843–2853. 10.1016/S0040-4020(01)99498-0

[B33] Nellaiappan K. Vinayagam A. (1986). A rapid method for detection of tyrosinase activity in electrophoresis. Stain Technol. 61, 269–272. 10.3109/10520298609109952 3097880

[B34] Pieper G. M. Nilakantan V. Hilton G. Halligan N. L. Felix C. C. Kampalath B.. (2003). Mechanisms of the protective action of diethyldithiocarbamate-iron complex on acute cardiac allograft rejection. Am. J. Physiol. Heart Circ. Physiol. 284, H1542–H1551. 10.1152/ajpheart.00913.2002 12679325

[B35] Pomerant S. H. Warner M. C. (1967). 3,4-Dihydroxy-L-phenylalanine as the tyrosinase cofactor. J. Biol. Chem. 242, 5308–5314. 4965136

[B36] Putnam C. D. Arvai A. S. Bourne Y. Tainer J. A. (2000). Active and inhibited human catalase structures: ligand and NADPH binding and catalytic mechanism. J. Mol. Biol. 296, 295–309. 10.1006/jmbi.1999.3458 10656833

[B37] Rodríguez-López J. N. Tudela J. Varón R. García-carmona F. García-cánovas F. (1992). Analysis of a kinetic model for melanin biosynthesis pathway. J. Biol. Chem. 267, 3801–3810. 1740428

[B38] Ros J. Rodríguez-López J. García-Cánovas F. (1994). Tyrosinase: kinetic analysis of the transient phase and the steady state. Biochim. Biophys. Acta 1204, 33–42. 10.1016/0167-4838(94)90029-9 8305473

[B39] Sánchez-Ferrer Á. Rodríguez-López J. N. García-Cánovas F. García-Carmona F. (1995). Tyrosinase: a comprehensive review of its mechanism. Biochim. Biophys. Acta 1247, 1–11. 10.1016/0167-4838(94)00204-T 7873577

[B40] Selinheimo E. NiEidhin D. Steffensen C. Nielsen J. Lomascolo A. Halaouli S.. (2007). Comparison of the characteristics of fungal and plant tyrosinases. J. Biotechnol. 130, 471–480. 10.1016/j.jbiotec.2007.05.018 17602775

[B41] Steiner U. Schliemann W. Bohm H. Strack D. (1999). Tyrosinase involved in betalain biosynthesis of higher plants. Planta 208, 114–124. 10.1007/s004250050541

[B42] Stintzing F. C. Carle R. (2004). Functional properties of anthocyanins and betalains in plants, food, and in human nutrition. Trends Food Sci. Technol. 15, 19–38. 10.1016/j.tifs.2003.07.004

[B43] Strack D. Schliemann W. (2001). Bifunctional polyphenol oxidases: novel functions in plant pigment biosynthesis. Angew. Chem. 40, 3791–3794. 10.1002/1521-3773(20011015)40:20<3791::AID-ANIE3791>3.0.CO;2-T 11668535

[B44] Strack D. Vogt T. Schliemann W. (2003). Recent advances in betalain research. Phytochemistry 62, 247–269. 10.1016/S0031-9422(02)00564-2 12620337

[B45] Sullivan M. L. (2015). Beyond brown: polyphenol oxidases as enzymes of plant specialized metabolism. Front. Plant Sci. 5:783. 10.3389/fpls.2014.00783 25642234 PMC4294140

[B46] Svenson J. Smallfield B. M. Joyce N. I. Sanson C. E. Perry N. B. (2008). Betalains in red and yellow varieties of the Andean tuber crop ulluco (*Ullucus tuberosus*). J. Agric. Food Chem. 56, 7730–7737. 10.1021/jf8012053 18662012

[B47] Switala J. Loewen P. C. (2002). Diversity of properties among catalases. Arch. Biochem. Biophys. 401, 145–154. 10.1016/S0003-9861(02)00049-8 12054464

[B48] Tesoriere L. Butera D. Pintaudi A. M. Allegra M. Livrea M. A. (2004). Supplementation with cactus pear (*Opuntia ficus-indica*) fruit decreases oxidative stress in healthy humans: a comparative study with vitamin C. Am. J. Clin. Nutr. 80, 391–395. 15277160 10.1093/ajcn/80.2.391

[B49] Vetrano A. M. Heck D. E. Mariano T. M. Mishin V. Laskin D. L. Laskin J. D. (2005). Characterization of the oxidase activity in mammalian catalase. J. Biol. Chem. 280, 35372–35381. 10.1074/jbc.M503991200 16079130

[B50] Wang C. Song H. Gong X. Hu Q. Liu F. Wang B. (2007b). Correlation of tyrosinase activity and betacyanin biosynthesis induced by dark in C_3_ halophyte *Suaeda salsa* seedlings. Plant Sci. 173, 487–494. 10.1016/j.plantsci.2007.07.010

[B51] Wang C. Q. Chen M. Wang B. S. (2007a). Betacyanin accumulation in the leaves of C_3_ halophyte *Suaeda salsa* L. is induced by watering roots with H_2_O_2_. Plant Sci. 172, 1–7. 10.1016/j.plantsci.2006.06.015

[B52] Wang J. Constabel C. P. (2003). Biochemical characterization of two differentially expressed polyphenol oxidases from hybrid poplar. Phytochemistry 64, 115–121. 10.1016/S0031-9422(03)00159-6 12946410

[B53] Weydert C. J. Cullen J. J. (2010). Measurement of superoxide dismutase, catalase and glutathione peroxidase in cultured cells and tissue. Nat. Protoc. 5, 51–66. 10.1038/nprot.2009.197 20057381 PMC2830880

[B54] Winder A. J. Harris H. (1991). New assays for the tyrosine hydroxylase and dopa oxidase activities of tyrosinase. Eur. J. Biochem. 198, 317–326. 10.1111/j.1432-1033.1991.tb16018.x 1674912

[B55] Yamamoto K. Kobayashi N. Yoshitama K. Teramoto S. Komamine A. (2001). Isolation and purification of tyrosine hydroxylase from callus cultures of *Portulaca grandiflora*. Plant Cell Physiol. 42, 969–975. 10.1093/pcp/pce125 11577191

[B56] Zámocký M. Koller F. (1999). Understanding the structure and function of catalases: clues from molecular evolution and *in vitro* mutagenesis. Prog. Biophys. Mol. Biol. 72, 19–66. 10.1016/S0079-6107(98)00058-3 10446501

