# Supplementary material for: Identification of a Catalase-Phenol Oxidase in Betalain Biosynthesis in Red Amaranth (Amaranthus cruentus)
Source: Front Plant Sci. 2016 Jan 8;6:1228. doi: 10.3389/fpls.2015.01228 (PMC4705222; doi:10.3389/fpls.2015.01228)
Supplement: Supplementary file 1 [file Presentation1.PDF]

Supplementary Material

Identification of a catalase-phenol oxidase in betalain biosynthesis in red amaranth (*Amaranthus cruentus*)

Xiao-Lu Teng, Ning Chen, Xing-Guo Xiao*

*** Correspondence:**
Xing-Guo Xiao, xiaoxg@cau.edu.cn; xiaoxg06@gmail.com

1 Supplementary Data

Here presented 5 Supplementary data.

Supplementary Data 1. K_m value of monophenolase activity (MO) of the purified enzyme toward L-tyrosine calculated from the Lineweaver-Burk graphs

Supplementary Data 2. K_m value of diphenolase activity (DO) of the purified enzyme toward L-DOPA calculated from the Lineweaver-Burk graphs

Supplementary Data 3. Video for in-vitro experiment of AcCATPO catalyzing the degradation of hydrogen peroxide (H_2O_2) and oxidation of L-DOPA
The reaction mixture (550 μL) consisted of the substrate solution containing 310 μL of 50 mM Tris-HCl (pH 7.0), 120 μL of 5 mM L-DOPA, 50 μL of 30% (V/V) H_2O_2, and 70 μL of the purified enzyme. A control test was run in parallel in the absence of the purified enzyme. The reaction was immediately recorded after addition of the purified enzyme.

Supplementary Data 4. K_m value of catalase activity (CAT) of the purified enzyme toward H_2O_2 calculated from the Lineweaver-Burk graphs

Supplementary Data 5. Multiple Sequence Alignment of AcCATPO, bovine and human catalases, and other plant catalases

Sequences were retrieved from a basic local alignment search (BLAST) (National Center for Biotechnology Information, NCBI). The AcCATPO sequence obtained was used as a query selecting results with total score >800 (E value = 0, except partial sequences). Accession codes (GenBank) for sequences from betalain-producing plants (highlighted by yellow) are: *Beta vulgaris* subsp. *vulgaris* 1,2 (gi:731361773, 731361778), *Beta vulgaris* subsp. *Maritime* (gi:564116010), *Mesembryanthemum crystallinum* root, leaf (gi:3202034, 3202032), *Rheum australe* (gi:197312885), *Hylocereus undatus* (gi:571032250), *Bassia scoparia* (gi:686477557), and *Suaeda salsa* (gi:20138726). Accession codes for the non-betalain-producing plants: *Prunus mume* 1,2 (gi:645265094, 645265092), *Gardenia jasminoides* (gi:721750661), *Nicotiana tabacum* 1,2 (gi:429535123, 2459684), *Prunus persica* (gi:32526568), *Nicotiana benthamiana* (gi:219560127), *Vitis vinifera* 1,2,3,4 (gi:526117723, 359476986, 819330654, 731427403), *Nicotiana sylvestris* (gi:698496386), *Prunus avium* (gi:121078773), *Nicotiana tomentosiformis* (gi:697176653), *Solanum tuberosum* (gi:565347640), *Eucalyptus grandis* 1,2 (gi:702368481, 702368496), *Solanum lycopersicum* (gi:854282574),

Tarenaya hassleriana 1,2,3 (gi:729311669, 729453171, 729330417), *Musa acuminata* subsp. *malaccensis* (gi:695050168), *Theobroma cacao* 1,2 (gi:590701975, 590595360), *Sesamum indicum* 1,2,3 (gi:749385032, 747055694, 747055696), *Brassica juncea* 1,2,3,4 (gi:4336754, 4336756, 4336758, 4336752), *Camelina sativa* 1,2,3 (gi:727512115, 727548051, 727523245), *Ipomoea batatas* 1,2 (gi:282935438, 115703), *Raphanus sativus* 1,2 (gi:8050693, 7302765), *Ziziphus jujuba* (gi:357966938), *Brassica rapa* (gi:685256782), *Jatropha curcas* 1,2 (gi:802555337, 806776603), *Nicotiana glutinosa* (gi:2253291), *Arabis alpina* (gi:674237382), *Arabidopsis thaliana* 1,2,3,4 (gi:15236264, 1246399, 15451166, 444340), *Brassica oleracea* (gi:259122789), *Brassica napus* (gi:169244543), *Solanum melongena* (gi:562787), *Genlisea aurea* (gi:527202914), *Gossypium arboretum* 1,2 (gi:728845709, 728833533), *Gossypium hirsutum* (gi:211906480), *Eriobotrya japonica* (gi:442736195), *Gossypium raimondii* (gi:823132870), *Hevea brasiliensis* (gi:315937176), *Elaeis guineensis* (gi:743775712), and *Citrus sinensis* (gi:568839653). Bovine catalase, *Bos taurus* catalase (UniProt accession number, sp|P00432); Human catalase, *Homo sapiens* catalase (UniProt accession number, sp|P04040). The amino acid residuals constituting a putative PPO active site were red-boxed. The amino acid residuals constituting catalase active sites were green-boxed. The putative conserved internal peroxisomal targeting signal was blue-boxed.

2 Supplementary Figures and Tables

Here presented 4 Supplementary Figures and 2 Supplementary Tables.

2.1 Supplementary Figures

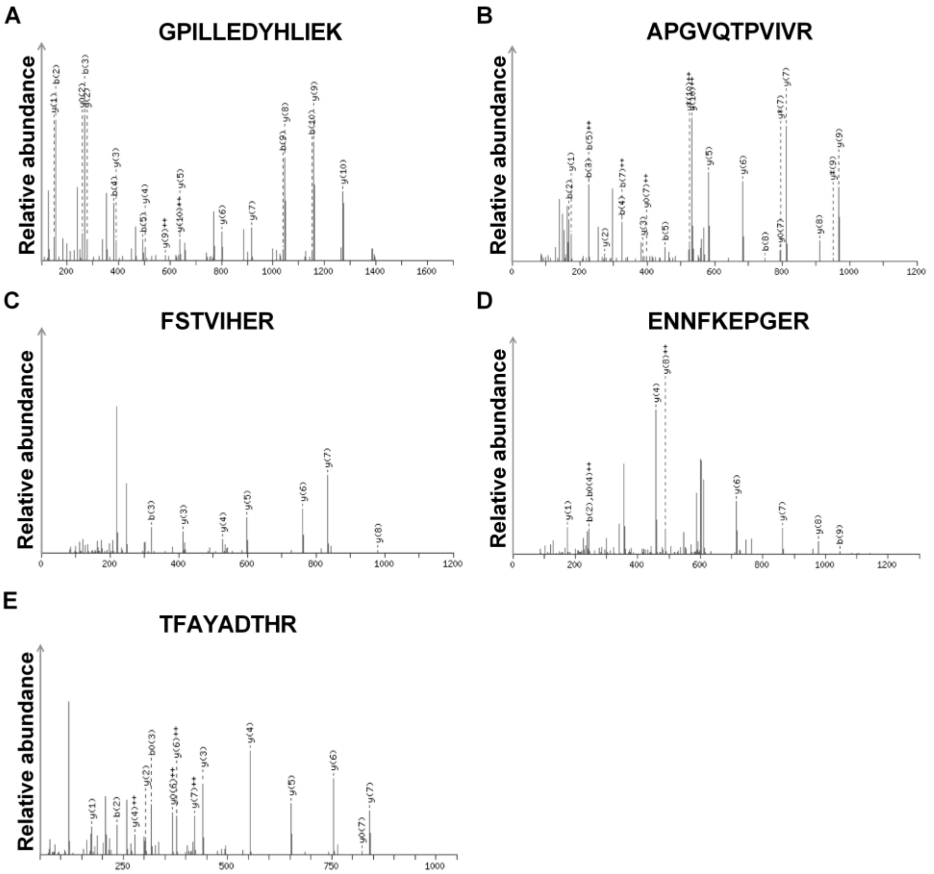

Supplementary Figure S1. MS/MS spectrum of five clear peptide fragments derived by tryptic digestion of the purified enzyme

Additional information regarding to five clear peptides (A-E) was displayed in Supplementary Table S1.

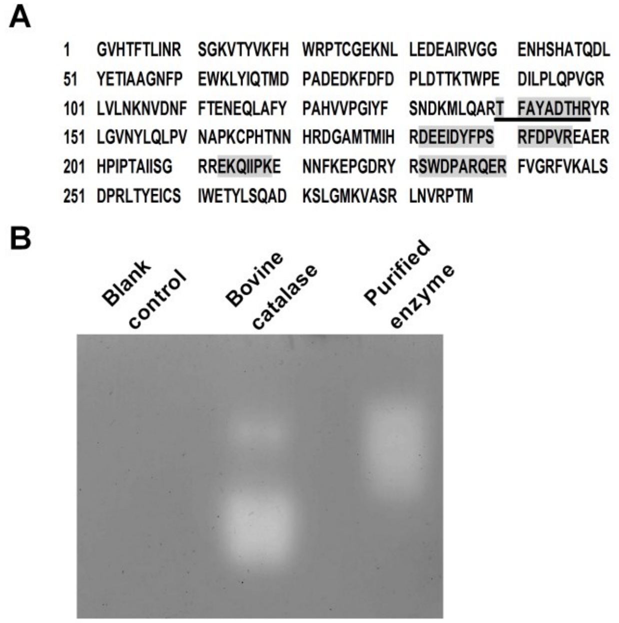

Supplementary Figure S2. Partial characterization of the catalase activity of the purified enzyme

(A) Amino acid sequence coverage of the catalase protein (NCBI accession number, gi|15617223) analyzed by nano-LC-MS/MS. The peptide sequences identical to those identified from MASCOT database were shaded, and the catalase-specific one with extensive homology underlined. (B) In-gel catalase activity assay of the purified enzyme. After electrophoresis, the native PAGE gel with the purified enzyme, bovine catalase (positive control) and buffer only (negative control) was washed with ddH_2O, incubated in H_2O_2, then rinsed once with ddH_2O for 5 min and stained until achromatic bands appeared as shown in (Weydert and Cullen, 2010).

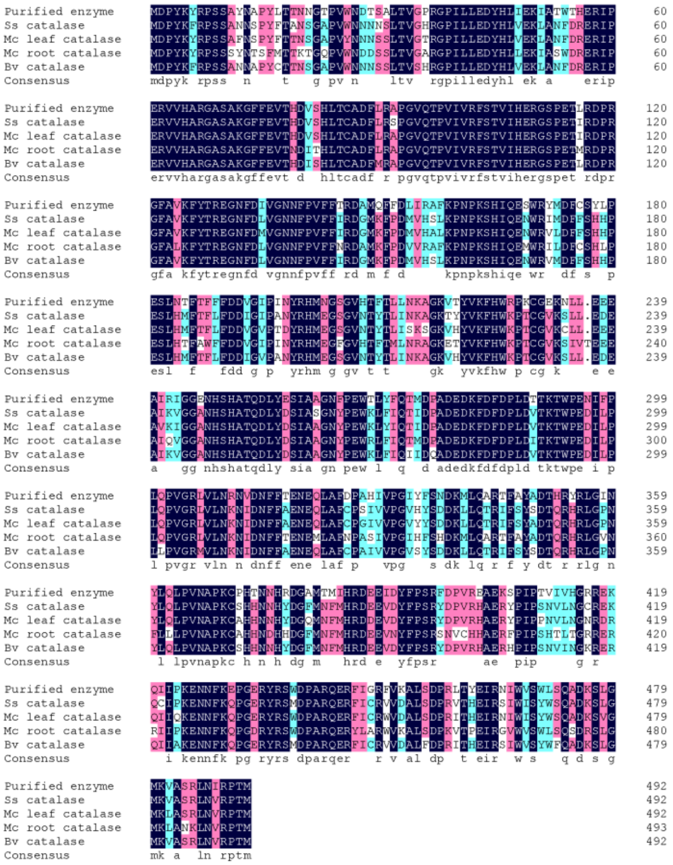

Supplementary Figure S3. Alignment of deduced amino acid sequences of the purified enzyme with those of catalases from some betalain-producing plants

Ss catalase, *Suaeda salsa* catalase (gi|20138726); Mc leaf catalase, *Mesembryanthemum crystallinum* leaf catalase (gi|3202032); Mc root catalase, *Mesembryanthemum crystallinum* root catalase (gi|3202034); Bv catalase, *Beta vulgaris* subsp. *maritima* catalase (gi|564116010).

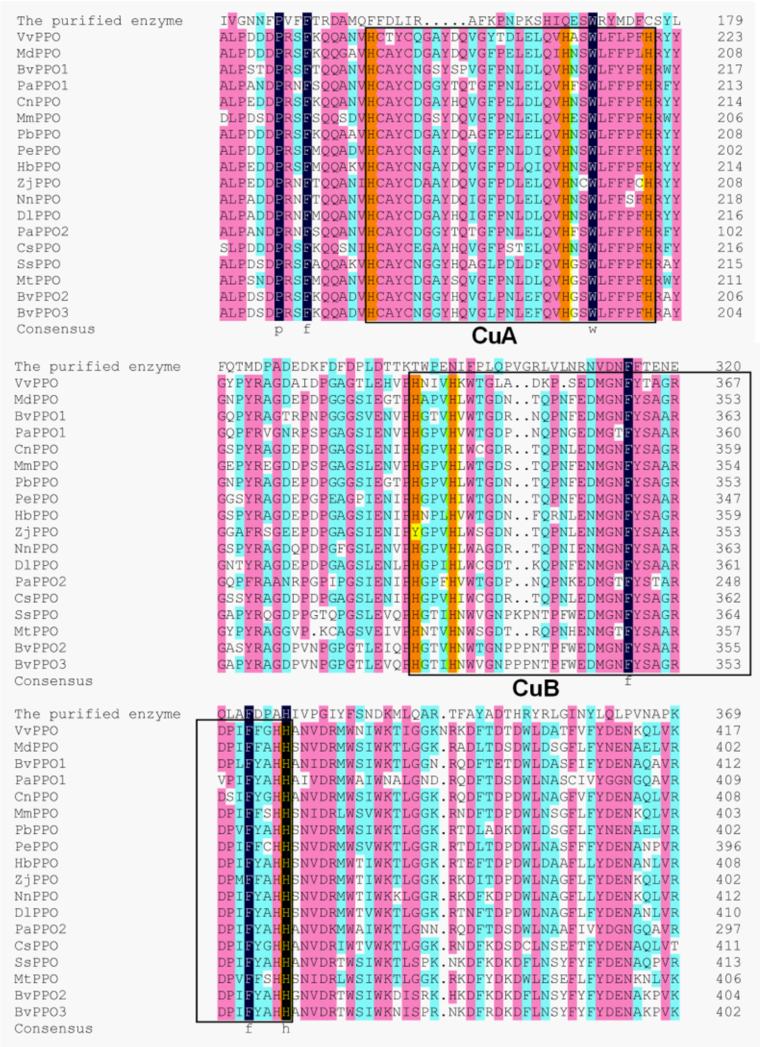

Supplementary Figure S4. Alignment of deduced amino acid sequences of the purified enzyme with those of plant PPOs

Active sites of plant PPOs (CuA and CuB) are highlighted. VvPPO, *Vitis vinifera* polyphenol oxidase (gi|1785613); MdPPO, *Malus domestica* polyphenol oxidase (gi|2293360); BvPPO1,2,3, *Beta vulgaris* subsp. *vulgaris* (gi|731350566, gi|731350560, gi|731350564); PaPPO1,2, *Phytolacca americana* polyphenol oxidase (gi|1752724, gi|1741862); CnPPO, *Camellia nitidissima* polyphenol oxidase (gi|222093457); MmPPO, *Morus alba* var. *multicaulis* polyphenol oxidase (gi|929652374); PbPPO, *Pyrus* x *bretschneideri* polyphenol oxidase (gi|334361506); PePPO, *Populus euphratica* polyphenol oxidase (gi|343157314); HbPPO, *Hevea brasiliensis* polyphenol oxidase (gi|570933638); ZjPPO, *Ziziphus jujuba* polyphenol oxidase (gi|313758569); NnPPO, *Nelumbo nucifera* polyphenol oxidase (gi|289188217); DlPPO, *Dimocarpus longan* polyphenol oxidase (gi|827195154); CsPPO, *Camellia sinensis* polyphenol oxidase (gi|193247172); SsPPO, *Suaeda salsa* polyphenol oxidase (gi|442557135); MtPPO, *Medicago truncatula* polyphenol oxidase (gi|922362506).

2.2 Supplementary Tables

Supplementary Table S1. Peptide sequences obtained by nano-LC-MS/MS analysis

Enzyme [Species] matched	NCBInr GenBank accessions	Peptide sequence
catalase [*Ipomoea batatas*]	gi|282935438	GPILLEDYHLIEK
catalase [*Hevea brasiliensis*]	gi|12002676	APGVQTPVIVR
catalase [*Hevea brasiliensis*]	gi|12002676	FSTVIHER
catalase [*Arabidopsis thaliana*]	gi|1246399	ENNFKEPGER
catalase [*Suaeda salsa*]	gi|15617223	TFAYADTHR

Supplementary Table S2. Comparison of K_m values of tyrosinases from different sources toward L-tyrosine and L-DOPA

Tyrosinase source	K_m values (mM)		Reference
	L-tyrosine	L-DOPA	
Leaves of red amaranth	0.2	0.6	This paper
Betacyanin-producing callus cultures of *Portulaca grandiflora*	1.0	2.4	(Steiner et al., 1999)
Human	0.2	0.4	(Kang and Choi, 1993)
Mouse	0.21	0.45	(Hearing and Jiménez, 1987)
Fly agaric (*Amanita muscaria)*	0.3	1.2	(Mueller et al., 1996)
Button mushroom (*Agaricus bisporus*)	0.3	0.5	(Lejczak et al., 1987)

References

Hearing, V.J., and Jiménez, M. (1987). Mammalian tyrosinase - The critical regulatory control point in melanocyte pigmentation. *International Journal of Biochemistry* 19**,** 1141-1147. doi: http://dx.doi.org/10.1016/0020-711X(87)90095-4.

Kang, S., and Choi, J.-D. (1993). Purification and characterization of human tyrosinase. *Korean Biochemical Journal* 26**,** 632-637.

Lejczak, B., Kafarski, P., and Makowiecka, E. (1987). Phosphonic analogues of tyrosine and 3,4-dihydroxyphenylalanine (dopa) influence mushroom tyrosinase activity. *Biochemical Journal* 242**,** 81-88.

Mueller, L.A., Hinz, U., and Zryd, J.P. (1996). Characterization of a tyrosinase from *Amanita muscaria* involved in betalain biosynthesis. *Phytochemistry* 42**,** 1511-1515. doi: 10.1016/0031-9422(96)00171-9.

Steiner, U., Schliemann, W., Bohm, H., and Strack, D. (1999). Tyrosinase involved in betalain biosynthesis of higher plants. *Planta* 208**,** 114-124. doi: 10.1007/s004250050541.

Weydert, C.J., and Cullen, J.J. (2010). Measurement of superoxide dismutase, catalase and glutathione peroxidase in cultured cells and tissue. *Nature Protocols* 5**,** 51-66. doi: 10.1038/nprot.2009.197.
