# Supplementary material for: Identification of a Catalase-Phenol Oxidase in Betalain Biosynthesis in Red Amaranth (Amaranthus cruentus)
Source: Front Plant Sci. 2016 Jan 8;6:1228. doi: 10.3389/fpls.2015.01228 (PMC4705222; doi:10.3389/fpls.2015.01228)
Supplement: Supplementary file 6 [file DataSheet5.PDF]

```
Amaranthus_cruentus_cv._Hopi_Red_Dye         ........MDPYKYRPS..SAYNAPYLTTNNGTPVWNDTSALTVGPRGPI   40
Bos_taurus                                   MADNRDPASDQMKHWKEQRAAQKPDVLTTGGGNPVGDKLNSLTVGPRGPL   50
Homo_sapiens                                 MADSRDPASDQMQHWKEQRAAQKADVLTTGAGNPVGDKLNVITVGPRGPL   50
Beta_vulgaris_subsp._vulgaris_1              ........MDPTKYRPA..SAYNSPYLTNNNGTPVWNDNSSLTVGPRGPI   40
Mesembryanthemum_crystallinum|root           ........MDPYKYRPS..SSYNTSFMTTKTGQPVWNDDSSLTVGARGPI   40
Mesembryanthemum_crystallinum|leaf           ........MDPYKYRPS..SAFNSPYFTTNSGAPVYNNNSSLTVGTRGPI   40
Prunus_mume_1                                ........MDPYKHRPS..SAFDSPYWTTNAGAPVWNNDSSLTVGPRGPV   40
Gardenia_jasminoides                         ........MDPYKFRPS..SAFNTPFWTTNAGGPVWNNDSALTVGTRGPI   40
Bassia_scoparia                              ........MDPYKFRPS..SAHNSPHFTTNSGAPVWNDNSSMTVGHRGPI   40
Beta_vulgaris_subsp._vulgaris_2              ........MDPYKFRPS..SANNAPYCTTNSGAPVWNNNSSLTVSHRGPI   40
Suaeda_salsa                                 ........MDPYKFRPS..SANNSPYFTANSGAPVWNNNNSLTVGHRGPI   40
Nicotiana_tabacum_1                          ........MDPYKYRPS..SAFNSPFCTTNSGAPVFNNNSSLTVGARGPV   40
Nicotiana_tabacum_2                          ........MDPYKYRPS..SAFNSPFCTTNSGAPVFNNNSSLTVGARGPV   40
Prunus_persica                               ........MDPYKHRPS..SAFDSPYWTTNAGAPVWNNDSSLTVGPRGPV   40
Nicotiana_benthamiana                        ........MDPYKYRPS..SAFNSPFCTTNSGAPVFNNNSSLTVGARGPI   40
Vitis_vinifera_1                             ........MDPYKYRPS..SAYNSPYFTTNAGAPVYNNDSSLTVGSRGPV   40
Nicotiana_sylvestris                         ........MDPYKYRPS..SAFNSPFCTTNSGAPVFNNNSSLTVGARGPV   40
Vitis_vinifera_2                             ........MDPYKYRPS..SAYNSPYFTTNAGAPVYNNDSSLTVGSRGPV   40
Prunus_avium                                 ........MDPYKHRPS..SAFDTPFWTTNSGAPVWNNDSSLTVGSRGPV   40
Vitis_vinifera_3                             ........MDPYKYRPS..SAYNSPYFTTNAGAPVYNNDSSLTVGSRGPV   40
Beta_vulgaris_subsp._maritima                ........MDPYKFRPS..SANNAPYCTTNSGAPVWNNNSSLTVSHRGPI   40
Nicotiana_tomentosiformis                    ........MDPYKYRPS..SAFNSTFCTTNSGAPVFNNNSSLTVGARGPV   40
Solanum_tuberosum                            ........MDPYKYRPS..SAFNSPFCTTNSGAPVWNNNSSLTVGARGPV   40
Eucalyptus_grandis_1                         ........MDPYKYRPS..SAYDSSFWTTNYGAPVWNNDSSLTVGTRGPI   40
Solanum_lycopersicum                         ........MDPYKYRPS..SAFNSPFCTTNSGAPVFNNNSSLTVGARGPV   40
Tarenaya_hassleriana_1                       ........MDPYKYRPS..SSHNSPFFTTNSGAPVWNNNSSMTVGPRGPI   40
Vitis_vinifera_4                             ........MDPYKYRPS..SAYNSPFWTTNSGAPVWNNNSSLTVGPRGPI   40
Musa_acuminata_subsp._malaccensis            ........MDPYKYRPS..SNYNSPFWTTNSGAPVWNNNSSLTVGIRGPI   40
Theobroma_cacao_1                            ........MDPYKYRPS..SAFNSPFWTTNSGAPVYNNNSSLSVGARGPI   40
Sesamum_indicum_1                            ........MDPYKYRPS..SAFNSPFMTTNSGAPVWNNNNSLTVGSRGPI   40
Brassica_juncea_1                            ........MDPYKYRPA..SSYNSPFFTTNSGAPVWNNNSSMTVGPRGPI   40
Hylocereus_undatus                           ........MDPYKHRPS..SSFNTTFWTTNSGAPVWNNTSAMTIGQRGPV   40
Camelina_sativa_1                            ........MDPYKYRPA..SSYNSPFFTTNSGAPVWNNNSSMTVGPRGPI   40
Camelina_sativa_2                            ........MDPYKYRPA..SSYNSPFFTTNSGAPVWNNNSSMTVGPRGPI   40
Ipomoea_batatas_1                            ........MDPSKYRPS..SSFNTPFCTTNSGAPVWNNTCALTVGSRGPI   40
Raphanus_sativus_1                           ........MDPYKYRPA..SSYNSPFFTTNSGAPVWNNNSSMTVGPRGPI   40
Ipomoea_batatas_2                            ........MDPSKYRPS..SSFNTPFCTTNSGAPVWNNTCALTVGSRGPI   40
Camelina_sativa_3                            ........MDPYKYRPA..SSYNSPFFTTNSGAPVWNNNSSMTVGPRGPI   40
Rheum_australe                               ........MDPYKFRPS..SAHDSPYWTTNSGAPVWNNNSSLTVGPRGPI   40
Brassica_juncea_2                            ........MDPYKYRPA..SSYNSPFFTTNSGAPVWNNNSSMTVGPRGPI   40
Ziziphus_jujuba                              ........MDPYKYRPS..SAFNSPYWTTNSGAPIWNNNSSLTVGPRGPI   40
Tarenaya_hassleriana_2                       ........MDPYKYRPS..SSYNSPFFTTNSGAPVWNNNSSMTVGPRGPI   40
Brassica_rapa                                ........MDPYKYRPA..SSYNSPFFTTNSGAPVWNNNSSMTVGPRGPI   40
Jatropha_curcas_1                            ........MDPYKFRPS..SANNSPFFTTNSGAPVWNNNNSLTVGSRGPI   40
Nicotiana_glutinosa                          ........MDPYKYRPS..SAFNSPFCTTNSGAPEKNNNSYLTVGARGLV   40
Brassica_juncea_3                            ........MDPYKYRPA..SSYNSPFFTTNFGAPVWNNNSSMTVGPRGPI   40
Arabis_alpina                                ........MDPYKYRPA..SSYNSPFFTTNSGAPVWNNNSSMTVGPRGPI   40
Eucalyptus_grandis_2                         ........MDPYKYRPS..SAYDSSFWTTNYGAPVWNNDSSLTVGTRGPI   40
Theobroma_cacao_2                            ........MDPYKYRPS..SAFNSPFWTTNSGAPVWNNNSSLTVGPRGPI   40
Arabidopsis_thaliana_1                       ........MDPYKYRPA..SSYNSPFFTTNSGAPVWNNNSSMTVGPRGPI   40
Brassica_oleracea                            ........MDPYKYRPA..SSYNSPFFTTNSGAPVWNNNSSMTVGPRGPI   40
Arabidopsis_thaliana_2                       ........MDPYKYRPA..SSYNSPFFTTNSGAPVWNNNSSMTVGPRGPI   40
Brassica_napus                               ........MDPYKYRPA..SSYNSPFFTTNSGAPVWNNNSSMTVGPRGPI   40
Sesamum_indicum_2                            ........MDPYKYRPS..RAFNSPFMTTNSGAPVWNNNDSLTVGTRGPI   40
Solanum_melongena                            ........MDLSKYRPS..SAYDTPFLTTNAGGPVYNNVSSLTVGPRGPV   40
Sesamum_indicum_3                            ........MDPYKYRPS..RAFNSPFMTTNSGAPVWNNNDSLTVGTRGPI   40
Raphanus_sativus_2                           ........MDPYKYRPA..SSYNSPFFTN.SGAPVWNNNSSMTVGPRGPI   39
Genlisea_aurea                               ........MDPYKYRPS..SAYNSPFWTTNSGAPVWNNNHSLTVGTRGPI   40
Arabidopsis_thaliana_3                       ........MDPYKYRPA..SSYNSPFFTTNSGAPVWNNNSSMTVGPRGPI   40
Gossypium_arboreum_1                         ........MDPYKHRPS..SAFNSPFWTTNSGAPVWNNNSSLTVGPRGPI   40
Gossypium_hirsutum                           ........MDPYKHRPS..SAFNSPFWTTNSGAPVWNNNSSLTVGPRGPI   40
Eriobotrya_japonica                          ........MDPYKYRPS..SAFNSPFWTTNAGAPVWNNNSSLTVGSRGPV   40
Gossypium_raimondii                          ........MDPYKHRPS..SAFNSPFWTTNSGAPVWNNNSSLTVGPRGPI   40
Tarenaya_hassleriana_3                       ........MDPYKYRPS..SAHSSPFFTTNSGAPVWNNNSSLTVGTRGPI   40
Jatropha_curcas_2                            ........MDPYKHCPS..SAHNTPFWTTNAGAPVWNNNSSMTVGQRGPI   40
Hevea_brasiliensis                           ........MDPYKFRPS..SAHNSSFFTTNSGAPVWNNNNSLTVGSRGPI   40
Arabidopsis_thaliana_4                       ........MDPYKYRPA..SSYNSPFFTTNSGAPVWNNNSSMTVGPRGLI   40
Elaeis_guineensis                            ........MDPYKYRPS..SAYNSPFWTTNSGAPVWNNNSSLTVGIRGPI   40
Gossypium_arboreum_2                         ........MDPYKFRPS..SSFDSPFWTTNSGAPVWNNNSSLTVGARGPI   40
Prunus_mume_2                                ........MDPYKYRPS..SAFDSPFWTTNSGAPVWNNNSSLTVGSRGPI   40
Citrus_sinensis                              ........MDPYKFRPA..SSFNAPFFTTNSGAPVWNNNSSMTIGPRGPI   40
Brassica_juncea_4                            ........MDPYKYRPA..SSYNSPFFTTNSGAPVWNNNSSMTVGPRGPI   40
Consensus                                             d                 t   g p            rg
```

```
Amaranthus_cruentus_cv._Hopi_Red_Dye     LLEDYHLIEKIATWTHERIPERVVHARGASAKGFFEVTHDVSHLTCADFL   90
Bos_taurus                               LVQDVVFTDEMAHFDRERIPERVVHAKGAGAFGYFEVTHDITRYSKAKVF  100
Homo_sapiens                             LVQDVVFTDEMAHFDRERIPERVVHAKGAGAFGYFEVTHDITKYSKAKVF  100
Beta_vulgaris_subsp._vulgaris_1          LLEDYHLIEKIATWTHERIPERVVHARGASAKGFFEVTHDIAHLTCADFL   90
Mesembryanthemum_crystallinum|root       LLEDYHLLEKIASWDRERIPERVVHARGASAKGFFEVTNDITHLTCADFL   90
Mesembryanthemum_crystallinum|leaf       LLEDYHLVEKLANFDRERIPERVVHARGASAKGFFEVTHDVSHLTCADFL   90
Prunus_mume_1                            LLEDYHLVEKLATFDRERIPERVVHARGASAKGFFEVTHDISQLTCADFL   90
Gardenia_jasminoides                     LLEDYHLVEKLANWTRERIPERVVHARGASAKGFFEVTHDISNLTCADFL   90
Bassia_scoparia                          LLEDYHLVEKLANFDRERIPERVVHARGASAKGFFETTHDISHLTCADFL   90
Beta_vulgaris_subsp._vulgaris_2          LLEDYHLVEKLANFDRERIPERVVHARGASAKGFFEVTHDISHLTCADFM   90
Suaeda_salsa                             LLEDYHLVEKLANFDRERIPERVVHARGASAKGFFEVTHDVSHLTCADFL   90
Nicotiana_tabacum_1                      LLEDYHLVEKLANFDRERVPERVVHARGASAKGFFEVTHDITHLTCADFL   90
Nicotiana_tabacum_2                      LLEDYHLVEKLANFDRERVPERVVHARGASAKGFFEVTHDITHLTCADFL   90
Prunus_persica                           LLEDYHLVEKLATFDRERIPERVVHARGASAKGFFEVTHDISQLTCADFL   90
Nicotiana_benthamiana                    LLEDYHLVEKLANFDRERVPERVVHARGASAKGFFEVTHDITHLTCADFL   90
Vitis_vinifera_1                         LLEDYHLVEKLANFQRERIPERVVHARGASAKGFFEVTHDVSDLTCADFL   90
Nicotiana_sylvestris                     LLEDYHLVEKLANFDRERVPERVVHARGASAKGFFEVTHDITHLTCADFL   90
Vitis_vinifera_2                         LLEDYHLVEKLANFQRERIPERVVHARGASAKGFFEVTHDVSDLTCADFL   90
Prunus_avium                             LLEDYHLVEKLATFDRERIPERVVHARGASAKGFFEVTHDISQLTCADFL   90
Vitis_vinifera_3                         LLEDYHLVEKLANFQRERIPERVVHARGASAKGFFEVTHDVSDLTCADFL   90
Beta_vulgaris_subsp._maritima            LLEDYHLVEKLANFDRERIPERVVHARGASAKGFFEVTHDISHLTCADFM   90
Nicotiana_tomentosiformis                LLEDYHLVEKLANFDRERVPERVVHARGASAKGFFEVTHDITHLTCADFL   90
Solanum_tuberosum                        LLEDYHLVEKLANFDRERIAERVVHARGASAKGFFEVTHDIAHLTCADFL   90
Eucalyptus_grandis_1                     LLEDYHLIEKLANFERERIPERVVHARGASAKGFFEVTHDISHLTCADFL   90
Solanum_lycopersicum                     LLEDYHLVEKLANFDRERIAERVVHARGASAKGFFEVTHDIAHLTCADFL   90
Tarenaya_hassleriana_1                   LLEDYHLVEKLANFDRERIPERVVHARGASAKGFFEVTHDISHLTCADFL   90
Vitis_vinifera_4                         LLEDYHLVEKLANFDRERIPERVVHARGASAKGFFETTHDISNLTCADFL   90
Musa_acuminata_subsp._malaccensis        LLEDYHLVEKLAQFDRERIPERVVHARGASAKGFFEVTHDVSHLTCADFL   90
Theobroma_cacao_1                        LLEDYHLVEKLANFDRERIPERVVHARGASAKGFFEVTHDISHLTCADFL   90
Sesamum_indicum_1                        LLEDYHLVEKLANFDRERIPERVVHARGASAKGFFEVTHDISHLSCADFL   90
Brassica_juncea_1                        LLEDYHLVEKLANFDRERIPERVVHARGASAKGFFEVTHDISNLTCADFL   90
Hylocereus_undatus                       LLEDYHLIEKITNFHRERIPERIVHARGASAKGFFEVTHDVSHLTCADFL   90
Camelina_sativa_1                        LLEDYHLVEKLANFDRERIPERVVHARGASAKGFFEVTHDISNLTCADFL   90
Camelina_sativa_2                        LLEDYHLVEKLANFDRERIPERVVHARGASAKGFFEVTHDISNLTCADFL   90
Ipomoea_batatas_1                        LLEDYHLIEKIQNFTRERIPERVVHARGATAKGFFEVTHDITHLTCADFL   90
Raphanus_sativus_1                       LLEDYHLVEKLANFDRERIPERVVHARGASAKGFFEVTHDISNLTCADFL   90
Ipomoea_batatas_2                        LLEDYHLVEKIQNFTRERIPERVVHARGASAKGFFEVTHDITHLTCADFL   90
Camelina_sativa_3                        LLEDYHLVEKLANFDRERIPERVVHARGASAKGFFEVTHDISNLTCADFL   90
Rheum_australe                           LLEDYHLVEKIVNFDRERIPERVVHARGASAKVFFEVTHDVSHLTCADFL   90
Brassica_juncea_2                        LLEDYHLVEKLANFDRERIPERVVHARGASAKGFFEVTHDISNLTCADFL   90
Ziziphus_jujuba                          LLEDYHLVEKLANFDRERIPERVVHARGASAKGFFEVTHDISHLTCADFL   90
Tarenaya_hassleriana_2                   LLEDYHLVEKLANFDRERIPERVVHARGASAKGFFEVTHDISHLTCADFL   90
Brassica_rapa                            LLEDYHLVEKLANFDRERIPERVVHARGASAKGFFEVTHDISNLTCADFL   90
Jatropha_curcas_1                        LLEDYHLVEKLANFDRERIPERVVHARGASAKGFFEVTHDISHLTCADFL   90
Nicotiana_glutinosa                      LLEDYHLVEKLANFDRERVPERVVHARGASAKGFFEVTHDITHLTCADFL   90
Brassica_juncea_3                        LLEDYHLVEKLANFDRERIPERVVHARGASAKGFFEVTHDISNLTCADFL   90
Arabis_alpina                            LLEDYHLVEKLANFDRERIPERVVHARGASAKGFFEVTHDISNLTCADFL   90
Eucalyptus_grandis_2                     LLEDYHLLEKLANFERERIPERVVHARGASAKGFFEVTHDISHLTCADFL   90
Theobroma_cacao_2                        LLEDYHLVEKLANFDRERIPERVVHARGASAKGFFEVTHDISHLTCADFL   90
Arabidopsis_thaliana_1                   LLEDYHLVEKLANFDRERIPERVVHARGASAKGFFEVTHDISNLTCADFL   90
Brassica_oleracea                        LLEDYHLVEKLANFDRERIPERVVHARGASAKGFFEVTHDISNLTCADFL   90
Arabidopsis_thaliana_2                   LLEDYHLVEKLANFDRERIPERVVHARGASAKGFFEVTHDISNLTCADFL   90
Brassica_napus                           LLEDYHLVEKLANFDRERIPERVVHARGASAKGFFEVTHDISNLTCADFL   90
Sesamum_indicum_2                        LLEDYHLVEKLANFDRERIPERVVHARGASAKGFFEVTHDISHLTCADFL   90
Solanum_melongena                        LLEDYHLIEKLATFDRERIPERVVHARGASAKGFFEVTHDVSHLTCADFL   90
Sesamum_indicum_3                        LLEDYHLVEKLANFDRERIPERVVHARGASAKGFFEVTHDISHLTCADFL   90
Raphanus_sativus_2                       LLEDYHLVEKLANFDRERIPERVVHARGASAKGFFEVTHDISNLTCADFL   89
Genlisea_aurea                           LLEDYHLVEKLANFDRERIPERVVHARGASAKGFFEVTHDISHLTSADFL   90
Arabidopsis_thaliana_3                   LLEDYHLVEKLANFDRERIPERVVHARGASAKGFFEVTHDISNLTCADFL   90
Gossypium_arboreum_1                     LLEDYHLVEKLANFDRERIPERVVHARGASAKGFFEVTHDISHLTCADFL   90
Gossypium_hirsutum                       LLEDYHLVEKLANFDRERIPERVVHARGASAKGFFEVTHDISHVTCADFL   90
Eriobotrya_japonica                      LLEDYHLIEKLANFDRERIPERVVHARGASAKGFFEVTHDISHLSCADFL   90
Gossypium_raimondii                      LLEDYHLVEKLANFDRERIPERVVHARGASAKGFFEVTHDISHLTCADFL   90
Tarenaya_hassleriana_3                   LLEDYHLLEKLANFDRERIPERVVHARGASAKGFFEVTHDITHLTCADFL   90
Jatropha_curcas_2                        LLEDYHMLEKLANFDRERIPERVVHARGMSAKGFFEVTHEISNLTCADFL   90
Hevea_brasiliensis                       LLEDYHLVEKLANFDRERIPERVVHARGASSKGFFEVTHDISHLTCADFL   90
Arabidopsis_thaliana_4                   LLEDYHLVEKLANFDRERIPERVVHARGASAKGFFEVTHDISNLTCADFL   90
Elaeis_guineensis                        LLEDYHVIEKLAQFDRERIPERVVHARGASAKGFFEVTHDVSHLTCADFL   90
Gossypium_arboreum_2                     LLEDYHLVEKLANFDRERIPERVVHARGASAKGFFEVTHDISQLTCADFL   90
Prunus_mume_2                            LLEDYHLVEKIANFDRERIPERVVHARGASAKGFFEVTHDISQLTCADFL   90
Citrus_sinensis                          LLEDYHLVEKLANFDRERIPERVVHARGASAKGFFEVTHDVSNLTCADFL   90
Brassica_juncea_4                        LLEDYHLVEKLANFDRERIPERVVHARGASAKGFFEVTHDISNLTCADFL   90
Consensus                                l  d            er  er vha g      fe t       a
```

```
Amaranthus_cruentus_cv._Hopi_Red_Dye     RAPGVQTPVIVRFSTVIHERGSPETLRDPRGFAVKFYTREGNFDIVGNNF   140
Bos_taurus                               EHIGKRTPIAVRFSTVAGESGSADTVRDPRGFAVKFYTEDGNWDLVGNNT   150
Homo_sapiens                             EHIGKKTPIAVRFSTVAGESGSADTVRDPRGFAVKFYTEDGNWDLVGNNT   150
Beta_vulgaris_subsp._vulgaris_1          RSPGVQTPVIVRFSTVIHERGSPETLRDPRGFAVKFYTREGNFDIVGNNF   140
Mesembryanthemum_crystallinum|root       RAPGVQTPVIVRFSTVIHERGSPETMRDPRGFALKFYTREGNFDLVGNNF   140
Mesembryanthemum_crystallinum|leaf       RAPGVQTPVIVRFSTVIHERGSPETIRDPRGFAVKFYTREGNFDMVGNNF   140
Prunus_mume_1                            RAPGVQTPVIVRFSTVIHERGSPETLRDPRGFAVKFYTREGNFDLVGNNF   140
Gardenia_jasminoides                     RAPGVQTPVIVRFSTVIHERGSPETLRDPRGFAVKFYTREGNYDLVGNNF   140
Bassia_scoparia                          RSPGVQTPVIVRFSTVIHERGSPETIRDPRGFAVKFYTREGNFDLVGNNF   140
Beta_vulgaris_subsp._vulgaris_2          RAPGVQTPVIVRFSTVIHERGSPETLRDPRGFAVKFYTREGNFDLVGNNF   140
Suaeda_salsa                             RSPGVQTPVIVRFSTVIHERGSPETIRDPRGFAVKFYTREGNFDLVGNNF   140
Nicotiana_tabacum_1                      RAPGVQTPVIVRFSTVIHERGSPETLRDPRGFAVKFYTREGNFDLVGNNF   140
Nicotiana_tabacum_2                      RAPGVQTPVIVRFSTVIHERGSPETLRDPRGFAVKFYTREGNFDLVGNNF   140
Prunus_persica                           RAPGVQTPVIVRFSTVIHERGSPETLRDPRGFAVKFYTREGNFDLVGNNF   140
Nicotiana_benthamiana                    RAPGVQTPVIVRFSTVIHERGSPETLRDPRGFAVKFYTREGNFDLVGNNF   140
Vitis_vinifera_1                         RAPGVQTPVIVRFSTVIHERGSPETIRDPRGFAVKFYTREGNFDMVGNNF   140
Nicotiana_sylvestris                     RAPGVQTPVIVRFSTVIHERGSPETLRDPRGFAVKFYTREGNFDLVGNNF   140
Vitis_vinifera_2                         RAPGVQTPVIVRFSTVIHERGSPETIRDPRGFAVKFYTREGNFDMVGNNF   140
Prunus_avium                             RAPGVQTPVIVRFSTVIHERGSPETLRDPRGFAVKFYTREGNFDLVGNNF   140
Vitis_vinifera_3                         RTPGVQTPVIVRFSTVIHERGSPETIRDPRGFAVKFYTREGNFDMVGNNF   140
Beta_vulgaris_subsp._maritima            RAPGVQTPVIVRFSTVIHERGSPETLRDPRGFAVKFYTREGNFDLVGNNF   140
Nicotiana_tomentosiformis                RAPGVQTPVIVRFSTVIHERGSPETLRDPRGFAVKFYTREGNFDLVGNNF   140
Solanum_tuberosum                        RAPGVQTPVIVRFSTVIHERGSPETLRDPRGFAVKFYTREGNFDLVGNNF   140
Eucalyptus_grandis_1                     RAPGVQTPVIVRFSTVIHERGSPETLRDPRGFAVKFYTREGNFDLVGNNF   140
Solanum_lycopersicum                     RAPGVQTPVIVRFSTVIHERGSPETLRDPRGFAVKFYTREGNFDLVGNNF   140
Tarenaya_hassleriana_1                   RAPGVQTPVIVRFSTVIHERGSPETLRDPRGFAVKFYTREGNFDLVGNNF   140
Vitis_vinifera_4                         RAPGVQTPVILRFSTVIHERGSPETLRDPRGFAVKFYTREGNFDLVGNNF   140
Musa_acuminata_subsp._malaccensis        RAPGVQTPIIVRFSTVIHERGSPETLRDPRGFAVKFYTREGNFDLVGNNF   140
Theobroma_cacao_1                        RAPGVQTPVIVRFSTVIHERGSPETLRDPRGFAVKFYTREGNFDLVGNNF   140
Sesamum_indicum_1                        RAPGVQTPVIVRFSTVIHERGSPETLRDPRGFAVKFYTREGNFDLVGNNF   140
Brassica_juncea_1                        RAPGVQTPVIVRFSTVIHERGSPETLRDPRGFAVKFYTREGNFDLVGNNF   140
Hylocereus_undatus                       RAPGVQTPVIVRFSTVIHERGSPETIRDPRGFATKFYTREGNFDIVGNNF   140
Camelina_sativa_1                        RAPGVQTPLIVRFSTVIHERGSPETLRDPRGFAVKFYTREGNFDLVGNNF   140
Camelina_sativa_2                        RAPGVQTPLIVRFSTVIHERGSPETLRDPRGFAVKFYTREGNFDLVGNNF   140
Ipomoea_batatas_1                        RAPGVQTPLIVRFSTVIHERGSPETIRDPRGFAVKMYTREGNWDLVGNNF   140
Raphanus_sativus_1                       RAPGVQTPVIVRFSTVIHERGSPETLRDPRGFAVKFYTREGNFDLVGNNF   140
Ipomoea_batatas_2                        RAPGVQTPLIVRFSTVIHERGSPETIRDPRGFAVKMYTRGGNWDLVGNNF   140
Camelina_sativa_3                        RAPGVQTPLIVRFSTVIHERGSPETLRDPRGFAVKFYTREGNFDLVGNNF   140
Rheum_australe                           RAPGVQTPLIVRFSTVIHERGSPETLRDPRGFAVKFYTREGNFDLVGNNF   140
Brassica_juncea_2                        RAPGVQTPVIVRFSTVIHERGSPETLRDPRGFAVKFYTREGNFDLVGNNF   140
Ziziphus_jujuba                          RAPGVQTPVIVRFSTVIHERGSPETLRDPRGFAVKFYTREGNFDLVGNNF   140
Tarenaya_hassleriana_2                   RGPGVQTPVIVRFSTVIHERGSPETLRDPRGFAVKFYTREGNFDLVGNNF   140
Brassica_rapa                            RAPGVQTPLIVRFSTVIHERGSPETLRDPRGFAVKFYTREGNFDLVGNNI   140
Jatropha_curcas_1                        RAPGVQTPVIVRFSTVIHERGSPETLRDPRGFAVKFYTREGNFDLVGNNF   140
Nicotiana_glutinosa                      RAPGVQTPVIVRFSTVIHERGSPETLRDPRGFAVKFYTREGNFDLVGNNF   140
Brassica_juncea_3                        RAPGVQTPVIVRFSTVIHERGSPETLRDPRGFAVKFYTREGNFDLVGNNF   140
Arabis_alpina                            RAPGVQTPVIVRFSTVIHERGSPETLRDPRGFAVKFYTREGNFDLVGNNF   140
Eucalyptus_grandis_2                     RAPGVQTPVIVRFSTVIHERGSPETLRDPRGFAVKFYTREGNFDLVGINF   140
Theobroma_cacao_2                        RAPGVQTPVIVRFSTVIHERGSPETLRDPRGFAVKFYTREGNFDLVGNNF   140
Arabidopsis_thaliana_1                   RAPGVQTPVIVRFSTVIHERGSPETLRDPRGFAVKFYTREGNFDLVGNNF   140
Brassica_oleracea                        RAPGVQTPVIVRFSTVIHERGSPETLRDPRGFAVKFYTREGNFDLVGNNF   140
Arabidopsis_thaliana_2                   RAPGVQTPVIVRFSTVIHERGSPETLRDPRGFAVKFYTREGNFDLVGNNF   140
Brassica_napus                           RAPGVQTPVIVRFSTVIHERGSPETLRDPRGFAVKSYTREGNFDLVGNNF   140
Sesamum_indicum_2                        RAPGVQTPVIVRFSTVIHERGSPETLRDPRGFAVKFYTREGNFDLVGNNF   140
Solanum_melongena                        RAPGVQTPVIQRFSTVVHERGSPESIRDIRGFAVKFYTREGNFDLVGNNV   140
Sesamum_indicum_3                        RAPGVQTPVIVRFSTVIHERGSPETLRDPRGFAVKFYTREGNFDLVGNNF   140
Raphanus_sativus_2                       RAPGVQTPVIVRFSTVIHERGSPETLRDPRGFAVKFYTREGNFDLVGNNF   139
Genlisea_aurea                           RAPGVQTPVIVRFSTVIHERGSPETLRDPRGFAVKFYTREGNFDLVGNNF   140
Arabidopsis_thaliana_3                   RAPGVQTPVIVRFSTVIHERGSPETLRDPRGFAVKFYTREGNFDLVGNNF   140
Gossypium_arboreum_1                     RAPGVQTPVILRFSTVIHERGSPETLRDPRGFAVKFYTREGNFDLVGNNF   140
Gossypium_hirsutum                       RAPGVQTPVILRFSTVIHERGSPETLRDPRGFAVKFYTREGNFDLVGNNF   140
Eriobotrya_japonica                      RAPGVQTPVIVRFSTVIHERGSPETIRDPRGFAVKFYTREGNFDLVGNNF   140
Gossypium_raimondii                      RAPGVQTPVIVRFSTVIHERGSPETLRDPRGFAVKFYTREGNFDLVGNNF   140
Tarenaya_hassleriana_3                   RAPGVQTPVIVRFSTVIHERASPETLRDPRGFAVKFYTREGNFDLVGNNF   140
Jatropha_curcas_2                        RAPGVQTPVIVRFSTVIHERGSPETLRDPRGFATKFYTREGNFDIVGNNF   140
Hevea_brasiliensis                       RAPGVQTPVIVRFSTVIHERGSPETLRDPRGFAVKFYTREGNFDLVGNNF   140
Arabidopsis_thaliana_4                   RAPGVQTPVIVRFSTVIHERGSPETLRDPRGFAVKFYTREGNFDLVGNNF   140
Elaeis_guineensis                        RAPGVQTPVIVRFSTVIHERGSPETLRDPRGFAVKFYTREGNFDLVGNNF   140
Gossypium_arboreum_2                     RAPGVQTPLIVRFSTVIHERGSPETLRDPRGFAVKFYTREGNFDLVGNNF   140
Prunus_mume_2                            RAPGVQTPVIVRFSTVIHERGSPETLRDPRGFAVKFYTREGNFDLVGNNF   140
Citrus_sinensis                          RAPGVQTPVIVRFSTVIHERGSPETLRDPRGFAVKFYTREGNFDLVGNNF   140
Brassica_juncea_4                        RAPGVQTPVIVRFSTVIHERGSPETLRDPRGFAVKFYTREGNFDLVGNNF   140
Consensus                                   g  tp   rfstv  e  s    rd rgfa k yt  gn d vg n
```

```
Amaranthus_cruentus_cv._Hopi_Red_Dye     PVFFTRDAMQFFDLIRAFKPNPKSHIQESWRYMDFCSYLPESLNTFTFFF   190
Bos_taurus                               PIFFIRDALLFPSFIHSQKRNPQTHLKDPDMVWDFWSLRPESLHQVSFLF   200
Homo_sapiens                             PIFFIRDPILFPSFIHSQKRNPQTHLKDPDMVWDFWSLRPESLHQVSFLF   200
Beta_vulgaris_subsp._vulgaris_1          PVFFTRDAMQFFDLVRAFKPNPKSHIQESWRYMDFCSYLPESLNTFSFFF   190
Mesembryanthemum_crystallinum|root       PVFFNRDAMKFPDVVRAFKPNPKSHIQEMWRILDFCSHLPESLHTFAWFF   190
Mesembryanthemum_crystallinum|leaf       PVFFIRDGMKFPDMVHALKPNPKSHIQENWRVLDFFSHHPESLHMFTFLF   190
Prunus_mume_1                            PVFFVRDAMKFPDAIRAFKPNPKSHIQETWRILDFFSHLPESLHTFAFFY   190
Gardenia_jasminoides                     PVFFVRDAMKFPDSIHALKPNPKSHIQESWRILDFFSHLPESLSTFAWFY   190
Bassia_scoparia                          PVFFIRDGMKFPDMVHSLKPNPKSHIQENWRIMDFFSHHPESLHMFTFLF   190
Beta_vulgaris_subsp._vulgaris_2          PVFFIRDGMKFPDMVHSLKPNPKSHIQENWRVMDFFSHHPESLHMFTFLF   190
Suaeda_salsa                             PVFFIRDGMKFPDMVHSLKPNPKSHIQENWRIMDFFSHHPESLHMFTFLF   190
Nicotiana_tabacum_1                      PVFFIRDGMKFPDMVHALKPNPKSHIQENWRVLDFFSHVPESLHMFTFLF   190
Nicotiana_tabacum_2                      PVFFIRDGMKFPDMVHALKPNPKSHIQENWRVLDFFSHVPESLHMFTFLF   190
Prunus_persica                           PVFFVRDAMKFPDAIRAFKPNPKSHIQETWRILDFFSHLPESLHTFAFFY   190
Nicotiana_benthamiana                    PVFFIRDGMKFPDMVHALKPNPKSHIQENWRVLDFFSHVPESLHMFTFLF   190
Vitis_vinifera_1                         PVFFIRDAMKFPDVIHAFKPNPKSHIQEYWRVVDFLSYHPESLSTFAFLF   190
Nicotiana_sylvestris                     PVFFIRDGMKFPDMVHALKPNPKSHIQENWRVLDFFSHVPESLHMFTFLF   190
Vitis_vinifera_2                         PVFFIRDAMKFPDVIHAFKPNPKSHIQEYWRVVDFLSYHPESLSTFAFLF   190
Prunus_avium                             PVFFVRDAMKFPDAIRAFKPNPKSHIQENWRILDFFSHLPESLHTFAFFY   190
Vitis_vinifera_3                         PVFFIRDAMKFPDVIHAFKPNPKSHIQEYWRVVDFLSYHPESLSTFAFLF   190
Beta_vulgaris_subsp._maritima            PVFFIRDGMKFPDMVHSLKPNPKSHIQENWRVMDFFSHHPESLHMFTFLF   190
Nicotiana_tomentosiformis                PVFFIRDGMKFPDMVHALKPNPKSHIQENWRVLDFFSHVPESLHMFTFLF   190
Solanum_tuberosum                        PVFFIRDGMKFPDMVHALKPNPKSHIQENWRVLDFFSHHPESLHMFTFLF   190
Eucalyptus_grandis_1                     PVFFVRDAMKFPDAIHAFKPNPKSNIQEMWRIIDFFSHQPESLSTFAWFF   190
Solanum_lycopersicum                     PVFFIRDGMKFPDMVHALKPNPKSHIQENWRVLDFFSHHPESLHMFTFLF   190
Tarenaya_hassleriana_1                   PVFFIRDGMKFPDMVHALKPNPKSHIQENWRVLDFFSHHPESLHMFTFLF   190
Vitis_vinifera_4                         PVFFIRDGMKFPDMVHALKPNPKSHIQENWRIVDFFSHHPESLHMFSFLF   190
Musa_acuminata_subsp._malaccensis        PVFFIRDGMKFPDMVHALKPNPKSHIQENWRILDFFSHHPESLHMFSFLF   190
Theobroma_cacao_1                        PVFFIRDGMKFPDMVHALKPNPKSHIQENWRILDFFSHHPESLHMFTFLF   190
Sesamum_indicum_1                        PVFFIRDGMKFPDMVHALKPNPKSHIQENWRIMDFFSHHPESLNMFTFLF   190
Brassica_juncea_1                        PVFFIRDGMKFPDMVHALKPNPKSHIQENWRVLDFFSHHPESLNMFTFLF   190
Hylocereus_undatus                       PVFFIRDGMKFPDVIHAFKPNPKSHIQEYWRVLDFCSHHPESLNTFGWLF   190
Camelina_sativa_1                        PVFFIRDGMKFPDMVHALKPNPKSHIQENWRILDFFSHHPESLNMFTFLF   190
Camelina_sativa_2                        PVFFIRDGMKFPDMVHALKPNPKSHIQENWRILDFFSHHPESLNMFTFLF   190
Ipomoea_batatas_1                        PVFFIRDGTQFPDVIHAFKPNPKSHIQENWRILDYLSHLPESLNTFAWFY   190
Raphanus_sativus_1                       PVFFIRDGMKFPDMVHALKPNPKSHIQENWRVLDFFSHHPESLNMFTFLF   190
Ipomoea_batatas_2                        PVFFIRDGTQFPDVIHAFKPNPKSHIQENWRILDYLSHLPESLNTFAWFY   190
Camelina_sativa_3                        PVFFIRDGMKFPDMVHALKPNPKSHIQENWRILDFFSHHPESLNMFTFLF   190
Rheum_australe                           PVFFIRDGMKFPDMVHALKPNPKSHIQENWRILDFFSHHPESLNMFTFLF   190
Brassica_juncea_2                        PVFFIRDGMKFPDMVHALKPNPKSHIQENWRVLDFFSHHPESLNMFTFLF   190
Ziziphus_jujuba                          PVFFIRDGMKFPDMVHALKPNPKSHIQENWRIVDFFSHHPESLHMFTFLF   190
Tarenaya_hassleriana_2                   PVFFIRDGMKFPDMVHALKPNPKSHIQENWRVLDFFSHHPESLHMFTFLF   190
Brassica_rapa                            PVFFIRDGMKFPDMVHALKPNPKSHIQENWRVMDFFSPHPESLNMFTFLF   190
Jatropha_curcas_1                        PVFFIRDGMKFPDMVHALKPNPKSHIQESWRILDFFSHHPESLNMFTFLL   190
Nicotiana_glutinosa                      PVFFIRDGMKFPDMVHALKPNPKSHIQENWRVLDFFSHVPESLHMFTFLF   190
Brassica_juncea_3                        PVFFIRDGMKFPDMVHALKPNPKSHIQENWRVLDFFSHHPESLNMFTFLF   190
Arabis_alpina                            PVFFIRDGMKFPDMVHALKPNPKSHIQENWRVLDFFSHHPESLNMFTFLF   190
Eucalyptus_grandis_2                     PVFFIRDAMKFPDAIHAFKPNPKSNIQEMWRIIDFLSHQPESLSTFAWFF   190
Theobroma_cacao_2                        PVFFVRDGMKFPDMVHALKPNPKSHIQENWRILDFFSHHPESLHMFTFLF   190
Arabidopsis_thaliana_1                   PVFFIRDGMKFPDMVHALKPNPKSHIQENWRILDFFSHHPESLNMFTFLF   190
Brassica_oleracea                        PVFFIRDGMKFPDMVHALKPNPKSHIQENWGVLDFFSHHPESLNMFTFLF   190
Arabidopsis_thaliana_2                   PVFFIRDGMKFPDMVHALKPNPKSHIQENWRILDFFSHHPESLNMFTFLF   190
Brassica_napus                           PVFFIRDGMKFPDMVHALKPNPKSHIQENWRVLDFFSHHPESLNMFTFLF   190
Sesamum_indicum_2                        PVFFVRDGMKFPDMVHALKPNPKSHIQENWRILDFFSHHPESLHMFSFLF   190
Solanum_melongena                        PVFFNRDAKSFPDTIRALKPNPKSHIQENWRILDFFSFLPESLHTFAFFY   190
Sesamum_indicum_3                        PVFFVRDGMKFPDMVHALKPNPKSHIQENWRILDFFSHHPESLHMFSFLF   190
Raphanus_sativus_2                       PVFFIRDGMKFPDMVHALKPNPKSHIQENWRVLDFFSHHPESLNMFTFLF   189
Genlisea_aurea                           PVFFVRDGMKFPDMVHALKPNPRSHIQENWRILDFFSHHPESLHMFTFLF   190
Arabidopsis_thaliana_3                   PVFFIRDGMKFPDMVHALKPNPKSHIQENWRILDFFSHHPESLNMFTFLF   190
Gossypium_arboreum_1                     PVFFIRDGMKFPDMVHALKPNPKSHIQENWRILDFFSHHPESLHMFTFLF   190
Gossypium_hirsutum                       PVFFIRDGMKFPDMVHALKPNPKSHIQENWRILDFFSHHPESLHMFTFLF   190
Eriobotrya_japonica                      PVFFVRDAMKFPDVIHAFKPNPKSHIQEYWRVLDFLSHHPESLLTFAFLF   190
Gossypium_raimondii                      PVFFIRDGMKFPDMVHALKPNPKSHIQENWRILDFFSHHPESLHMFTFLF   190
Tarenaya_hassleriana_3                   PVFFIRDGMKFPDMVHALKPNPKSHIQENWRILDFFSHHPESLHMFSFLF   190
Jatropha_curcas_2                        PVFFIRDGIKFPDVIHAFKPNPKSHIQEYWRIFDFLSHHPESLSTFAWFF   190
Hevea_brasiliensis                       PVFFIRDGIKFPDMVHALKPNPKSHIQETWRILDFFSHHPESLHMFTFLF   190
Arabidopsis_thaliana_4                   PVFFIRDGMKFPDIVHALKPNPKSHIQENWRILDFFSHHPESLNMFTFLF   190
Elaeis_guineensis                        PVFFVRDGIKFPDMVHSLKPNPKSHIQENWRILDFFSHHPESLHMFTFLF   190
Gossypium_arboreum_2                     PVFFIRDGMKFPDMVHALKPNPKSHIQENWRILDFFSHHPESLHMFTFLF   190
Prunus_mume_2                            PVFFVRDGMKFPDMVHALKPNPKSHIQEPWRILDFFSHHPESLHMFTFLF   190
Citrus_sinensis                          PVFFVRDGMKFPDMVHALKPNPKSHIQENWRIVDFFSHHPESLHMFSFLF   190
Brassica_juncea_4                        PVFFIRDGMKFPDMVHALKPNPKSHIQENWRVLDFFSHHPESLNMFTFLF   190
Consensus                                p ff rd   f       k np           d  s  pesl
```

```
Amaranthus_cruentus_cv._Hopi_Red_Dye     DDVGIPINYRHMNGSGVHTFTLLNKAGKVTYVKFHWRPKCGEKNLL.EEE   239
Bos_taurus                               SDRGIPDGHRHMNGYGSHTFKLVNANGEAVYCKFHYKTDQGIKNLS.VED   249
Homo_sapiens                             SDRGIPDGHRHMNGYGSHTFKLVNANGEAVYCKFHYKTDQGIKNLS.VED   249
Beta_vulgaris_subsp._vulgaris_1          DDVGIPLNYRHMNGSGVHTFTLINKAGKVTYVKFHWRPTCGEKNLL.EEE   239
Mesembryanthemum_crystallinum|root       DDVGIPINYRHMEGFGVHTFTMLNRAGKETYVKFHWKPTCGVKSIVTEEE   240
Mesembryanthemum_crystallinum|leaf       DDVGVPTDYRHMEGSGVNTYTLISKSGKVHYVKFHWKPTCGVKCLL.EEE   239
Prunus_mume_1                            DDLGVPQDYRHMEGSSVHAYTLISKAGKVHYVKFHWKPTCGVKCLL.EDE   239
Gardenia_jasminoides                     DDVGIPQDYRHMEGFGIHAYTLINKAGKAHYVKFHWKPTCGVKSLL.EEE   239
Bassia_scoparia                          DDIGIPANYRHMEGSGVNTYTLVNKAGKAYYVKFHWKPTCGVKSLL.EDE   239
Beta_vulgaris_subsp._vulgaris_2          DDIGVPANYRHMEGSGVNTYTLINKAGKVHYVKFHWKPTCGVKSLL.EDE   239
Suaeda_salsa                             DDIGIPANYRHMEGSGVNTYTLINKAGKTYYVKFHWKPTCGVKSLL.EDE   239
Nicotiana_tabacum_1                      DDIGIPQDYRHMDGSGVHTFTLINKAGKSTYVKFHWKPTCGVKSLL.EEE   239
Nicotiana_tabacum_2                      DDIGIPQDYRHMDGSGVHTFTLINKAGKSTYVKFHWKPTCGVKSLL.EEE   239
Prunus_persica                           DDLGVPQDYRHMEGSSVHAYTLISKAGKVHYVKFHWKPTCGVKCLL.EDE   239
Nicotiana_benthamiana                    DDIGIPQDYRHMDGSGVHTFTLINKAGKSTYVKFHWKPTCGVKSLL.EDE   239
Vitis_vinifera_1                         DDVGIPQDYRHMEGFGVHTFTLLNKAGKANYVKFHWKPTCGVKCLL.EDE   239
Nicotiana_sylvestris                     DDIGIPQDYRHMDGSGVHTFTLINKAGKSTYVKFHWKPTCGVKSLL.EEE   239
Vitis_vinifera_2                         DDVGVPQDYRHMEGFGVHTFTLLNKAGKANYVKFHWKPTCGVKCLL.EDE   239
Prunus_avium                             DDLGIPQDYRHMEGSSVHAYTLISKAGKVHYVKFHWKPTCGVKCLL.EDE   239
Vitis_vinifera_3                         DDVGIPQDYRHMEGFGVHTFTLLNKAGKANYVKFHWKPTCGVKCLL.EDE   239
Beta_vulgaris_subsp._maritima            DDIGVPANYRHMEGSGVNTYTLINKAGKVHYVKFHWKPTCGVKSLL.EDE   239
Nicotiana_tomentosiformis                DDIGIPQDYRHMDGSGVHTFTLINKAGKSTYVKFHWKPTCGVKSLL.EDE   239
Solanum_tuberosum                        DDIGIPQDYRHMDGSGVHTFTLINRAGKSTYVKFHWKPTCGVKSLL.EEE   239
Eucalyptus_grandis_1                     DDVGIPQDYRHMEGFGVHAFTFINKTGKTNYVKFHWKPTCGVKCLL.EEE   239
Solanum_lycopersicum                     DDIGIPQDYRHMDGSGVHTFTLINRAGKSTYVKFHWKPTCGVKSLL.EEE   239
Tarenaya_hassleriana_1                   DDIGIPQDYRHMEGSGVNTYMLVNKAGKAVYVKFHWKPTCGVKSLL.EDE   239
Vitis_vinifera_4                         DDVGIPQDYRHMEGSGVNTYTLINKAGKAHYVKFHWKPTCGVKCLL.EEE   239
Musa_acuminata_subsp._malaccensis        DDVGVPLNYRHMDGSGVHTFTLLNREGKATYVKFHWRPTSGVKCLL.EDE   239
Theobroma_cacao_1                        DDVGVPQDYRHMDGSGVHTYTLINKAGKAQYVKFHWRPTCGVKCLL.DDE   239
Sesamum_indicum_1                        DDIGVPQDYRHMDGSGVNTYTLINKAGKAHYVKFHWKPTCGVKSLL.EDE   239
Brassica_juncea_1                        DDIGIPQDYRHMEGSGVNTYMLINKAGKAHYVKFHWKPTCGVKSLL.EED   239
Hylocereus_undatus                       DDVGIPQDYRHMEGFGVNTFSFINRDGKVHYVKFHWKPTCGVKSLL.EDE   239
Camelina_sativa_1                        DDIGIPQDYRHMDGSGVNTYMLINKAGKAHYVKFHWRPTCGVKSLL.EED   239
Camelina_sativa_2                        DDIGIPQDYRHMDGSGVNTYMLINKAGKAHYVKFHWRPTCGVKSLL.EED   239
Ipomoea_batatas_1                        DDVGIPTDYRHMEGFGVHTFTMINKEGKANYVKFHWKPTCGIKCLL.EEE   239
Raphanus_sativus_1                       DDIGIPQDYRHMEGSGVNTYMLINKSGKAHYVKFHWKPTCGVKSLL.EED   239
Ipomoea_batatas_2                        DDVGIPTDYRHMEGFGVHTFTMINKEGKANYVKFHWKPTCGVKCLL.EEE   239
Camelina_sativa_3                        DDIGIPQDYRHMDGSGVNTYMLINKAGKAHYVKFHWRPTCGVKSLL.EED   239
Rheum_australe                           DDIGIPQDYRHMDGSGVNTYTLVNKVGKVHYVKFHWKPTCGVKSLL.EDE   239
Brassica_juncea_2                        DDIGIPQDYRHMEGSGVNTYMLINKSGKAHYVKFHWKPTCGVKSLL.EED   239
Ziziphus_jujuba                          DDLGVPQDYRHMEGSGVNTYTLINKAGKAHYVKFHWKPTCGVKCLL.EDE   239
Tarenaya_hassleriana_2                   DDIGVPQDYRHMEGSGVNTYMLINKAGKAVYVKFHWKPTCGVKSLL.EEE   239
Brassica_rapa                            DDIGIPQDYRHMEGSGVNTYMLINKSGKAHYVKFHWKPTCGVKSLL.EED   239
Jatropha_curcas_1                        DDIGIPQDYRHMDGSGVNTYTLINKAGKAHYVKFHWKPTCGVKSLL.EDD   239
Nicotiana_glutinosa                      DDIGIPQDYRHMDGSGVHTFTLINKAGKSTYAKFHWKPTCGVQSLL.EDE   239
Brassica_juncea_3                        DDIGIPQDYRHMEGSGVNTYMLINKSGKAHYVKFHWKPTCGVKSLL.EED   239
Arabis_alpina                            DDIGIPQDYRHMDGSGVNTYMLINKAGKAHYVKFHWRPTCGVKSLL.EED   239
Eucalyptus_grandis_2                     DDVGIPQDYRHMEGFGVHAFTFINKAGKTNYVKFHWKPTCGVKCLL.EEE   239
Theobroma_cacao_2                        DDLGVPQDYRHMEGSGVNTYTLINKAGKAQYVKFHWKPTCGVKCLL.EEE   239
Arabidopsis_thaliana_1                   DDIGIPQDYRHMDGSGVNTYMLINKAGKAHYVKFHWKPTCGVKSLL.EED   239
Brassica_oleracea                        DDIGVPQDYRHMEGSGVNTYMLINKSGKAHYVKFHWKPTCGVKSLL.EED   239
Arabidopsis_thaliana_2                   DDIGIPQDYRHMDGSGVNTYMLINKAGKAHYVKFHWKPTCGVKSLL.EED   239
Brassica_napus                           DDIGIPQDYRHMEGSGVNTYMLINKAGKAHYVKFHWKPTCGVKSLL.EED   239
Sesamum_indicum_2                        DDVGVPQDYRHMDGSGVNTYTLINKAGKAHYVKFHWKPTCGVKCLL.EEE   239
Solanum_melongena                        DDVCLPINYRHMEGFGVHAYQLINKAGKAHYVKFHWKPTCGVKSMT.EEE   239
Sesamum_indicum_3                        DDVGVPQDYRHMDGSGVNTYTLINKAGKAHYVKFHWKPTCGVKCLL.EEE   239
Raphanus_sativus_2                       DDIGIPQDYRHMEGSGVNTYMLINKSGKAHYVKFHWKPTCGVKSLL.EED   238
Genlisea_aurea                           DDLGVPQDYRHMDGSGVNTYSLINKAGKAHYVKFHWRPTCGVKCLL.EDE   239
Arabidopsis_thaliana_3                   DDIGIPQDYRHMDGSGVNTYMLINKAGKAHYVKFHWKPTCGVKSLL.EED   239
Gossypium_arboreum_1                     DDLGVPQDYRHMEGSGVNTYTLINKAGKAHYVKFHWKPTCGVKCLL.EDE   239
Gossypium_hirsutum                       DDLGVPQDYRHMEGSGVNTYTLINKAGKAHYVKFHWKPTCGVKCLL.EDE   239
Eriobotrya_japonica                      DDVGVPQDYRHMEGFGVHSYTLISKAGKVHYVKFHWKPTCGVKCLL.EDE   239
Gossypium_raimondii                      DDLGVPQDYRHMEGSGVNTYTLINKAGKAHYVKFHWKPTCGVKCLL.EDE   239
Tarenaya_hassleriana_3                   DDLGVPQDYRHMEGSGVNTYMLINKAGKAHYVKFHWKPTCGVKCLL.EEE   239
Jatropha_curcas_2                        DDVGIPQDYRHMEGFGVHTFCFLNKAGKVTYVKFHWKPTCGVKCLT.DDE   239
Hevea_brasiliensis                       DDIGIPQDYRHMDGSGINTYTLINKAGKAYYVNFHWKPTCGVKSLL.EDE   239
Arabidopsis_thaliana_4                   DDIGIPQDYRHMDGSGVNTYMLINKAGKAHYVKFHWKPTCGVKSLL.EED   239
Elaeis_guineensis                        DDVGVPADYRHMDGSGVNTYTLINKEGKAHYVKFHWRPTCGVKCLL.EDE   239
Gossypium_arboreum_2                     DDIGVPQDYRHMDGSGVHTYTLINKAGKSHYVKFHWKPTCGVKSLL.EDE   239
Prunus_mume_2                            DDLGVPQDYRHMEGSGVNTYTLINKAGKAQYVKFHWKPTCGVKCLL.EDE   239
Citrus_sinensis                          DDVGVPRDYRHMEGSGVNTYMLINKAGKAHYVKFHWKPTCGVKCLL.EDE   239
Brassica_juncea_4                        DDIGIPQDYRHMEGSGVNTYMLINKSGKAHYVKFHWKPTCGVKSLL.EED   239
Consensus                                 d   p   rhm g            g   y  fh     g
```

```
Amaranthus_cruentus_cv._Hopi_Red_Dye    AIRIGGENHSHATQDLYESIAAGNFPEWTLYFQTMDPADEDKFDFDPLDT   289
Bos_taurus                              AARLAHEDPDYGLRDLFNAIATGNYPSWTLYIQVMTFSEAEIFPFNPFDL   299
Homo_sapiens                            AARLSQEDPDYGIRDLFNAIATGKYPSWTFYIQVMTFNQAETFPFNPFDL   299
Beta_vulgaris_subsp._vulgaris_1         AIRVGGENHSHATQDLYESIASGNFPEWKLYIQTMDPNDEDKFDFDPLDM   289
Mesembryanthemum_crystallinum|root      AIQVGGANHSHATQDLYDSIAAGNYPEWRLFIQTMDPADEDKFDFDPLDI   290
Mesembryanthemum_crystallinum|leaf      AVKIGGANHSHATQDLYDSIAAGNYPEWKLYIQTIDPADEDKFDFDPLDV   289
Prunus_mume_1                           AIKVGGANHSHATKDLYDSIAAGNYPEWKLYIQTMDPDHEDRFDFDPLDL   289
Gardenia_jasminoides                    AIRVGGSNHSHATQDLYDSIAAGNYPEWKLFIQTMDPDHEDQFDFDPLDV   289
Bassia_scoparia                         AIKIGGANHSHATQDLYDSIAAGNYPEWKLFIQTIDPADEDKFDFDPLDV   289
Beta_vulgaris_subsp._vulgaris_2         AIKVGGANHSHATQDLYDSIAAGNYPEWKLFIQIIDQADEDKFDFDPLDV   289
Suaeda_salsa                            AIKVGGANHSHATQDLYDSIASGNYPEWKLFIQTIDPADEDKFDFDPLDV   289
Nicotiana_tabacum_1                     AARIGGANHSHATQDLYDSIAAGNYPEWKLFIQTMDPDHEDRFDFDPLDV   289
Nicotiana_tabacum_2                     AARIGGANHSHATQDLYDSIAAGNYPEWKLFIQTMDPDHEDRFDFDPLDV   289
Prunus_persica                          AIKVGGANHSHATKDLYDSIAAGNYPEWKLYIQTMDPDHEDRFDFDPLDL   289
Nicotiana_benthamiana                   AARVGGANHSHATQDLYDSIAAGNYPEWKLFIQTMDPDHEDRFDFDPLDV   289
Vitis_vinifera_1                        AIRVGGTNHSHATQDLYDSIKAGNYPEWKLFIQTMDPEHEDKFDFDPLDV   289
Nicotiana_sylvestris                    AARIGGANHSHATQDLYDSIAAGNYPEWKLFIQTMDPDHEDRFDFDPLDV   289
Vitis_vinifera_2                        AIRVGGTNHSHATQDLYDSIKAGNYPEWKLFIQTMDPEHEDKFDFDPLDV   289
Prunus_avium                            AIKVGGANHSHATKDLYDSIAAGNYPEWKLYIQTMDPDHEDRFDFDPLDL   289
Vitis_vinifera_3                        AIRVGGTNHSHATQDLYDSIKAGNYPEWKLFIQTMDPEHEDKFDFDPLDV   289
Beta_vulgaris_subsp._maritima           AIKVGGANHSHATQDLYDSIAAGNYPEWKLFIQIIDQADEDKFDFDPLDV   289
Nicotiana_tomentosiformis               AARVGGANHSHATQDLYDSIAAGNYPEWKLFIQTMDPDHEDRFDFDPLDV   289
Solanum_tuberosum                       AIRVGGANHSHATQDLYDSIAAGNYPEWKLFIQIMDPDHEDKFDFDPLDV   289
Eucalyptus_grandis_1                    AIRIGGSNHSHATKDLYDSIAAGNYPEWKLYIQVMDPALEDSFDFDPLDM   289
Solanum_lycopersicum                    AIRVGGANHSHATQDLYDSIAAGNYPEWKLFIQIMDPEHEDKFDFDPLDV   289
Tarenaya_hassleriana_1                  AIRVGGSNHSHATQDLYDSIAAGNYPEWKLFIQTIDPADEDKFDFDPVDV   289
Vitis_vinifera_4                        AIRVGGSNHSHATQDLYDSIAAGNYPEWKLFIQTIDPDHEDKFDFDPLDV   289
Musa_acuminata_subsp._malaccensis       AVTVGGNNHSHGTQDLYDSIAAGNYPEWKLFIQTMDPEHEDRFDFDPLDV   289
Theobroma_cacao_1                       AIRVGGSNHSHATQDLYDSIAAGNYPEWKLFIQTMDPAHEDRFDFDPLDV   289
Sesamum_indicum_1                       AIKVGGANHSHATQDLYDSIAAGNYPEWKLFIQIIDPDHEDRFDFDPLDV   289
Brassica_juncea_1                       AIRVGGTNHSHATQDLYDSIAAGNYPEWKLFVQIIDPADEDKFDFDPLDV   289
Hylocereus_undatus                      AVRVGGLNHSHATQDLYESIAAGNYPEWKLCIQIMDPADEDKYDFDPLDM   289
Camelina_sativa_1                       AIRVGGTNHSHATQDLYDSIAAGNYPEWKLFIQTIDPADEDKFDFDPLDV   289
Camelina_sativa_2                       AIRIGGTNHSHATQDLYDSIAAGNYPEWKLFIQIIDPADEDKFDFDPLDV   289
Ipomoea_batatas_1                       AIRIGGENHSHATQDLYESIAAGNYPEWKLYIQVMDPDHEDRFDFDPLDT   289
Raphanus_sativus_1                      AIRVGGTNHSHATQDLYDSIAAGNYPEWKLFVQIIDPADEDKFDFDPLDV   289
Ipomoea_batatas_2                       AIRIGGENHSHATQDLYESIAAGNYPEWKLYIQVMDPDHEDRFDFDPLDT   289
Camelina_sativa_3                       AIRIGGTNHSHATQDLYDSIAAGNYPEWKLFIQIIDPADEDKFDFDPLDV   289
Rheum_australe                          AIKVGGANHSHATQDLYDSIAAGNYPEWKLFIQVIDPVDEDKFDFDPLDV   289
Brassica_juncea_2                       AVRVGGTNHSHATQDLYDSIAAGNYPEWKLFIQVIDPADEDKFDFDPLDV   289
Ziziphus_jujuba                         AIKVGGANHSHATQDLYDSIAAGNYPEWKLFIQTIDPDYEDRFDFDPLDV   289
Tarenaya_hassleriana_2                  AIRVGGSNHSHATQDLYDSIAAGNYPEWKLFIQTIDPADEDKFDFDPVDV   289
Brassica_rapa                           AVRVGGTNHSHATQDLYDSIAAGNYPEWKLFIQTMDPADEDKFDFDPLDV   289
Jatropha_curcas_1                       AIRVGGANHSHATQDLYDSIAAGNYPEWKLFIQIIDPLDEDKFDFDPLDV   289
Nicotiana_glutinosa                     AARVGGANHSHATQDLYDSIAAGNYPEWKLFIQTMDPSHEDRFDFDPLDV   289
Brassica_juncea_3                       AIRVGGTNHSHATQDLYDSIAAGNYPEWKLFVQIIDPADEDRFDFDPLDV   289
Arabis_alpina                           AIRVGGTNHSHATQDLYDSIAAGNYPEWKLFIQIIDPADEDKFDFDPLDV   289
Eucalyptus_grandis_2                    AIRIGGSNHSHATKDLYDSIAAGNYPEWKLYIQVMDPALEDSFDFDPLDM   289
Theobroma_cacao_2                       AIKVGGANHSHATQDLYDSIAAGNYPEWKLFIQTMDPDHEDRFDFDPLDV   289
Arabidopsis_thaliana_1                  AIRVGGTNHSHATQDLYDSIAAGNYPEWKLFIQIIDPADEDKFDFDPLDV   289
Brassica_oleracea                       AIRVGGTNHSHATQDLYDSIAAGNYPEWKLFVQIIDPADEDKFDFDPLDV   289
Arabidopsis_thaliana_2                  AIRVGGTNHSHATQDLYDSIAAGNYPEWKLFIQIIDPADEDKFDFDPLDV   289
Brassica_napus                          AIRVGGTNHSHATQDLYDSIAAGNYPEWKLFVQIIDPADEDKFDFDPLDV   289
Sesamum_indicum_2                       AIKVGGANHSHATQDLYDSIAAGNYPEWKLFIQIMDPDHEDRFDFDPLDV   289
Solanum_melongena                       AIRVGGTNHSHATKDLYDSIAAGNYPEWKLFIQIMDPEDVDKFDFDPLDV   289
Sesamum_indicum_3                       AIKVGGANHSHATQDLYDSIAAGNYPEWKLFIQIMDPDHEDRFDFDPLDV   289
Raphanus_sativus_2                      AIRVGGTNHSHATQDLYDSIAAGNYPEWKLFVQIIDPADEDKFDFDPLDV   288
Genlisea_aurea                          AIKVGGANHSHATQDLYDSIAAGNFPEWKLFIQTIDPDHEDRFDFDPLDV   289
Arabidopsis_thaliana_3                  AILVGGTNHSHATQDLYDSIAAGNYPEWKLFIQIIDPADEDKFDFDPLDV   289
Gossypium_arboreum_1                    AIKVGGANHSHATQDLYDSIAAGNYPEWKLFIQTIDPDHEDKFDFDPLDV   289
Gossypium_hirsutum                      AIKVGGANHSHATQDLYDSIAAGNYPEWKLFIQTIDPDHEDKFDFDPLDV   289
Eriobotrya_japonica                     AIKVGGANHSHATQDLYDSIAAGNYPEWKLFIQTMDPDHEDRFDFDPLDD   289
Gossypium_raimondii                     AIKVGGANHSHATQDLYDSIAAGNYPEWKLFIQTIDPDHEDKFDFDPLDV   289
Tarenaya_hassleriana_3                  AIRVGGSNHSHATQDLYDSIAAGNYPEWKLFIQIMDPVDEDKFDFDPLDV   289
Jatropha_curcas_2                       AMKIGGANHSHATQDLYDSIAAGNYPEWILHIQTMDPADEDRFDFDPLDM   289
Hevea_brasiliensis                      AIRVGGSNHSHATQDLYDSIAAGNYPEWKLFIQTIDPADEEKFDFDPLDV   289
Arabidopsis_thaliana_4                  AIRLGGTNHSHATQDLYDSIAAGNYPEWKLFIQIIDPADEDKFDFDPLDV   289
Elaeis_guineensis                       AVIVGGNNHSHATKDLYDSIAAGNYPEWKLYIQTIDPDHEDKFDFDPLDV   289
Gossypium_arboreum_2                    AIRVGGANHSHATQDLYDSIAAGNYPEWKLFIQIMDPLHEDRFDFDPLDV   289
Prunus_mume_2                           AIKVGGANHSHATKDLYDSIAAGNYPEWKLYIQTIDPDHEDRFDFDPLDV   289
Citrus_sinensis                         AIKVGGANHSHATQDLYDSIASGNYPEWKLYIQIMDPDHEDKFDFDPLDV   289
Brassica_juncea_4                       AVRVGGTNHSHATQDLYDSIAAGNYPEWKLFIQVIDPADEDKFDFDPLDV   289
Consensus                               a             dl   i  g  p w    q           f p d
```

```
Amaranthus_cruentus_cv._Hopi_Red_Dye      TKTWPENIFPLQPVGRLVLNRNVDNFFTENEQLAFDPAHIVPGIYFSNDK   339
Bos_taurus                                TKVWPHGDYPLIPVGKLVLNRNPVNYFAEVEQLAFDPSNMPPGIEPSPDK   349
Homo_sapiens                              TKVWPHKDYPLIPVGKLVLNRNPVNYFAEVEQIAFDPSNMPPGIEASPDK   349
Beta_vulgaris_subsp._vulgaris_1           TKTWPEDILPLQPVGRLVLNKNVDNFFTENEQLAFDPAHVVPGIYFSNDK   339
Mesembryanthemum_crystallinum|root        TKTWPEDILPLQPVGRLVLNKNIDNFFTENEMLAFNPASIVPGIHFSHDK   340
Mesembryanthemum_crystallinum|leaf        TKTWPEDILPLQPVGRLVLNKNIDNFFAENEQLAFCPGIVVPGVYYSDDK   339
Prunus_mume_1                             TKTWPEDILPLQPVGRLVLNKNIDNFFAENEQLAFNPAHVVPGVYYSDDK   339
Gardenia_jasminoides                      TKIWPEDILPLQPVGRLVLNKNIDNFFNENEQLAFNPAHVVPGIYYSEDK   339
Bassia_scoparia                           TKTWPEDILPLQPVGRLVLNKNIDNFFAENEQLAFCPAIVVPGIHYSDDK   339
Beta_vulgaris_subsp._vulgaris_2           TKTWPEDILPLLPVGRMVLNKNIDNFFAENEQLAFCPAIVVPGVSYSDDK   339
Suaeda_salsa                              TKTWPEDILPLQPVGRLVLNKNIDNFFAENEQLAFCPSIVVPGVHYSDDK   339
Nicotiana_tabacum_1                       TKTWPEDILPLQPVGRLVLNKNIDNFFNENEQLAFCPSIVVPGVYYSDDK   339
Nicotiana_tabacum_2                       TKTWPEDILPLQPVGRLVLNKNIDNFFNENEQLAFCPSIVVPGVYYSDDK   339
Prunus_persica                            TKTWPEDILPLQPVGRLVLNKNIDNFFAENEQLAFNPAHVVPGIYYSDDK   339
Nicotiana_benthamiana                     TKTWPEDILPLQPVGRLVLNKNIDNFFNENEQLAFCPSIVVPGVYYSDDK   339
Vitis_vinifera_1                          TKTWPEDILPLQPVGRLVLNKNIDNFFAENEMLAFNPALVVPGIYYTNDK   339
Nicotiana_sylvestris                      TKTWPEDILPLQPVGRLVLNKNIDNFFNENEQLAFCPSIVVPGVYYSDDK   339
Vitis_vinifera_2                          TKTWPEDILPLQPVGRLVLNKNIDNFFAENEMLAFNPALVVPGIYYTNDK   339
Prunus_avium                              TKTWPEDILPLQPVGRLVLNKNIDNFFAENEQLAFNPAFVVPGIYYSDDK   339
Vitis_vinifera_3                          TKTWPEDILPLQPVGRLVLNKNIDSFFAENEMLAFNPALVVPGIYYTNDK   339
Beta_vulgaris_subsp._maritima             TKTWPEDILPLLPVGRMVLNKNIDNFFAENEQLAFCPAIVVPGVSYSDDK   339
Nicotiana_tomentosiformis                 TKTWPEDILPLQPVGRLVLNKNIDNFFNENEQLAFCPSIVVPGVYYSDDK   339
Solanum_tuberosum                         TKTWPEDILPLQPVGRLVLNKNIDNFFNENEQLAFCPSIVVPGVYYSDDK   339
Eucalyptus_grandis_1                      TKEWPEDILPLQPVGRLVLNKNVDNFFAENEQLAFNPAFVVPGIYYSNDK   339
Solanum_lycopersicum                      TKTWPEDILPLQPVGRLVLNKNIDNFFNENEQLAFCPSIVVPGVYYSDDK   339
Tarenaya_hassleriana_1                    TKTWPEDILPLQPVGRLVLNKNIDNFFAENEQLAFCPAIVVPGVYYSDDK   339
Vitis_vinifera_4                          TKTWPEDILPLQPVGRLVLNRNIDNFFAENEQLAFCPAIVVPGVYYSDDK   339
Musa_acuminata_subsp._malaccensis         TKTWPEDIFPLQPVGRMVLNKNIDNFFNENEQLAFCPAIIVPGIYYSDDK   339
Theobroma_cacao_1                         TKTWPEDIFPLQPVGRLVLNKNIDNFFAENEQLAFCPSIIVPGLYYSDDK   339
Sesamum_indicum_1                         TKTWPEDILPLQPVGRMVLNKNIDNFFAENEQLAFCPAIIVPGIYYSDDK   339
Brassica_juncea_1                         TKTWPEDILPLQPVGRMVLNKNIDNFFAENEQLAFCPAIIVPGIHYSDDK   339
Hylocereus_undatus                        TKIWPEDLVPLQPVGRLVLNRNVDNFFAENEQLAFSPGVVVPGIYYSDDK   339
Camelina_sativa_1                         TKTWPEDILPLQPVGRMVLNKNIDNFFAENEQLAFCPAIIVPGIHYSDDK   339
Camelina_sativa_2                         TKTWPEDILPLQPVGRMVLNKNIDNFFAENEQLAFCPAIIVPGIHYSDDK   339
Ipomoea_batatas_1                         TKIWPEELIPLQPVGRMVLNKNIDNFFAENEMLAMDPAHIVPGIYFSDDK   339
Raphanus_sativus_1                        TKTWPEDLLPLQPVGRMVLNKNIDNFFAENEQLAFCPAIIVPGIHYSDDK   339
Ipomoea_batatas_2                         TKIWPEELIPLQPVGRMVLNKNIDNFFAENEMLAMDPAHIVPGIYFSDDK   339
Camelina_sativa_3                         TKTWPEDILPLQPVGRMVLNKNIDNFFAENEQLAFCPAIIVPGIHYSDDK   339
Rheum_australe                            TRTWPEDILPLIPVGRMVLNKNIDNFFAENEQLAFCPGIVVPGIYYSDDK   339
Brassica_juncea_2                         TKTWPENLLPLQPVGRMVLNKNIDNFFAENEQLAFCPAIIVPGIHYSDDK   339
Ziziphus_jujuba                           TKTWPEDILPLQPVGRLVLNKNIDNFFAENEQLAFCPAIVVPGVYYSDDK   339
Tarenaya_hassleriana_2                    TKTWPEDIVPLQPVGRLVLNKNIDSFFAENEQLAFCPGIIVPGIHYSDDK   339
Brassica_rapa                             TKTWPEELLPLQPVGRMVLNKNIDNFLAENEQLAFCPAIIVPGIYYSDDK   339
Jatropha_curcas_1                         TKTWPEDILPLQPVGRMVLNRNIDNFFAENEQLAFCPAIIVPGVYYSDDK   339
Nicotiana_glutinosa                       TKTWPEDILPLQPVGRLVLNKNIDNFFNENEQLAFCPSIVVPGVYYSDDK   339
Brassica_juncea_3                         TKTWPEDILPLQPVGRMVLNKNIDNFFAENEQLAFCPAIIVPGIHYSDDK   339
Arabis_alpina                             TKTWPEDILPLQPVGRMVLNKNIDNFFAENEQLAFCPAIIVPGIHYSDDK   339
Eucalyptus_grandis_2                      TKIWPEDILPLQPVGRLVLNQNVDNFFAENEQLAFNPAFVVPGIYFSNDK   339
Theobroma_cacao_2                         TKTWPEDILPLQPVGRLVLNKNIDNFFAENEQLAFCPAIVVPGIYYSDDK   339
Arabidopsis_thaliana_1                    TKTWPEDILPLQPVGRMVLNKNIDNFFAENEQLAFCPAIIVPGIHYSDDK   339
Brassica_oleracea                         TKTWPEDLLPLQPVGRMVLNKNIDNFFAENEQLAFCPAIIVPGIHYSDDK   339
Arabidopsis_thaliana_2                    TKTWPEDILPLQPVGRMVLNKNIDNFFAENEQLAFCPAIIVPGIHYSDDK   339
Brassica_napus                            TKTWPEDILPLQPVGRMVLNKNIDNFFAENEQLAFCPAIIVPGIHYSDDK   339
Sesamum_indicum_2                         TKTWPEDILPLQPVGRLVLNKNIDNFFAENEQLAFCPAIIVPGIYYSDDK   339
Solanum_melongena                         TKTWPEDILPLMPVGRLVLNRNIDNFFAENEQLAFNPGHIVPGVYYSEDK   339
Sesamum_indicum_3                         TKTWPEDILPLQPVGRLVLNKNIDNFFAENEQLAFCPAIIVPGIYYSDDK   339
Raphanus_sativus_2                        TKTWPEDLLPLQPVGRMVLNKNIDNFFAENEQLAFCPAIIVPGIHYSDDK   338
Genlisea_aurea                            TKTWPEDVLPLQPVGRLVLNRNIDNFFAENEQLAFCPAIIVPGIYYSDDK   339
Arabidopsis_thaliana_3                    TKTWPEDILPLQPVGRMVLNKNIDNFFAENEQLAFCPAIIVPGIHYSDDK   339
Gossypium_arboreum_1                      TKTWPEDILPLQPVGRLVLNKNIDNFFAENEQLAFCPAIVVPGIYYSDDK   339
Gossypium_hirsutum                        TKTWPEDILPLQPVGRLVLNKNIDNFFAENEQLAFCPAIVVPGIYYSDDK   339
Eriobotrya_japonica                       TKTWPEDILPLQPVGRLVLNKNIDNFFAENEQLAFNPAFVVPGVYYSNDK   339
Gossypium_raimondii                       TKTWPEDILPLQPVGRLVLNKNIDNFFAENEQLAFCPAIVVPGIYYSDDK   339
Tarenaya_hassleriana_3                    TKTWPEDILPLQPVGRLVLNKNIDNFFAENEQVAFCPALVVPGVYYSDDK   339
Jatropha_curcas_2                         TKIWPEDIFPLQPIGRLVLNRNIDNWFAENEMLAFNPAHVVPGIHYSNDK   339
Hevea_brasiliensis                        TKTWPEDILPLQPVGRLVLNRNIDNFFAENEQLAFCPSIVVPGVYYSDDK   339
Arabidopsis_thaliana_4                    TKTWPEDILPLQPVGRMVLNKNIDNFFAENEQLAFCPAIIVPGIHYSDDK   339
Elaeis_guineensis                         TKTWPEDILPLQPVGRMVLNKNIDNFFAENEQLAFCPAIVVPGIYYSDDK   339
Gossypium_arboreum_2                      TKTWPEDIFPLQPVGRMVLNKNIDNFFAENEQLAFCPSLIVPGIYYSDDK   339
Prunus_mume_2                             TKTWPEDILPLQPVGRLVLNKNIDNFFAENEQLAFCPAIVVPGVYYSDDK   339
Citrus_sinensis                           TKTWPEDILPLQPVGRLVLNKNIDNFFAENEQLAFCPAIVVPGIYYSNDK   339
Brassica_juncea_4                         TKTWPENLLPLQPVGRMVLNKNIDNFFAENEQLAFCPAIIVPGIHYSDDK   339
Consensus                                 t  wp    pl p g  vln n      e e  a  p    pg     dk
```

```
Amaranthus_cruentus_cv._Hopi_Red_Dye  MLQARTFAYADTHRYRLGINYLQLPVNAP.KCPHTNNHRDGAMTMIHRDE   388
Bos_taurus                            MLQGRLFAYPDTHRHRLGPNYLQIPVNCPYRARVANYQRDGPMCMMDNQG   399
Homo_sapiens                          MLQGRLFAYPDTHRHRLGPNYLHIPVNCPYRARVANYQRDGPMCMQDNQG   399
Beta_vulgaris_subsp._vulgaris_1       MLQARTFAYADTHRYRLGINYLQLPVNAP.KCPHTNMHRDGAMNMVHRDE   388
Mesembryanthemum_crystallinum|root    MLQARTFAYADTQRHRLGVNFLLLPVNAP.KCAHHNDHHDGFMNFMHRDE   389
Mesembryanthemum_crystallinum|leaf    LLQTRIFSYADTQRHRLGPNYLQLPVNAP.KCAHHNNHYDGQMNFMHRDD   388
Prunus_mume_1                         MLQTRIFAYSDTQRHRLGPNYLQLPVNAP.KCPHHNNHHEGFMNFMHRDE   388
Gardenia_jasminoides                  LLQGRIFAYSDTHRHRLGPNYLQLPVNAP.KCAHHNNHYDGYMNIMHRDE   388
Bassia_scoparia                       LLQTRIFSYSDTQRHRLGPNYLQLPVNAP.KCSHHNNHYDGFMNFMHRGE   388
Beta_vulgaris_subsp._vulgaris_2       LLQTRIFSYSDTQRHRLGPNYLQLPVNAP.KCSHHNNHYDGFMNFMHRDE   388
Suaeda_salsa                          LLQTRIFSYSDTQRHRLGPNYLQLPVNAP.KCSHHNNHYDGFMNFMHRDE   388
Nicotiana_tabacum_1                   MLQTRIFSYSDTQRYRLGPNYLQLPANAP.KCAHHNNHYDGSMNFMHRDE   388
Nicotiana_tabacum_2                   MLQTRIFSYSDTQRYRLGPNYLQLPANAP.KCAHHNNHYDGSMNFMHRDE   388
Prunus_persica                        VLQTRIFAYSDTQRHRLGPNYLQLPVNAP.KCPHHNNHHEGFMNFMHRDE   388
Nicotiana_benthamiana                 MLQTRIFSYSDTQRYRLGPNYLQLPANAP.KCAHHNNHYDGSMNFMHRDE   388
Vitis_vinifera_1                      MLQGRIFAYSDTQRHRLGPNYLQLPVNAP.KCAFHNNHHDGSMNFMHRDE   388
Nicotiana_sylvestris                  MLQTRIFSYSDTQRYRLGPNYLQLPANAP.KCAHHNNHYDGSMNFMHRDE   388
Vitis_vinifera_2                      MLQGRIFAYSDTQRHRLGPNYLQLPVNAP.KCAFHNNHHDGSMNFMHRDE   388
Prunus_avium                          LLQTRIFAYSDTQRHRLGPNYMQLPVNAP.KCPHHNNHQDGFMNFMHRDE   388
Vitis_vinifera_3                      MLQGRIFAYSDTQRHRLGPNYLQLPVNAP.KCAFHNNHHDGSMNFMHRDE   388
Beta_vulgaris_subsp._maritima         LLQTRIFSYSDTQRHRLGPNYLQLPVNAP.KCSHHNNHYDGFMNFMHRDE   388
Nicotiana_tomentosiformis             MLQTRIFSYSDTQRYRLGPNYLQLPANAP.KCAHHNNHYDGSMNFMHRDE   388
Solanum_tuberosum                     MLQTRIFSYSDTQRYRLGPNYLQLPANAP.KCAHHNNHYDGSMNFMHRDE   388
Eucalyptus_grandis_1                  LLQARIFAYSDTHRYRLGPNYLQLPVNAP.KCAHHNNHHDGFMNIMHRDE   388
Solanum_lycopersicum                  MLQTRIFSYSDTQRYRLGPNYLQLPANAP.KCAHHNNHYDGSMNFMHRDE   388
Tarenaya_hassleriana_1                LLQTRIFSYSDTQRHRLGPNYLQLPVNAP.KCAHHNNHHEGFMNFMHRDE   388
Vitis_vinifera_4                      LLQTRIFSYSDTQRHRLGPNYLQLPANAP.KCAHHNNHHEGFMNFMHRDE   388
Musa_acuminata_subsp._malaccensis     LLQTRIFSYSDTQRHRLGPNYLMLPVNAP.KCAHHNNHHDGFMNFMHRDE   388
Theobroma_cacao_1                     LLQTRIFSYSDTQRHRLGPNYLQLPANAP.KCAHHNNHHEGFMNFMHRDE   388
Sesamum_indicum_1                     LLQTRIFSYADTQRHRLGPNYLQLPANAP.KCAHHNNHHEGFMNFMHRDE   388
Brassica_juncea_1                     LLQTRVFSYADTQRHRLGPNYLQLPVNAP.KCAHHNNHHEGFMNFMHRDE   388
Hylocereus_undatus                    LLQCRVFAYGDAQRYRLGANYMMLPVNAP.KCAHHNNQYDGVMNFMHRDE   388
Camelina_sativa_1                     LLQTRVFSYADTQRHRLGPNYLQLPVNAP.KCAHHNNHHDGFMNFMHRDE   388
Camelina_sativa_2                     LLQTRVFSYADTQRHRLGPNYLQLPVNAP.KCAHHNNHHDGFMNFMHRDE   388
Ipomoea_batatas_1                     MLQARVFAYADTHRHRLGPNYMLLPVNAP.KCAHHNNSYDGYMNFVHRDE   388
Raphanus_sativus_1                    LLQTRVFSYADTQRHRLGPNYLQLPVNAP.KCAHHNNHHEGFMNFMHRDE   388
Ipomoea_batatas_2                     MLQARVFAYADTHRHRLGPNYMLLPVNAP.KCAHHNNSYDGYMNFVHRDE   388
Camelina_sativa_3                     LLQTRVFSYADTQRHRLGPNYLQLPVNAP.KCAHHNNHHDGFMNFMHRDE   388
Rheum_australe                        LLQTRIFSYSDTQRHRLGPNYLQLPVNAP.KCAHHNNHHEGLMNFMHRDE   388
Brassica_juncea_2                     LLQTRVFSYADTQRHRLGPNYLQLPVNAP.KCAHHNNHHEGFMNFMHRDE   388
Ziziphus_jujuba                       LLQTRIFSYSDTQRHRLGPNYLQLPANAP.KCAHHNNHHEGFMNFMHRDE   388
Tarenaya_hassleriana_2                LLQTRVFSYSDTQRHRLGPNYLQLPVNAP.KCAHHNNHHEGFMNFMHRDE   388
Brassica_rapa                         LLQTRVFSYADTQRHRLGPNYLQLPVNSP.KCSHHNNHHEGFMNFMHRDE   388
Jatropha_curcas_1                     LLQTRIFSYSDTQRHRLGPNYLQLPPNAP.KCAHHNNHHEGFMNFMHRDE   388
Nicotiana_glutinosa                   MLQTRIFSYSDTQRYRLGPNYLQLPANAP.KCAHHNNHYDGSMNFMHRDE   388
Brassica_juncea_3                     LLQTRVFSYADTQRHRLGPNYLQLPVNAP.KCAHHNNHHEGFMNFMHRDE   388
Arabis_alpina                         LLQTRVFSYADTQRHRLGPNYLQLPVNAP.KCAHHNNHHEGFMNFMHRDE   388
Eucalyptus_grandis_2                  LLQARIFAYSDTHRYRLGPNYLQLPVNAP.KCAHHNNHHDGFMNFMHRDE   388
Theobroma_cacao_2                     LLQTRIFSYADTQRHRLGPNYLQLPPNAP.KCAHHNNHHEGFMNFMHRDE   388
Arabidopsis_thaliana_1                LLQTRVFSYADTQRHRLGPNYLQLPVNAP.KCAHHNNHHEGFMNFMHRDE   388
Brassica_oleracea                     LLQTRVFSYADTQRHRLGPNYLQLPVNAP.KCAHHNNHHEGFMNFMHRDE   388
Arabidopsis_thaliana_2                LLQTRVFSYADTQRHRLGPNYLQLPVNAP.KCAHHNNHHEGFMNFMHRDE   388
Brassica_napus                        LLQTRVFSYADTQRHRLGPNYLQLPVNAP.KCAHHNNHHEGFMNFMHRDE   388
Sesamum_indicum_2                     LLQTRIFSYADTQRHRLGPNYLQLPVNAP.KCAHHNNHHEGFMNFMHRDE   388
Solanum_melongena                     LLQTRIFAYADTQRHRIGPNYMQLPVNAP.KCAHHNNHRDGAMNFMHRDE   388
Sesamum_indicum_3                     LLQTRIFSYADTQRHRLGPNYLQLPVNAP.KCAHHNNHHEGFMNFMHRDE   388
Raphanus_sativus_2                    LLQTRVFSYADTQRHRLGPNYLQLPVNAP.KCAHHNNHHEGFMNFMHRDE   387
Genlisea_aurea                        LLQTRIFSYADTQRHRLGPNYLQLPVNAP.KNAHHNNHHEGFMNFMHRDE   388
Arabidopsis_thaliana_3                LLQTRVFSYADTQRHRLGPNYLQLPVNAP.KCAHHNNHHEGFMNFMHRDE   388
Gossypium_arboreum_1                  LLQTRIFSYADTQRHRLGPNYLQLPANAP.KCAHHNNHHEGFMNFMHRDE   388
Gossypium_hirsutum                    LLQTRIFSYADTQRHRLGPNYLQLPANAP.KCAHHNNHHEGFMNFMHRDE   388
Eriobotrya_japonica                   MLQTRLFAYSDTQRHRLGPNYLQLPVNAP.KCAHHNNHYDGFMNFMHRDE   388
Gossypium_raimondii                   LLQTRIFSYSDTQRHRLGPNYLQLPANAP.KCAHHNNHHEGFMNFMHRDE   388
Tarenaya_hassleriana_3                LLQTRIFSYADTQRHRLGPNYLQLPVNAP.KCVHHNNHHDGFMNFMHRDE   388
Jatropha_curcas_2                     LFQLRTFAYSDTQRHRLGPNYMQLPVNAP.KCPHHNNHYDGFMNFMHRDE   388
Hevea_brasiliensis                    LLQTRIFSYSDTQRYRLGPNYLQLPANAP.KCAHHNNHHDGFMNFMHRDE   388
Arabidopsis_thaliana_4                LLQTRVFSYADTQRHRLGPNYLQLPVNAP.KCAHHNNHHEGFMNFMHRDE   388
Elaeis_guineensis                     LLQTRIFSYADTQRHRLGPNYLMLPANAP.KCAHHNNHHDGFMNFMHRDE   388
Gossypium_arboreum_2                  LLQTRIFSYSDTQRHRLGPNYLQLPANAP.KCAHHNNHHEGFMNFMHRDE   388
Prunus_mume_2                         LLQTRIFSYSDTQRHRLGPNYLQLPVNAP.KCSHHNNHHEGFMNFMHRDE   388
Citrus_sinensis                       LLQTRIFSYADTQRHRLGPNYLQLPVNAP.KCAQHNNHYDGFMNFMHRDE   388
Brassica_juncea_4                     LLQTRVFSYADTQRHRLGPNYLQLPVNAP.KCAHHNNHHEGFMNFMHRDE   388
Consensus                               q r f y d  r r g n    p n p      n    g m
```

```
Amaranthus_cruentus_cv._Hopi_Red_Dye      .EIDYFPSRFDPVREAEKSPIPTVIVHGRREKQIIPKENNFKEPGERYRS   437
Bos_taurus                                GAPNYYPNSFSAPEHQPSALEHRTHFSGDVQRFNSANDDNVTQVRTFYLK   449
Homo_sapiens                              GAPNYYPNSFGAPEQQPSALEHSIQYSGEVRRFNTANDDNVTQVRAFYVN   449
Beta_vulgaris_subsp._vulgaris_1           .EIDYFPSRFDPLREAERFPIPTAIIHGRREKQIIPKENNFKEPGVRYRS   437
Mesembryanthemum_crystallinum|root        .EVNYFPSRSNVCHHAERFPIPSHTLTGRRERRIIPKENNFKQPGDRYRS   438
Mesembryanthemum_crystallinum|leaf        .EVDYFPSRYDPVRHAERYPIPPNVLNGNRDRQIIQKENNFKQPGERYRS   437
Prunus_mume_1                             .EVNYFPSRYDPVRHAERYPIPSNILYGKREKCVIEKENNFKQPGERYRS   437
Gardenia_jasminoides                      .EIDYFPSRYDPVRHAERHPIPTRVLTGRRERRVIEKENNFKQPVERYRS   437
Bassia_scoparia                           .EVDYFPSRYDPVRHAEKFPIPSNICTGRRERQIIPKENNFKQPGERYRS   437
Beta_vulgaris_subsp._vulgaris_2           .EVDYFPSRYDPVRHAERHPIPSNVINGKRERQIIAKENNFKQPGERYRS   437
Suaeda_salsa                              .EVDYFPSRYDPVRHAERYPIPSNVLNGCREKQCIPKENNFKQPGERYRS   437
Nicotiana_tabacum_1                       .EIDYFPSRYDPVRHAEKYPIPSTMCTGKREKCVIQKENNFKQPGDRYRS   437
Nicotiana_tabacum_2                       .EIDYFPSRYDPVRHAEKYPIPSTMCTGKREKCVIQKENNFKQPGDRYRS   437
Prunus_persica                            .EVNYFPSRHDPVRHAERYPIPSNILSGKREKCVIEKENNFKQPGERYRS   437
Nicotiana_benthamiana                     .EIDYFPSRYDPVRHAEKYPIPSTMCTGKREKCVIQKENNFKQPGDRYRS   437
Vitis_vinifera_1                          .EVDYFPSRFDPVRHAETFPIPSTICHGKREKVVIEKENNFKQPGERYRS   437
Nicotiana_sylvestris                      .EIDYFPSRYDPVRHAEKYPIPSTMCTGKREKCVIQKENNFKQAGDRYRS   437
Vitis_vinifera_2                          .EVDYFPSRFDPVRHAETFPIPSTICHGKREKVVIEKENNFKQPGERYRS   437
Prunus_avium                              .EVNYFPSRYDPVRHAERFPIPSNILTGKRERRVIEKENNFKQPGERYRS   437
Vitis_vinifera_3                          .EVDYFPSRFDPVRHAETFPIPSTICHGKREKVVIEKENNFKQPGERYRS   437
Beta_vulgaris_subsp._maritima             .EVDYFPSRYDPVRHAERHPIPSNVINGKRERQIIAKENNFKQPGERYRS   437
Nicotiana_tomentosiformis                 .EIDYFPSRYDPVRHAEKYPIPSTMCTGKREKCVIQKENNFNQPGERYRS   437
Solanum_tuberosum                         .EIDYFPSRYDQVRHAEVYPIPSTVCSGKREKCIIQKENNFKQPGERYRT   437
Eucalyptus_grandis_1                      .EINYFPSKFDPVRHAERYPIPSQIVTGKREKVIIEKENNFKQPGERYRS   437
Solanum_lycopersicum                      .EIDYFPSRYDQVRHAEVYPIPSTVCSGKREKCIIQKENNFKQPGERYRS   437
Tarenaya_hassleriana_1                    .EVNYFPSRYDPVRHAERYPIPPVICSGRRERCIIEKENNFKQPGERYRS   437
Vitis_vinifera_4                          .EVNYFPSRYDPVRHAERYPIPPAILTGRREKCIIPKENNFKQPGERYRS   437
Musa_acuminata_subsp._malaccensis         .EVNYFPSRYDPVRHAERFPIPSSVVTGRREKSIINKENNFKQPGERYRS   437
Theobroma_cacao_1                         .EVNYFPSRFDPVRHAEKYPIPTAICSGKREKCIIDKENNFQQPGERYRS   437
Sesamum_indicum_1                         .EVNYFPSRYDPTRHSEMYPIPPVVLSGKREKRCIEKENNFKQPGERYRS   437
Brassica_juncea_1                         .EVNYFPSRYDPVRHAEKYPTPPAVCSGKRERCIIEKENNFKEPGERYRS   437
Hylocereus_undatus                        .EIDYFPSKFDPARQAEKYPILTKVYTGKREKTVIPKENNFKQPGDRYRS   437
Camelina_sativa_1                         .EVNYFPSRYDQVRHAEKYPAPPAVCSGKRERCVIAKENNFKEPGERYRS   437
Camelina_sativa_2                         .EVNYFPSRYDPVRHAEKYPAPPAVCSGTRERCVIAKENNFKEPGERYRS   437
Ipomoea_batatas_1                         .EVDYFPSKFDNTRNAERFPTPLRIVTGQRDKCVIEKENNFKQPGDRYRS   437
Raphanus_sativus_1                        .EVNYFPSRYDPVRHAEKYPTPPAVCSGKRERCIIEKENNFKEPGERYRS   437
Ipomoea_batatas_2                         .EVDYFPSKFDNTRNAERFPTPLRIVTGQRDKCVIEKENNFKQPGDRYRS   437
Camelina_sativa_3                         .EVNYFPSRYDQVRHAEKYPAPPAVCSGKRERCVIAKENNFKEPGERYRS   437
Rheum_australe                            .EVNYFPSRFDPARHAEKYPIPQNVLNGRREKQIIAKENNFEEPGVRYRS   437
Brassica_juncea_2                         .EVNYFPSRYDPVRHAEKYPTPPAVCSGKRERCVIEKENNFKEPGERYRS   437
Ziziphus_jujuba                           .EVNYFPSRYDPVRHAEQHPIPSAIFNGKREKCIIEKENNFKQPGERYRS   437
Tarenaya_hassleriana_2                    .EVNYFPSRYDPVRHAERYPVPSVVCSGRRERCIIEKENNFKQPGERYRS   437
Brassica_rapa                             .EVNYFPSRYDPVRHAEKYPTPPAVCSGKRERCVIEKENNFKEPGERYRS   437
Jatropha_curcas_1                         .EVNYFPSRYDPVRHAEKYPIPPPVLTGRREKTVIPKENNFKQPGERYRS   437
Nicotiana_glutinosa                       .EIDYFPSRYDPVRHAEKYPIPSTMCTGKREKCVILKENNFKQPGERYRS   437
Brassica_juncea_3                         .EVNYFPSRYDPVRHAEKYPTPPAICSGKRERCIIEKENNFKEPGERYRS   437
Arabis_alpina                             .EVNYFPSRYDTVRHAEKYPAPPAVCSGKRERCVIEKENNFKEPGERYRS   437
Eucalyptus_grandis_2                      .EINYFPSKFDPVRHAERYPIPSQIVTGKRERVIIEKENNFKQAGERYRS   437
Theobroma_cacao_2                         .EINYFPSRYDPVRHAERFPIPPAVCSGRREKCIIEKENNFKQPGERYRS   437
Arabidopsis_thaliana_1                    .EVNYFPSRYDQVRHAEKYPTPPAVCSGKRERCIIEKENNFKEPGERYRT   437
Brassica_oleracea                         .EVNYFPSRYDPVRHAEKYPTPPAVCSGKRERCVIEKENNFKEPGERYRS   437
Arabidopsis_thaliana_2                    .EVNYFPSRYDQVRHAEKYPTPPAVCSGKRERCVIEKENNFKEPGERYRT   437
Brassica_napus                            .EVNYFPSRYDPVRHAEKYPTPPAVCSGKRERCIIEKENNFQQPGERYRS   437
Sesamum_indicum_2                         .EVNYFPSRYDPSRHAERYPIPPVVLSGKRGKICIEKENNFKQPGDRYRS   437
Solanum_melongena                         .EVDYLPSRFDPCRPAEQYPIPSCVLTGRREKCVIPKENNFKQAGERYRT   437
Sesamum_indicum_3                         .EVNYFPSRYDPSRHAERYPIPPVVLSGKRGKICIEKENNFKQPGDRYRS   437
Raphanus_sativus_2                        .EVNYFPSRYDPVRHAEKYPTPPAVCSGKRERCIIEKENNFKEPGERYRS   436
Genlisea_aurea                            .EVNYFPSRYDPVRHADKVPIPSVPFSGRREKVSIEKENNFKQPGERYRS   437
Arabidopsis_thaliana_3                    .EVNYFPSRYDQVRHAEKYPTPPAVCSGKRERCIIEKENNFKEPGERYRT   437
Gossypium_arboreum_1                      .EINYFPSRYDPVRHAEMFPIPPAVCTGRREKCIIEKENNFKQPGERYRS   437
Gossypium_hirsutum                        .EINYFPSRYDPVRHAEMFPIPPAVCTGRREKCIIEKENNFKQPGERYRS   437
Eriobotrya_japonica                       .EVDYFPSRYQPARHAETFPIPSNIITGKRDKCVIEKENNFKQPGERYRS   437
Gossypium_raimondii                       .EINYFPSRYDPVRHAEMFPIPPAVCTGRREKCIIEKENNFKQPGERYRS   437
Tarenaya_hassleriana_3                    .EVNYFPSRFDPVRHAERFPSPPAICSGRREKCIIEKENNFKQPGERYRS   437
Jatropha_curcas_2                         .EIDYFQSRYDPVRHAERYPIPNAIISGRREKCVIPKENNFKQPGERYRS   437
Hevea_brasiliensis                        .EVNYFPSRYDPVRHAEKYPIPSAILSGNCEKCVIPKENNFKQPGERYRS   437
Arabidopsis_thaliana_4                    .EVNYFPSRYDQVRHAEKYPTPPAVCSGKRERCIIEKENNFKEPGERYRT   437
Elaeis_guineensis                         .EVNYFPSRYDPVRHAERFPIPSRILTGRREKCVIHKENNFKQPGERYRS   437
Gossypium_arboreum_2                      .EVNYFPSRYDPVRHAEKHPIPSTVLSGKREKCIIGKENNFKQPGERYRS   437
Prunus_mume_2                             .EVNYFPSRYDPVRNAERFPIPPNILSGKREKCIIEKENNFKQPGERYRS   437
Citrus_sinensis                           .EIDYFPSRYDPVRHAEMHPTPPEICGGRREKCIIPKENNFKQPGERYRS   437
Brassica_juncea_4                         .EVNYFPSRYDPVRHAEKYPTPPAVCSGKRERCVIEKENNFKEPGERYRS   437
Consensus                                     y                      g           n       y
```

```
Amaranthus_cruentus_cv._Hopi_Red_Dye      WDPA....RQERFIGRFVKALSDPRLTYEIRNIWVSWLSQADKSLGMKVA    483
Bos_taurus                                VLNE...EQRKRLCENIAGHLKDAQLFIQKKAVKN..FSDVHPEYGSRIQ    494
Homo_sapiens                              VLNE...EQRKRLCENIAGHLKDAQIFIQKKAVKN..FTEVHPDYGSHIQ    494
Beta_vulgaris_subsp._vulgaris_1           WDPA....RQDRFVGRFVKALSDPRLTYEIRSIWVSYLTQADKSLGMKVA    483
Mesembryanthemum_crystallinum|root        WDPA....RQERYLARWVKALSDPKVTPEIRGVWVSWLSQSDRSLGMKLA    484
Mesembryanthemum_crystallinum|leaf        WDPA....RQERFICRWVDALSDPRITHEIRNIWISYWSQADKSVGMKLA    483
Prunus_mume_1                             WAPD....RQERFIRRWVDALSDPRVTHEIRSIWISYWSQADKSLGQKLA    483
Gardenia_jasminoides                      WAPD....RQERFVRRWVDALSDPRLTHEIRSIWISYWSQADKSLGQKIA    483
Bassia_scoparia                           MDPA....RQERFICRVVDALSDPRITHEIRSIWISYWTQADKSLGMKVA    483
Beta_vulgaris_subsp._vulgaris_2           MDPA....RQERFICRVVDALSDPRITHEIRSIWVSYWSQADKSLGMKVA    483
Suaeda_salsa                              MDPA....RQERFICRVVDALSDPRVTHEIRSIWISYWSQADKSLGMKVA    483
Nicotiana_tabacum_1                       FTPD....RQERFIRRWVEALSDPRITYEIRSIWISYWSQADKSLGQKLA    483
Nicotiana_tabacum_2                       FTPD....RQERFIRRWVEALSDPRITYEIRTIWISYWSQADKSLGQKLA    483
Prunus_persica                            WAPD....RQERFIRRWVDALSDPRVTHEIRSIWISYWSQADKSLGQKLL    483
Nicotiana_benthamiana                     FTPD....RQERFIRRWVEALSDPRITYEIRSIWISYWSQADKSLGQKLA    483
Vitis_vinifera_1                          FAPD....RQERFINRWVETLSDPRVTYEIRSIWISYWSQADRSLGQKLA    483
Nicotiana_sylvestris                      FTPD....RQERFIRRWVEALSDPRITYEIRSIWISYWSQADKSLGQKLA    483
Vitis_vinifera_2                          FAPD....RQERFINRWVETLSDPRVTYEIRSIWISYWSQADRSLGQKLA    483
Prunus_avium                              WAPD....RQERFIRRCVDALSDPRVSHEIRSIWISYWSQADKSLGQKIA    483
Vitis_vinifera_3                          FAPD....RQERFINRWVETLSDPRVTYEIRSIWISYWSQADRSLGQKLA    483
Beta_vulgaris_subsp._maritima             MDPA....RQERFICRVVDALFDPRITHEIRSIWVSYWFQADKSLGMKVA    483
Nicotiana_tomentosiformis                 FTPD....RQERFIRRWVEALSDPRITYEIRSIWISYWSQADKSLGQKLA    483
Solanum_tuberosum                         FTPD....RQERFIRRWVEALSDPRITYEIRSIWITYWSQADKSLGQKLA    483
Eucalyptus_grandis_1                      WAPD....RQERFINRWIKALTEPRVTHEHRSIWISYWTQADKSLGQKLA    483
Solanum_lycopersicum                      FTPD....RQERFIRRWVEALSDPRITYEIRSIWITYWSQADKSLGQKLA    483
Tarenaya_hassleriana_1                    FTPD....RQERFIRRWIEALSDPRITHEIRSIWISYWSQADQSLGQKLA    483
Vitis_vinifera_4                          FAPD....RQERFIQRWVDALSDPRVTYEIRSIWISYWSQADKSLGQKLA    483
Musa_acuminata_subsp._malaccensis         WAPD....RQERFVHRWVDALSDPRVTHEIRDIWISYWSQCDKSLGQKIA    483
Theobroma_cacao_1                         FAPD....RQERFIYRWVDALSDPRVTYEIRSIWISYWSKADKSLGQKLA    483
Sesamum_indicum_1                         WAPD....RQERFIRRWVEALSDPRLTHEIRSIWISYWSQADKSLGQKLA    483
Brassica_juncea_1                         FTPE....RQERFIGRWIDALSDPRITHEIRSIWISYWSQADKSLGQKLA    483
Hylocereus_undatus                        WDPA....RQERFVKRWVDVLSDPRVTLELRSIWVSYLSQCDRSLGMKVA    483
Camelina_sativa_1                         FPPE....RQERFICRWIDALSDPRITHEIRSIWISYWSQADKSLGQKLA    483
Camelina_sativa_2                         FPPE....RQERFICRWIDALSDPRITHEIRSIWISYWSQADKSLGQKLA    483
Ipomoea_batatas_1                         WAPD....RQDRFINRWVKALSEPRVTHEIRSTWISYLTQADRSLGQKVA    483
Raphanus_sativus_1                        FTPE....RQERFIGRWIDALSDPRITHEIRSIWISYWSQADKSLGQKLA    483
Ipomoea_batatas_2                         WAPD....RQDRFINRWVKALSEPRVTHEIRSTWISYLTQADRSLGQKVA    483
Camelina_sativa_3                         FPPE....RQERFICRWIDALSDPRITHEIRSIWISYWSQADKSLGQKLA    483
Rheum_australe                            FDPA....RQERFICRWIGALSDPRVTPEIRSIWISYWSQADKSLGMKLA    483
Brassica_juncea_2                         FTPE....RQERFIRRWIEALSDPRITHEIRSIWISYWSQADKTLGQKLA    483
Ziziphus_jujuba                           WAPD....RQERFIRRWIDALSDPRVTHEIRSIWISYWSQADKSLGQKLA    483
Tarenaya_hassleriana_2                    FTPD....RKERFIRRWIEALSDPRITHEIRSIWISYWSQADQSLGQKLA    483
Brassica_rapa                             FTPE....RQERFIGRWIDALSDPRITHEIRSIWISYWSQADKSLGQKLA    483
Jatropha_curcas_1                         FTPD....RKDRFIRRWVEALSDPRVTYEIRSIWISYWSQADKSLGQKIA    483
Nicotiana_glutinosa                       FTPD....RQERFIRRWVEALSDPRITHEIRSIWISYWSQADKSLGQKLA    483
Brassica_juncea_3                         FTPE....RQERFIGRWIDALSDPRITHEIRSIWISYWSQADKSLGQKLA    483
Arabis_alpina                             FPPE....RQERFIRRWIEALSDPRITHEIRSIWISYWSQADKSLGQKLA    483
Eucalyptus_grandis_2                      WAPD....RQERFINRWIKALSEPRVTHEHRSIWISYWTQADKSIGQKLA    483
Theobroma_cacao_2                         WAPD....RQERFICRWVDALSDPRVTHEIRSIWITYWSQACKSLGQKLA    483
Arabidopsis_thaliana_1                    FTPE....RQERFIQRWIDALSDPRITHEIRSIWISYWSQADKSLGQKLA    483
Brassica_oleracea                         FTPE....RQERFIGRWIDALSDPRITHEIRSIWISYWSQADKSLGQKLA    483
Arabidopsis_thaliana_2                    FTPE....RQERFIQRWIDALSDPRITHEIRSIWISYWSQADKSLGQKLA    483
Brassica_napus                            WDAD....RQERFVKRFVEALAEPRVTHEIRSIWISYWTQADKSLGQKLA    483
Sesamum_indicum_2                         WAPD....RQERFIRRWVEALSDPRLTHEIRSIWVSYWSQADKSLGQKLA    483
Solanum_melongena                         WEPD....RQDRYINKWVESLSDPRVTHEIRSIWISYLSQADKSCGQKVA    483
Sesamum_indicum_3                         WAPD....RQERFIRRWVEALSDPRLTHEIRSIWVSYWSQADKSLGQKLA    483
Raphanus_sativus_2                        FTPE....RQERFIGRWIDALSDPRITHEIRSIWISYWSQADKSLGQKLA    482
Genlisea_aurea                            WDPD....RQERFIRRWVDALSDPRLTQEIRSIWISYWTQADKSLGQKLA    483
Arabidopsis_thaliana_3                    FTPE....RQERFIQRWIDALSDPRITHEIRSIWISYWSQADKSLGQKLA    483
Gossypium_arboreum_1                      WAAD....RQERFICRWVEAFSDPRVTHEIRSIWISYWSQADKSLGQKLA    483
Gossypium_hirsutum                        WAAD....RQERFICRWVEALSDPRVTHEIRSIWISYWSQADKSLGQKLA    483
Eriobotrya_japonica                       WAPD....RQERFLHRWVDVLSEPRVTYEIRSIWISYWSQADRSFGQKLA    483
Gossypium_raimondii                       WAAD....RQERFICRWVDALSDPRVTHEIRSIWISYWSQADKSVGQKLA    483
Tarenaya_hassleriana_3                    WAPD....RQERFLKRWVEALSDKRCTHEIRSIWISYWSQADKSLGQKLA    483
Jatropha_curcas_2                         WTPD....RQERFLCRLVNALSDPRITHEIRGIWVSYWSQCDQSLGQKLA    483
Hevea_brasiliensis                        FSSD....RKERFIRRWVEAMSDPRVTYEIRSIWISYWYQADKSLGQKIA    483
Arabidopsis_thaliana_4                    FTPE....RQERFIQRWIDALSDPRITHEIRSIWISYWSQADKSLGQKLA    483
Elaeis_guineensis                         WDPN....RQERFIRRWVEALSDPRVTHEIQSIWISYWSQCDKSLGQKIA    483
Gossypium_arboreum_2                      FSAD....RQERFINRWIDALSDPRVTHEIRSIWISYWSQADKSLGQKIA    483
Prunus_mume_2                             WAPD....RQERFICRWVDALSDPRVTHEIRSIWISYWSQADKSLGQKLA    483
Citrus_sinensis                           WAPD....RQERFICRWVDALSDPRVTHEIRHIWISYWSQACKSLGQKLA    483
Brassica_juncea_4                         FTPESFDFRQERFIRRWIEALSDPRITHEIRSIWISYWSQADKTLGQKLA    487
Consensus                                             r                                  g
```

Amaranthus_cruentus_cv._Hopi_Red_Dye	SRLNIRPTM........................	492
Bos_taurus	ALLDKYNEEKPKNAVHTYVQHGSHLSAREKANL	527
Homo_sapiens	ALLDKYNAEKPKNAIHTFVQSGSHLAAREKANL	527
Beta_vulgaris_subsp._vulgaris_1	SRLNIRPTM........................	492
Mesembryanthemum_crystallinum|root	NKLNVRPTM........................	493
Mesembryanthemum_crystallinum|leaf	SRLNVRPTM........................	492
Prunus_mume_1	SRLNVRPSI........................	492
Gardenia_jasminoides	FRLNVRPTM........................	492
Bassia_scoparia	SRLNVKATM........................	492
Beta_vulgaris_subsp._vulgaris_2	SRLNVRPTM........................	492
Suaeda_salsa	SRLNVRPTM........................	492
Nicotiana_tabacum_1	SRLNVRPSI........................	492
Nicotiana_tabacum_2	SRLNVRPSI........................	492
Prunus_persica	SRLNVRPSI........................	492
Nicotiana_benthamiana	SRLNVRPSI........................	492
Vitis_vinifera_1	SRLNVKPKY........................	492
Nicotiana_sylvestris	SRLNVRPSI........................	492
Vitis_vinifera_2	SRLNVKPKY........................	492
Prunus_avium	TRLNVRPSI........................	492
Vitis_vinifera_3	SRLNVKPKY........................	492
Beta_vulgaris_subsp._maritima	SRLNVRPTM........................	492
Nicotiana_tomentosiformis	SRLNVRPSI........................	492
Solanum_tuberosum	SRLNVRPSI........................	492
Eucalyptus_grandis_1	SRLSARPSM........................	492
Solanum_lycopersicum	SRLNVRPSI........................	492
Tarenaya_hassleriana_1	SRLNVRPSI........................	492
Vitis_vinifera_4	SRLNVRPSI........................	492
Musa_acuminata_subsp._malaccensis	TRLNVKPTM........................	492
Theobroma_cacao_1	SRLSVRPSI........................	492
Sesamum_indicum_1	SRLNVRPTM........................	492
Brassica_juncea_1	SRLNVRPNI........................	492
Hylocereus_undatus	TRLNMRPTM........................	492
Camelina_sativa_1	SRLNVRPSI........................	492
Camelina_sativa_2	SRLNVRPSI........................	492
Ipomoea_batatas_1	SRLNIRPTM........................	492
Raphanus_sativus_1	SRLNVRPSI........................	492
Ipomoea_batatas_2	SRLNIRPTM........................	492
Camelina_sativa_3	SRLNVRPSI........................	492
Rheum_australe	NRLNVKPTM........................	492
Brassica_juncea_2	SRLNVRPSI........................	492
Ziziphus_jujuba	SRLNVRPSI........................	492
Tarenaya_hassleriana_2	SRMNVRPNI........................	492
Brassica_rapa	SRLNVRPNI........................	492
Jatropha_curcas_1	SHLNMRPTM........................	492
Nicotiana_glutinosa	SRLNVRPSI........................	492
Brassica_juncea_3	NRLNVRPSI........................	492
Arabis_alpina	SRLNVRPSI........................	492
Eucalyptus_grandis_2	SSLSARPSM........................	492
Theobroma_cacao_2	SRLNVKPNI........................	492
Arabidopsis_thaliana_1	SRLNVRPSI........................	492
Brassica_oleracea	SRLNVRPSI........................	492
Arabidopsis_thaliana_2	SRLNVRPSI........................	492
Brassica_napus	SRLNVRPKY........................	492
Sesamum_indicum_2	SRLNVRPAM........................	492
Solanum_melongena	SRLLVKPTM........................	492
Sesamum_indicum_3	SHLNVRPTM........................	492
Raphanus_sativus_2	SRLNVRPSI........................	491
Genlisea_aurea	SRLNVRPTM........................	492
Arabidopsis_thaliana_3	SRLNVRPSI........................	492
Gossypium_arboreum_1	SRLNVRPSI........................	492
Gossypium_hirsutum	SRLNVRPSI........................	492
Eriobotrya_japonica	TRLNVRPSI........................	492
Gossypium_raimondii	SRLNVRPSI........................	492
Tarenaya_hassleriana_3	SRLNVRPSF........................	492
Jatropha_curcas_2	SRLNVRPNI........................	492
Hevea_brasiliensis	SHLNMRPTM........................	492
Arabidopsis_thaliana_4	SRLNVRPSI........................	492
Elaeis_guineensis	TRLNVKPCM........................	492
Gossypium_arboreum_2	SRLNVRPSI........................	492
Prunus_mume_2	SRLNVRPSI........................	492
Citrus_sinensis	SRLNVRPNI........................	492
Brassica_juncea_4	SRLNVRPSI........................	496
Consensus		
